# Network-level encoding of local neurotransmitters in cortical astrocytes

**DOI:** 10.1038/s41586-024-07311-5

**Published:** 2024-04-17

**Authors:** Michelle K. Cahill, Max Collard, Vincent Tse, Michael E. Reitman, Roberto Etchenique, Christoph Kirst, Kira E. Poskanzer

**Affiliations:** 1 Department of Biochemistry & Biophysics, University of California, San Francisco, CA USA; 2 Neuroscience Graduate Program, University of California, San Francisco, CA USA; 3 Departamento de Química Inorgánica, Analítica y Química Física, INQUIMAE, Facultad de Ciencias Exactas y Naturales, Universidad de Buenos Aires, CONICET, Buenos Aires, Argentina; 4 Department of Anatomy, University of California, San Francisco, CA USA; 5 Kavli Institute for Fundamental Neuroscience, San Francisco, CA USA; 6 Lawrence Berkeley National Laboratory, Berkeley, CA USA

**Keywords:** Astrocyte, Cellular neuroscience

## Abstract

Astrocytes, the most abundant non-neuronal cell type in the mammalian brain, are crucial circuit components that respond to and modulate neuronal activity through calcium (Ca^2+^) signalling^1–7^. Astrocyte Ca^2+^ activity is highly heterogeneous and occurs across multiple spatiotemporal scales—from fast, subcellular activity^3,4^ to slow, synchronized activity across connected astrocyte networks^8–10^—to influence many processes^5,7,11^. However, the inputs that drive astrocyte network dynamics remain unclear. Here we used ex vivo and in vivo two-photon astrocyte imaging while mimicking neuronal neurotransmitter inputs at multiple spatiotemporal scales. We find that brief, subcellular inputs of GABA and glutamate lead to widespread, long-lasting astrocyte Ca^2+^ responses beyond an individual stimulated cell. Further, we find that a key subset of Ca^2+^ activity—propagative activity—differentiates astrocyte network responses to these two main neurotransmitters, and may influence responses to future inputs. Together, our results demonstrate that local, transient neurotransmitter inputs are encoded by broad cortical astrocyte networks over a minutes-long time course, contributing to accumulating evidence that substantial astrocyte–neuron communication occurs across slow, network-level spatiotemporal scales^12–14^. These findings will enable future studies to investigate the link between specific astrocyte Ca^2+^ activity and specific functional outputs, which could build a consistent framework for astrocytic modulation of neuronal activity.

## Abstract (web summary)

A study investigates subcellular, single-cell and network-level comunication within the astrocyte network in response to the two major neurotransmitter inputs.

## Main

A set of defined rules governing neuronal input–output relationships is a cornerstone of cellular neuroscience. Given a specific excitatory or inhibitory neurotransmitter (NT) input, post-synaptic membrane potential changes that lead to action potentials can be accurately predicted. However, neurons are not the only nervous system cells that sense NTs. Astrocytes—the most abundant non-neuronal cell type in the mammalian brain—are crucial circuit components that respond to and modulate neuronal activity through Ca^2+^ signalling^1–7^. However, the set of rules governing input–output relationships in astrocytes is poorly defined, in part because it is unclear over which spatiotemporal scales these relationships should be evaluated. Although there seem to be fast and local astrocytic responses to local stimuli^3,4^, there is also evidence to suggest that astrocyte responses to local stimuli have a spatiotemporally distributed component, as local astrocyte stimulation can lead to distributed changes in neuronal activity and plasticity^15,16^. Thus, a comprehensive framework describing input–output relationships in astrocytes requires simultaneous investigation of activity across multiple spatiotemporal scales.

Here we set out to build an input framework governing transient and sustained cortical astrocyte Ca^2+^ activity at three spatial scales: subcellular, single cell and network. To take a physiologically relevant and comparative approach, we focused on astrocyte responses to the two main NTs: glutamate and GABA (γ-aminobutyric acid). Whereas previous studies demonstrate general astrocyte Ca^2+^ increases in response to these NTs^2,6,17^, our goal was to link specific excitatory and inhibitory chemical inputs to specific astrocyte Ca^2+^ activity, and map the scales over which astrocytes could exert effects on neuronal circuitry.

## NTs drive distinct astrocyte activity

**Fig. 1:**
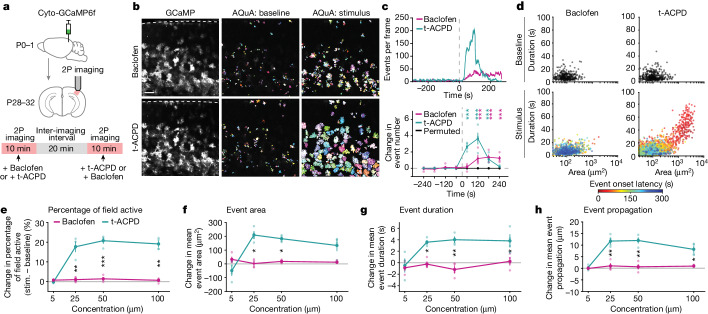
Direct GABAergic and glutamatergic receptor activation drive distinct astrocyte Ca^2+^ activity. **a**, Experimental strategy for cyto-GCaMP6f expression and two-photon (2P) imaging of astrocytic Ca^2+^ in acute V1 cortical slices during pharmacological activation through bath-application. Receptor agonists sequentially bath-applied to the same slice, with an inter-imaging interval of >20 min, including >10-min washout period. P0, postnatal day 0. **b**, Left: representative astrocytic GCaMP6f fluorescence during bath-application of baclofen (top) and t-ACPD (bottom). Dashed line: pia. Middle and right: all AQuA-detected events 300 s before (middle) and after (right) agonist addition (50 µM). Scale bar, 100 μm. **c**, Top: representative traces (AQuA events per frame) of FOV in **b**. Bottom: average change from baseline in events per minute. Periods of 300–0 s before and 0–240 s after agonist addition were used to calculate change in events per 60 s per active astrocyte (≥1 AQuA-detected event). Data shown by slice (*n* = 4 slices stimulated with 50 μM agonist); mean ± s.e.m. Permutation test used to determine significance. *P* values in Supplementary Table 1. 0 s: time of agonist addition. **d**, Features of individual Ca^2+^ events at baseline (top, black) and after bath-application of baclofen (bottom left) or t-ACPD (bottom right). Events following agonist addition colour-coded by onset time. Dots: individual Ca^2+^ events from *n* = 4 slices stimulated with 50 µM agonist. **e**–**h**, Average change in Ca^2+^ features with bath-application of baclofen (pink) or t-ACPD (green) at four concentrations. Agonist order alternated between conditions: baclofen added first at 5 and 50 μM and second at 25 and 100 μM. Change calculated by comparing 120 s before and after agonist entry. Data shown by slice (*n* = 4 slices, 4 mice for each concentration); mean ± s.e.m. Paired *t*-tests between agonists at each concentration followed by Bonferroni–Holm correction with family-wise error rate ≤ 0.05. *P* values in Supplementary Table 2. All statistical tests are two-sided. NS: *P* ≥ 0.05; *P < 0.05; **P < 0.01; ***P < 0.001.

**Extended Data Fig. 1:**
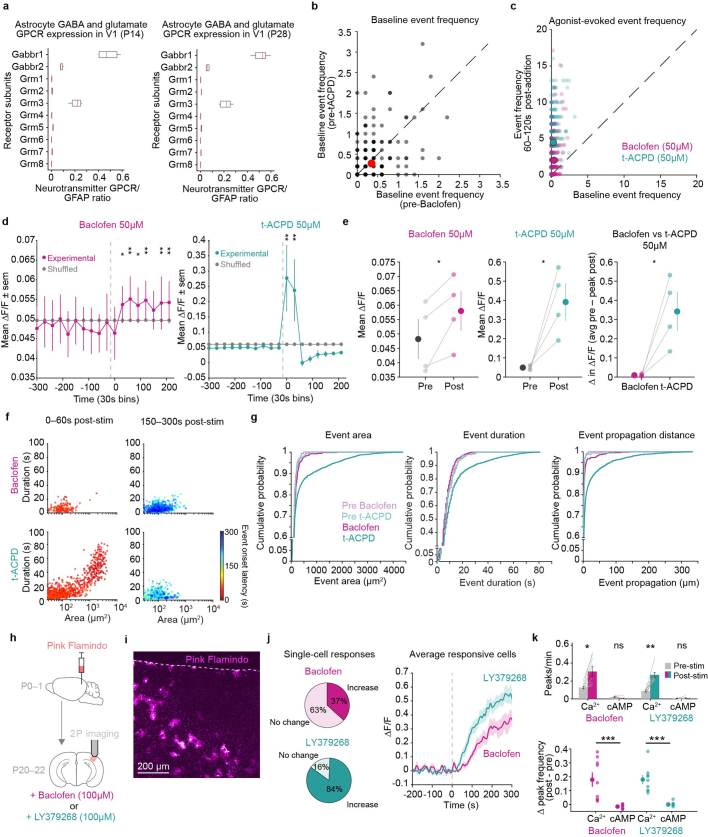
Different responses to activation of astrocytic glutamatergic and GABAergic receptors via pharmacological bath-application. **a**, Ribosomal-mRNA expression in visual cortex astrocytes of P14 (n = 4 biological replicates) and P28 (n = 5 biological replicates) mice from the Farhy-Tselnicker et al. publicly available dataset (NCBI Gene Expression Omnibus, GSE161398). Visual cortex astrocytes show expression of GABA_B_ receptors and mGluR_3_, but low expression of all other mGluRs, including mGluR_5_ (ref. ^22^). Similar expression levels are found in the Srinivasan et al. dataset available at http://astrocyternaseq.org/. Ratio of FPKM for the gene of interest / FPKM for GFAP were calculated to normalize for potential differences in the sequencing depth of replicates. Center line: median; box limits: 25^th^ and 75^th^ percentiles; whiskers: minimum and maximum values. **b**, Baseline event frequency (events/60 s) for each active astrocyte prior to bath application of baclofen (50 µM, x-axis) and t-ACPD (50 µM, y-axis). Data shown by astrocyte (grey dots, from n = 4 slices) and mean (red dot). Dashed line = unity line. Baseline event frequencies prior to baclofen and t-ACPD application were compared for each astrocyte using a paired two-sided t-test (*p* = 0.14). **c**, Event frequency (events/60 s) for each active astrocyte 300–0 s before and 60–120 s after addition of agonist (50 µM). Data shown by astrocyte (light dots, from n = 4 slices) and mean (solid dots) for baclofen (pink) and t-ACPD (green). Dashed line = unity line; all astrocytes above the unity line display increased activity in presence of agonist. For b & c, 300–0 s before addition of agonist was used to calculate mean baseline event frequency per astrocyte; an active astrocyte is any cell with ≥ 1AQuA-detected event. Note the difference in axes between graphs in b & c, reflecting the low baseline event frequency for all astrocytes. **d**, Time-series traces of average ΔF/F in 30 s windows from active cells in each slice. 300–0 s before and 0–240 s after bath-application of agonist used to calculate event average ΔF/F /30 s. Data shown as mean ± sem (n = 4 slices, 4 mice stimulated with 50 μM agonist). Permutation test used to determine significance. One-sided *p*-values for all timepoints are in Supplementary Table 3. 0 s = frame of agonist entry into the imaging chamber. ΔF/F values were calculated using a moving 10 s baseline window, averaging the lower 50% of values in the window. Active cells were cells with ≥ 1 AQuA event detected in either the baclofen or t-ACPD recording. **e**, Left and center: Average ΔF/F before and after bath-application of baclofen (50 μM, left) and t-ACPD (50 μM, center). ΔF/F after bath-application of agonist is from the 30 s time window with the highest average ΔF/F for each slice (“peak post”). Right: Change in average ΔF/F after bath-application of agonist. Data shown as slices (light dots and grey lines, n = 4 slices, 4 mice) and mean ± sem (dark dots and error bars). Two-sided paired t-test compares conditions. Baclofen: *p* = 0.046, t-ACPD: *p* = 0.031 and Δ in ΔF/F: *p* = 0.033. **f**, Scatter plots of the area and duration of individual Ca^2+^ events 0–60 s (left) and 150–300 s (right) after bath-application of baclofen (top) or t-ACPD (bottom). Separating events into these two time-windows highlights events occurring early that are covered in Fig. 1d by those with longer onset latencies. Events following bath-application of agonists color-coded by onset time. Dots represent individual Ca^2+^ events from n = 4 slices stimulated with 50 µM agonist. Note: these are the same data, with the same onset latency color scale, as shown in Fig. 1d, bottom. **g**, Distributions of event area, duration and propagation 120–0 s before (“Pre”) or 0–120 s after addition of baclofen (50 μM) or t-ACPD (50 μM). One-way ANOVA followed by Tukey-Kramer Test determine significant pairwise comparisons between conditions. *p-*values in Supplementary Table 4. Note that, for all features, pre-baclofen, pre-tACPD, and baclofen events are not significantly different from one another. Only events following addition of t-ACPD show a rightward shift for all features. **h**, Experimental strategy for Pink Flamindo expression and 2 P imaging of astrocytic cAMP in acute cortical slices. **i**, Representative Pink Flamindo fluorescence in V1 FOV; dotted line denotes pia. **j**, Left: Percent of total astrocytes that increase fluorescence or show no change with bath-application of baclofen (top, pink) or mGluR_3_-specific agonist LY379268 (bottom, green) (*n* = 147 astrocytes) in the presence of TTX and CBX. Right: Average ∆F/F trace only from responsive cells in each slice (mean ± sem across slices from *n* = 54 responsive astrocytes [baclofen] and 123 responsive astrocytes [LY379268] from 8 slices, 3 mice). To capture steady-state changes, ∆F/F values were calculated using raw – background fluorescence and a fixed baseline window (frames 1–100), then lowpass filtered at 0.01 Hz. **k**, Top: Average Ca^2+^ or cAMP peaks/minute/astrocyte before and after bath-application of baclofen (pink) or LY379268 (green). Data shown as slices (grey lines) and corresponding mean ± sem. Two-sided paired t-test compares pre- and post-agonist values for each condition. *P*-values corrected for multiple comparisons using Bonferroni-Holm correction FWER ≤ 0.05. Baclofen: *p* = 0.019 (Ca^2+^) and 0.057 (cAMP). LY379268: *p* = 0.0017 (Ca^2+^) and 0.66 (cAMP). Bottom: Average change in Ca^2+^ or cAMP peaks/minute following bath-application of baclofen (pink) or LY379268 (green). Data shown by slice (light dots) and corresponding mean ± sem (dark dots and error bars). Two-sided rank sum tests compare Ca^2+^ and cAMP frequency changes for each agonist. *p* = 0.000082 (baclofen) and 0.000082 (LY379268). Cyto-GCaMP: *n* = 809 active astrocytes (baclofen) and 1033 active astrocytes (LY379268), 9 slices, 3 mice. Pink Flamindo: *n* = 147 astrocytes, 8 slices, 3 mice. To detect transient fluctuations, ∆F/F was calculated using a moving 10 s baseline window, with peaks determined for each astrocyte if ∆F/F ≥ 3 SD above mean baseline ∆F/F.

To first test whether astrocytes show generally distinct activity in response to different NTs, we used two-photon astrocyte Ca^2+^ imaging (using the genetically encoded intracellular indicator cyto-GCaMP6f) while sequentially bath-applying GABA and glutamate receptor agonists onto ex vivo acute cortical slices from mice (Fig. 1a). We activated the GABAergic and glutamatergic G-protein-coupled receptors (GPCRs) expressed by astrocytes^18,19^ (Extended Data Fig. 1a and Supplementary Videos 1 and 2), using baclofen to activate GABA_B_ receptors (GABA_B_Rs)^2,17,20^ and a broad-spectrum metabotropic glutamate receptor (mGluR) agonist, (1*S*-3*R*)-ACPD (t-ACPD)^15,21^, to activate mGluR_3_, the mGluR subtype expressed by astrocytes at this age^22^, while silencing neuronal firing with tetrodotoxin. We analysed the resulting Ca^2+^ activity using the event-detection software AQuA^8^ (Fig. 1b). In the same populations of astrocytes, with similar levels of baseline activity (Extended Data Fig. 1b), GABA_B_R or mGluR_3_ activation increased Ca^2+^ event frequency, but each led to Ca^2+^ responses that differed in time course and magnitude. Using both event-based and region-of-interest (ROI)-based analyses, we found that t-ACPD induced robust, transient Ca^2+^ activity increases, whereas baclofen caused a delayed and prolonged activation, lasting to the end of recording (Fig. 1c and Extended Data Fig. 1c–e). Analysing individual Ca^2+^ events by area and duration, we found a population of events that were larger and longer compared to the baseline with t-ACPD, but not with baclofen (Fig. 1d and Extended Data Fig. 1f,g). To ensure that these distinct responses were not dependent on a specific agonist concentration or order, we quantified activity across a broad concentration range, alternating agonist order between concentrations. Across Ca^2+^ event features, we saw a consistently higher response with mGluR_3_ compared to GABA_B_R activation (Fig. 1e–h), demonstrating that the same cortical astrocyte populations exhibit distinct activity, with distinct time courses, in response to different NTs.

GABA_B_R and mGluR_3_ are both G_i_-coupled GPCRs canonically linked to decreases in intracellular cyclic adenosine monophosphate (cAMP). To explore whether these two G_i_-GPCRs also engage cAMP in NT-specific ways, we expressed the genetically encoded cAMP sensor Pink Flamindo^23^ in astrocytes, and bath-applied agonists selective for these receptors (Extended Data Fig. 1h–k). We switched from using a broad-spectrum mGluR agonist, t-ACPD (Fig. 1), to an mGluR_3_-selective agonist, LY379268 (Extended Data Fig. 1h–k), to specifically examine the effect of this G_i_-GPCR activation on cAMP activity. In contrast to canonical G_i_-GPCR signalling, slow and sustained cAMP increases^24,25^ were observed with both agonists, with more cells responding to mGluR_3_ than to GABA_B_R activation (Extended Data Fig. 1j). When comparing astrocytic agonist-triggered Ca^2+^ and cAMP, we found significantly more dynamic Ca^2+^ activity compared to cAMP (Extended Data Fig. 1k). Although Ca^2+^ is not a canonical downstream signalling partner of G_i_-GPCRs, our results confirm previous findings that astrocytes do signal through mGluR_3_ and GABA_B_R to mobilize intracellular Ca^2+^ (refs. ^2,17,24,26^), potentially through phospholipase C signalling^27,28^ or by βγ-subunits directly binding to inositol trisphosphate receptors (IP_3_R)^29,30^. The relative lack of dynamism in cAMP compared to Ca^2+^ led us to focus only on Ca^2+^ as the second messenger more likely to exhibit NT-specific responses to spatiotemporally restricted—and more physiological—NT release.

## Single astrocytes respond to NT release

**Fig. 2:**
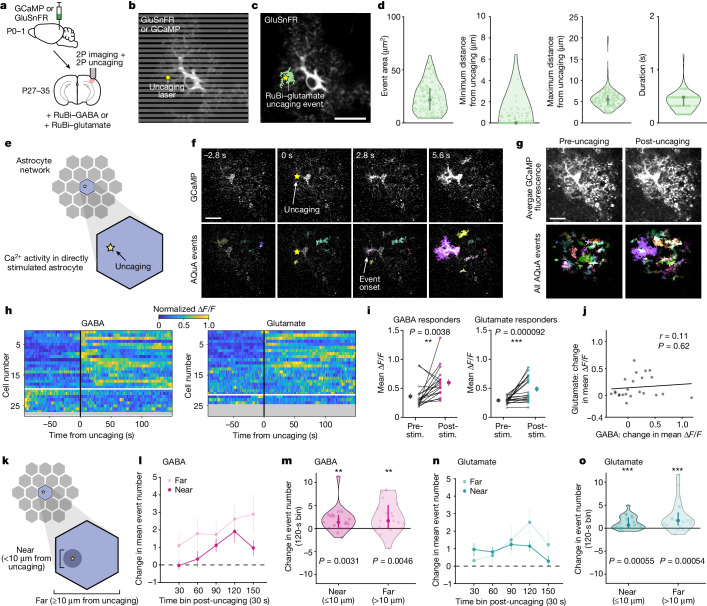
Subcellular, spatiotemporally restricted NT release increases Ca^2+^ activity within directly stimulated astrocytes. **a**, Experimental strategy for simultaneous ex vivo two-photon imaging of astrocyte Ca^2+^ (cyto-GCaMP6f) or extracellular glutamate (GluSnFR) and two-photon NT uncaging. **b**, Imaging and uncaging schematic. Grey lines: scanning laser. Yellow star: NT uncaging site. **c**, A representative GluSnFR event during RuBi–glutamate uncaging. **d**, GluSnFR event features post RuBi–glutamate uncaging. Data shown by individual glutamate events; median and 25th and 75th percentiles (*n* = 72 trials, 12 recordings, 4 slices, 2 mice). **e**, Schematic highlighting directly stimulated astrocyte. Analysis throughout figure includes only events from directly stimulated cells. **f**, Representative GCaMP6f fluorescence in astrocyte before and after RuBi–GABA uncaging. Yellow star: uncaging location and frame. **g**, Average GCaMP fluorescence 150–0 s pre- and 0–150 s post-stimulus from the astrocyte in **f**. **h**, Astrocyte Ca^2+^ stimulated by GABA or glutamate uncaging. Rows: average Δ*F*/*F* from AQuA-detected events per cell, normalized between 0 and 1 per cell. Cells sorted by onset time. Red line: NT uncaging. The white line separates responding (above) and non-responding cells (below). Responder cells: ≥1 post-stimulus frame with ∆*F*/*F* ≥ baseline mean + 3 s.d. Greyed-out rows: cells excluded because of baseline event frequency. **i**, Mean fluorescence pre- and post-stimulus from astrocytes responding to NT uncaging. Data are shown by cell and as mean ± s.e.m. **j**, Fluorescence change in stimulated astrocytes following NT uncaging. Pearson’s correlation shows no significant relationship between fluorescence change following GABA and glutamate uncaging (*P* = 0.62). **k**, A schematic of stimulated astrocyte compartments near and far from uncaging. Far compartment: uncaging outside NT spread radius (*d*, maximum distance from uncaging). **l**,**n**, Event frequency (events per 30 s) change near and far from GABA (**l**) and glutamate (**n**) uncaging within responding, stimulated cells. Data shown as mean ± s.e.m. **m**,**o**, Event frequency change during high activity period (90–120 s after uncaging, ‘120-s’ bin) from **l** and **n**, respectively. Data shown by cell; median and 25th and 75th percentiles. **h**–**j**,**l**–**o**, Pre-stimulus: 90–0 s before uncaging; post-stimulus: 0–150 s following uncaging. *n* = 27 (GABA) and 24 (glutamate) cells in **h**, 19/27 cells responded to GABA and 21/24 to glutamate in **i**,**l**–**o** and 24 paired cells in **j** all from 27 FOVs, 7 slices, 4 mice. **i**,**m**,**o**, Wilcoxon signed-rank test. All statistical tests are two-sided. Scale bars, 20 μm (**c**,**f**,**g**). NS: *P* ≥ 0.05; *P < 0.05; **P < 0.01; ***P < 0.001.

**Extended Data Fig. 2:**
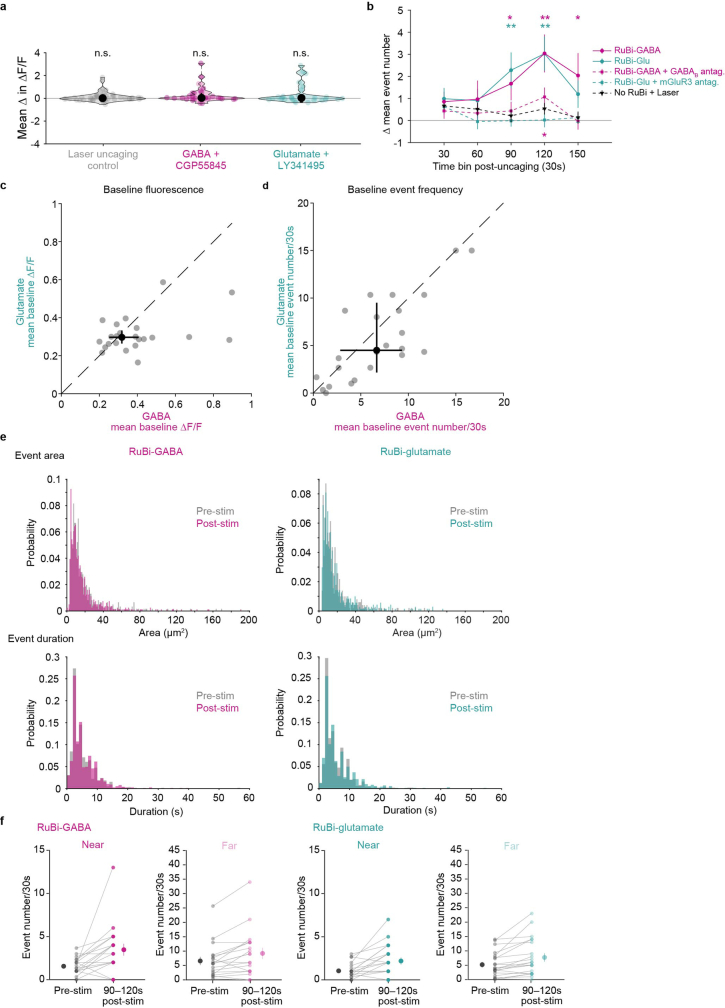
Characterization of, and controls for, increased Ca^2+^ activity in astrocytes directly stimulated by NT uncaging. **a**, Average change in ΔF/F with laser uncaging control (laser stimulation without RuBis, grey, n = 46 astrocytes, 9 slices, 3 mice) and with uncaging in the presence of antagonist (RuBi-GABA + GABA_B_R antagonist [magenta, n = 28 astrocytes, 8 slices, 5 mice] or RuBi-glutamate + mGluR_2/3_ antagonist [green, n = 28 astrocytes, 7 slices, 4 mice]). GABA_B_R antagonized using CGP55845, a potent and selective GABA_B_R antagonist, and mGluR_3_ antagonized using LY341495, a potent mGluR_2/3_ antagonist also known to antagonize other mGluR subtypes at higher concentrations^68^. Data shown by astrocyte, median, 25^th^ and 75^th^ percentile. Wilcoxon signed-rank test compares change from baseline. *p*-values corrected for multiple comparisons using Bonferroni-Holm correction with FWER ≤ 0.05. Laser uncaging control: *p* = 0.50, RuBi-GABA + CGP55845: *p* = 0.11 and RuBi-glutamate + LY341495: *p* = 0.41. **b**, Event frequency change after NT uncaging (GABA: solid magenta lines, n = 27 astrocytes, 7 slices, 4 mice; glutamate: solid green lines, n = 24 astrocytes, 7 slices, 4 mice), NT uncaging in the presence of antagonist (dotted magenta and green lines), and laser uncaging control (dotted black line). 90–0 s before and 0–150 s after uncaging used to calculate event number/30 s. Data shown by mean ± sem. Permutation test used to determine significance. *p*-values in Supplementary Table 5. **c**,**d**, Baseline fluorescence (**c**) and event frequency (**d**) prior to GABA and glutamate uncaging. 90–0 s before uncaging used to calculate mean ∆F/F (c) and mean number of events/30 s (d) per cell. Data shown by cell (grey dots, n = 24 astrocytes), median, and 25^th^ and 75^th^ percentile (black dots and crosshairs). Dashed line = unity line. Wilcoxon signed-rank tests show no significant difference between baseline features of directly stimulated astrocytes prior to GABA and glutamate uncaging (*p* = 0.089 [c, baseline fluorescence], 0.068 [d, baseline event frequency]). **e**, Distribution of event area and duration pre- and post-uncaging of RuBi-GABA (left) and RuBi-glutamate (right) from “responder” uncaging cells. Detected events 120 s pre- and post-uncaging are included from n = 19 astrocytes, 7 slice, 4 mice (GABA), 21 astrocytes, 7 slices, 4 mice (glutamate). Rank-sum test compares pre- and post-uncaging event features. Area: *p* = 0.58 (GABA) and 0.95 (glutamate). Duration: *p* = 0.083 (GABA) and 0.13 (glutamate). **f**, Event frequency in responding astrocytes directly stimulated with NT. Events from directly stimulated astrocytes separated into events near and far from GABA and glutamate uncaging. 90–0 s before used to calculate average event number/30 s (“pre-stim”). Data shown by cell (light dots and grey lines) and mean ± sem (dark dots and error bars). All statistical tests are two-sided.

To release NTs with spatiotemporal precision, we used two-photon photo-release (‘uncaging’) of caged NTs (Fig. 2a), as is commonly used to interrogate postsynaptic physiology through restricted activation area and duration^15,31,32^. To compare the effects of GABA and glutamate on the same astrocytes, we chose a class of caged compounds (with ruthenium bipyridine (RuBi) backbones), bound to either GABA^33^ or glutamate^34^, that can be two-photon-uncaged (800 nm) during simultaneous GCaMP Ca^2+^ imaging with a second two-photon laser (excitation 980 nm; Fig. 2b). With this strategy, the uncaging and imaging experimental paradigm is common to both GABA and glutamate conditions. To account for likely variability in the Ca^2+^ response to NTs across individual cells^35,36^, we imaged the same astrocytes while sequentially uncaging GABA and glutamate at the same subcellular location, separated by an inter-imaging interval of >20 min, including a washout period of >10 min. To account for any changes resulting from previous NT release, we alternated the order of GABA or glutamate uncaging between slices. To quantify the properties of NT release in this dual two-photon uncaging and imaging strategy, we first imaged an extracellular-facing glutamate sensor (GluSnFR^37^) while uncaging RuBi–glutamate (Fig. 2c). We confirmed that NT release was spatiotemporally confined to the intended location, over an area of about 25 μm^2^ and duration of 0.5–1 s (Fig. 2d). To ensure that the uncaging laser itself did not stimulate astrocytes, we also stimulated GCaMP-expressing astrocytes with the uncaging laser alone in the absence of RuBi–GABA or RuBi–glutamate, and did not observe a change in average Ca^2+^ fluorescence or event frequency (Extended Data Fig. 2a,b).

After validating the spatiotemporal precision of this approach, we next released NT during GCaMP imaging and analysed the Ca^2+^ activity within the directly stimulated astrocyte (Fig. 2e). We observed examples of Ca^2+^ increases within seconds, in close proximity to the uncaging site (Fig. 2f,g and Supplementary Videos 3 and 4). By plotting ∆*F*/*F* and sorting by latency-to-fluorescence increases, we saw most astrocytes increase Ca^2+^ activity following NT release (Fig. 2h, above the white line (70% and 88% of cells for GABA and glutamate, respectively), and Fig. 2i), but the area and duration of Ca^2+^ events were unchanged (Extended Data Fig. 2e). The activity increases often lasted for 2.5 min after NT release, the post-uncaging duration of the recording (Fig. 2h and Extended Data Fig. 2b), validating previous findings that NT-induced astrocyte Ca^2+^ activity can be long-lasting^2,6^. Comparing the same astrocyte’s response to both NTs, we found no significant relationship between the magnitude of its response to GABA versus glutamate (Fig. 2j), a controlled comparison given similar levels of activity within each cell before uncaging (Extended Data Fig. 2c,d). To confirm that the Ca^2+^ elevations were due to activation of astrocytic GPCRs, we next carried out NT uncaging in slices in which GABA_B_R or mGluR were inhibited pharmacologically, and found that Ca^2+^ increases were indeed blocked in these conditions (Extended Data Fig. 2a,b).

Astrocyte Ca^2+^ activity can be highly compartmentalized^3,4,35,36^, so we next tested whether observed changes in Ca^2+^ activity within the stimulated astrocyte were confined to subcellular regions directly exposed to initial NT release (<10 μm from uncaging; Fig. 2c,d). We found an increased frequency of Ca^2+^ events both near to (<10 μm) and far from (≥10 μm) the uncaging site (Fig. 2k–o and Extended Data Fig. 2f), with increases in both spatial domains peaking ≥1 min after uncaging for both NTs. These data demonstrate that spatiotemporally restricted NT release can drive Ca^2+^ activity in subcellular compartments extending beyond the stimulated region.

## Networks respond to subcellular NTs

**Fig. 3:**
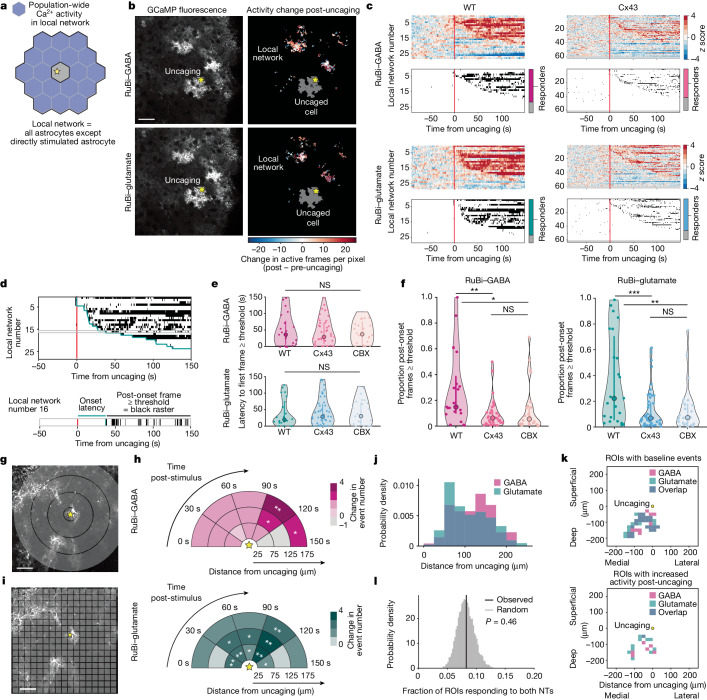
Subcellular release of NTs increases Ca^2+^ activity in the local astrocyte network through Cx43. **a**, Analysis throughout the figure is of population-wide Ca^2+^ activity from all astrocytes in the FOV not directly stimulated by uncaging. **b**, Representative astrocytic GCaMP6f fluorescence (left) and spatial heat maps of Ca^2+^ changes in local astrocyte network (right) following GABA and glutamate uncaging. Pre- and post-uncaging periods: 150 s before and after uncaging. Activity in the uncaged cell (dark grey) is excluded. **c**, Top: Ca^2+^ from all recorded local networks; rows show mean Δ*F*/*F* traces from AQuA-detected events per local network. Networks sorted by onset time. Red line: time of NT uncaging. Greyed-out rows: networks without detected events. Bottom: binarized raster plots show frames with *z* scores ≥ 3 (threshold). Stacked bar graphs: proportion of local networks exhibiting ≥1 post-stimulus frame ≥ threshold (responder). Two-sided Fisher’s exact test compares the proportion of responders across conditions: *P* = 0.62 (GABA WT versus *Cx43*-floxed), 0.78 (glutamate WT versus *Cx43*-floxed), 0.75 (GABA WT versus glutamate WT). **d**, Top: example binarized raster plot from **c**. Green line: response onset for each network (first post-stimulus frame ≥ threshold). Bottom: example local network, showing onset latency (green) as time between NT uncaging and response onset, and post-onset frames ≥ threshold (black ticks). **e**, Onset latency. One-way analysis of variance compares onset latency across conditions. *P* = 0.82 (GABA), 0.89 (glutamate). **f**, Persistence of network-level responses (proportion of post-onset frames ≥ threshold). One-way analysis of variance followed by Tukey–Kramer test for each NT. GABA: *P* = 0.0010 (WT versus *Cx43*-floxed), 0.025 (WT versus CBX), 0.72 (*Cx43*-floxed versus CBX). Glutamate: *P* = 0.00034 (WT versus *Cx43*-floxed), 0.0032 (WT versus CBX), 0.98 (*Cx43*-floxed versus CBX). **g**, Sholl-like analysis. Grey circles: 50-µm bands. Yellow star: NT uncaging site. **h**, Ca^2+^ event frequency change in local network after NT uncaging. Permutation test to determine significance. Two-sided *P* values in Supplementary Table 6. **i**, Grid-based ROI (20 µm^2^). **j**, Distances from uncaging site to centre of ROIs active post-uncaging. Active ROIs: ROIs with ≥50% event frequency increase post-uncaging. *n* = 195 active ROIs (GABA), 171 active ROIs (glutamate) from 27 paired FOVs. **k**, Example FOV of ROIs with baseline events (left) and active ROIs post-uncaging (right). Yellow dot: NT uncaging site. **l**, Fraction of ROIs active (responding) following both GABA and glutamate uncaging, among all active ROIs for uncaging of either NT (black vertical line; 8.27 ± 1.34%, mean ± sem; *n* = 27 paired FOVs). One-sided *P* value compares observed overlap fraction (Jaccard index) to surrogate data (grey distribution). **e**,**f**, Data shown by responding network; median, and 25th and 75th percentiles. *n* = 28 networks, 7 slices, 4 mice (WT) in **c**,**h**, 63 networks, 16 slices, 8 mice (*Cx43*-floxed) in **c**, 21 networks responding to GABA and 23 to glutamate from 7 slices, 4 mice (WT); 42 networks responding to GABA and 47 to glutamate in 16 slices, 8 mice (*Cx43*-floxed); 24 networks responding to GABA and 24 to glutamate in 8 slices, 4 mice (CBX) in **e**,**f**. Scale bars, 50 µm (**b**,**g**,**i**). NS: *P* ≥ 0.05; *P < 0.05; **P < 0.01; ***P < 0.001.

**Extended Data Fig. 3:**
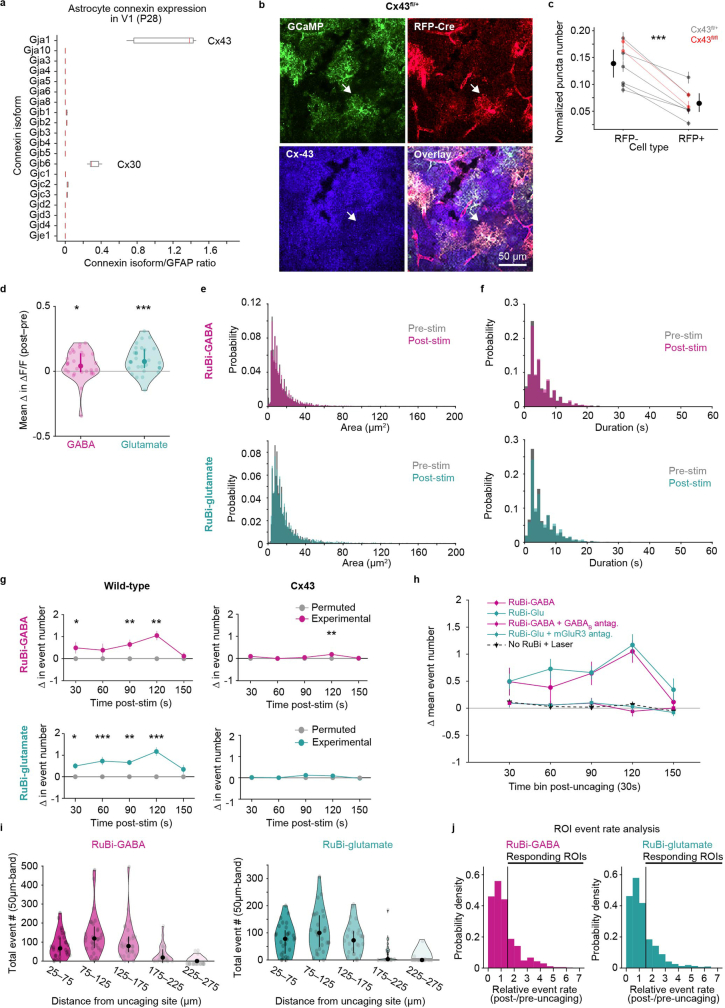
Confirmation of Cx43 knockdown and network-level controls after NT uncaging. **a**, Ribosomal-mRNA expression in visual cortex astrocytes of P28 mice (n = 5 biological replicates) from the Farhy-Tselnicker et al. publicly available dataset (NCBI Gene Expression Omnibus, GSE161398). Visual cortex astrocytes preferentially express Cx43 (Gja1) over other connexins, including Cx30 (Gjb6). Similar expression levels are found in the Srinivasan et al. dataset available at http://astrocyternaseq.org. Ratio of FPKM for the gene of interest / FPKM for GFAP were calculated to normalize for potential differences in the sequencing depth of replicates. Center line: median; box limits: 25^th^ and 75^th^ percentiles; whiskers: minimum and maximum values. **b**, Representative micrographs of immunohistochemistry in a Cx43^fl/+^ slice demonstrating reduced numbers of Cx43 puncta in Cre^+^ astrocytes. White arrow points to individual cell expressing GCaMP (green) and RFP-Cre (red), with reduced Cx43 (blue). **c**, Average Cx43 puncta/astrocyte in RFP-Cre^−^ and RFP-Cre^+^ astrocytes; puncta counts are normalized by area of each astrocyte. Data are shown by mouse averages (light dots, error bars and connecting lines, grey = Cx43^fl/+^ and red = Cx43^fl/fl^ mice) and mean ± sem (dark dots and error bars). Cx43 puncta counts were similar for Cx43^fl/+^ and Cx43^fl/fl^ mice; data from both genotypes were pooled together for all analyses and referred to as Cx43^floxed^ (n = 8 mice). Paired two-sided t-test compares average Cx43 puncta counts in RFP-Cre^−^ and RFP-Cre^+^ astrocytes. *p* = 0.00013. **d**, Average change in ∆F/F in WT astrocyte networks after RuBi-GABA (magenta) and RuBi-glutamate (green) uncaging. Data shown by trial/FOV, median and 25^th^ and 75^th^ percentile. Wilcoxon signed-rank test compares change from baseline. *p* = 0.016 (GABA) and 0.00032 (glutamate). **e**,**f**, Distribution of event area and duration pre- and post-uncaging of RuBi-GABA (top) and RuBi-glutamate (bottom). Detected events 120 s pre- and post-uncaging are included. Rank-sum test compares pre- and post-uncaging event features. Area: *p* = 0.025 (GABA) and 0.0050 (glutamate). Duration: *p* = 0.063 (GABA) and 0.0000045 (glutamate). **g**,**h**, Event frequency change in neighboring astrocytes after GABA (g, top) and glutamate (g, bottom) uncaging in WT and Cx43^floxed^ slices. WT data from g replotted in h (circular markers) with laser uncaging control (laser stimulation without RuBis, dotted black line and triangular markers) and with uncaging in the presence of antagonist (RuBi-GABA + GABA_B_R antagonist [magenta line and square markers] or RuBi-glutamate + mGluR_2/3_ antagonist [green line and square markers]). 90–0 s before and 0–150 s after uncaging used to calculate event number/30 s in neighboring astrocytes with ≥ 1 AQuA-detected event. Data shown by mean ± sem. Permutation test used to determine significance. *p*-values in Supplementary Table 7. **i**, Total number of AQuA-detected events in 50 µm bands radiating out from the uncaging site. All events 90 s before and 150 s after NT uncaging are included. Data shown by trial/FOV, median and 25^th^ and 75^th^ percentile. **j**, Distribution of relative event rates from 20×20 µm ROIs following uncaging of RuBi-GABA (left) and RuBi-glutamate (right). Validation for threshold used to define ROIs with increased activity post-uncaging; chosen threshold: ≥ 50% event frequency increase post-uncaging. *n* = 28 networks, 7 slices, 4 mice (WT) in **d**–**i**, 61 networks, 16 slices, 8 mice (Cx43^floxed^) in **g**,**h**, 48 networks, 9 slices, 3 mice (laser uncaging control), 32 networks, 8 slices, 5 mice (RuBiGABA + GABA_B_R antagonist), 28 networks, 7 slices, 4 mice (RuBi-glutamate + mGluR_2/3_ antagonist) in **h**. All statistical tests are two-sided.

To examine whether activity changes extended beyond single cells, we next investigated population-wide Ca^2+^ activity in neighbouring astrocytes within the gap junctionally coupled local network (Fig. 3a). Within the 300 × 300 μm imaging field of view (FOV), the astrocyte over which NT was uncaged was approximately centred. Neighbouring astrocytes (*n* = 10.3 ± 3.85; mean ± s.d.) with GCaMP6f activity were imaged and distinguished from the uncaged cell by delineating cell maps. The active neighbouring astrocytes within a given FOV define a ‘local network’ (Fig. 3a,b). We observed general Ca^2+^ increases within the local network of astrocytes after uncaging (Fig. 3b, Extended Data Fig. 3d–f and Supplementary Videos 5 and 6). Although we saw heterogeneity in the timing and magnitude of local network responses to subcellular NT release in the uncaged cell, most imaged networks responded with population-wide fluorescence increases (Fig. 3c, left).

To investigate whether gap junctional coupling mediates these non-cell-autonomous Ca^2+^ activity changes after a single point of network stimulation, we genetically or pharmacologically inhibited gap junctions and measured population-wide network Ca^2+^ responses (Fig. 3c–f). Genetically, we focused on the predominant connexin protein (Cx43) expressed in cortical astrocytes^18,19,38^ (Extended Data Fig. 3a), and decreased the Cx43 expression level mosaically by injecting the astrocyte-specific Cre virus AAV5-GFAP(0.7)-RFP-T2A-iCre^39^ (and AAV5-GfaABC1D-GCaMP6f-SV40 to express GCaMP) into *Cx43*^*fl/+*^ and *Cx43*^*fl/fl*^ mice. Decreases in the level of Cx43 protein in Cre^+^ cells were confirmed through immunohistochemistry (Extended Data Fig. 3b,c and Supplementary Video 7). After targeting Cre^+^ astrocytes for RuBi–GABA and RuBi–glutamate uncaging, population-wide network activity changes were attenuated compared to those observed in wild-type (WT) slices (Fig. 3c, right). Although population-wide fluorescence did rise above threshold in some post-stimulus frames in *Cx43*-floxed and carbenoxolone (CBX, broad pharmacological gap junctional blocker)-treated networks with similar onset latencies to WT networks (Fig. 3d,e), the proportion of time that population-wide activity remained in an elevated state was significantly reduced in networks with gap junctional inhibition (Fig. 3c,f). Additionally, *Cx43*-floxed networks showed no significant increase in average event frequency, similar to the laser-uncaging controls and receptor-activation controls in slices in which GABA_B_R or mGluR was inhibited pharmacologically during uncaging (Extended Data Fig. 3g,h). These results indicate that astrocytic Cx43-based signalling may play a role in network-level Ca^2+^ increases following NT release elsewhere in the local network. Further, these observations hint that reduced Ca^2+^ signalling in uncoupled astrocyte networks may underlie altered neuronal network activity and deficits in sensory-related behaviours observed in connexin-deficient mice^40,41^.

We next examined how far NT-induced local network activity extended from the uncaged cell. Using a Sholl-like analysis (Fig. 3g), we observed event frequency increases as far away as 125–175 µm from uncaging of both NTs (Fig. 3h), to the edge of the FOV (Extended Data Fig. 3i). To compare the spatial distribution of these network-level responses between GABA and glutamate, we then analysed event activity within 20 × 20 µm ROIs in a grid over the FOV (Fig. 3i–k). As in the Sholl-like analysis (Fig. 3h), ROIs with uncaging-driven activity were distributed both near and far from the uncaging site (GABA: 119.9 ± 46.1 μm; glutamate: 109.3 ± 49.4 μm (mean ± s.d.); Fig. 3j). Further, whereas baseline activity encompasses contiguous, overlapping portions of the astrocyte network (Fig. 3k, top), ROIs exhibiting an event increase after NT uncaging were sparse (Extended Data Fig. 3j) and, critically, exhibit no significant overlap between responses to GABA and glutamate (Fig. 3k, bottom and Fig. 3l), suggesting that GABA and glutamate do not primarily activate the same regions of the astrocyte network. Together these data show that focal release of NT at a single cortical astrocyte leads to spatially distributed changes in Ca^2+^ activity across an astrocyte network.

## Propagation separates network responses

**Fig. 4:**
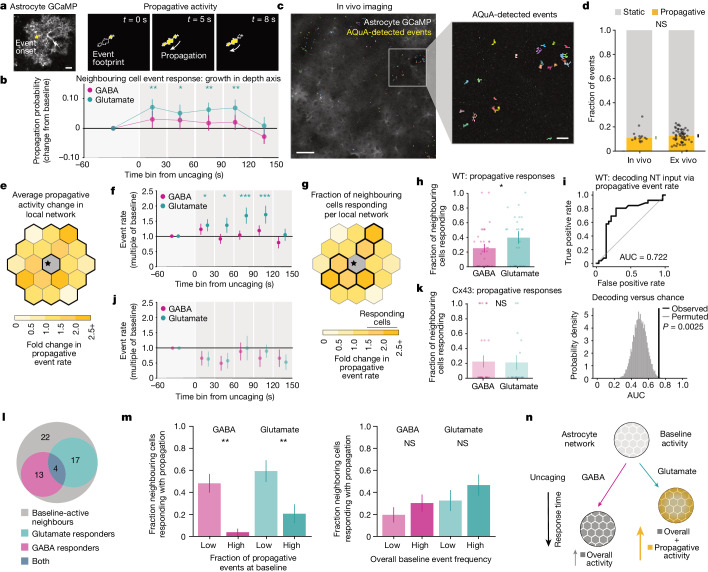
Propagative activity distinguishes astrocyte network responses to GABA and glutamate. **a**, Astrocytic GCaMP6f fluorescence with initial territory (left) and subsequent trajectory (right) of a propagating event in yellow. Outline: total event territory. **b**, Probability change of Ca^2+^ event growing in the depth axis (relative to pia) among all events from neighbouring cells after NT uncaging. Data shown as overall probability ± standard error (*n* = 142 cells, 28 FOV (GABA), 120 cells, 27 FOV (glutamate)). Two-sided *P* and *q* values by permutation testing (Supplementary Table 8). **c**, Two-photon image of in vivo astrocyte GCaMP6f in V1. Overlay: Ca^2+^ events from 90-s stationary period. **d**, Propagative event fraction in V1 during stationary wakefulness in vivo and baseline in acute V1 slices. Data shown by recording; median ± standard error by bootstrapping (*n* = 15 recordings, 5 mice (in vivo), 55 recordings, 4 mice (ex vivo)). Two-sided rank-sum test (*P* = 0.57). **e**,**f**,**j**, Schematic (**e**) and quantification of fold change in propagative event rate across neighbouring cells per FOV after NT uncaging in WT (**f**) or *Cx43*-floxed (**j**) slices. Data shown as median across FOVs ± standard error. One-sided *P* and *q* values by permutation testing (see Supplementary Tables 9 and 10). As in Fig. 3, directly stimulated astrocyte excluded from all figure analyses. **g**,**h**,**k**, Schematic (**g**) and quantification of fraction of neighboring cells per FOV with ≥50% propagative event rate increase (‘responding’) after NT uncaging in WT (**h**) or *Cx43*-floxed (**k**) slices. Data shown by FOV; mean ± sem (see Supplementary Table 9). Two-sided *P* values by permutation testing, *P* = 0.046 (WT), 1.0 (*Cx43*-floxed). **i**, Top: receiver operating characteristic curve decoding NT identity by thresholding relative propagative event rate change across all neighbouring cells per FOV. Bottom: observed area under the receiver operating characteristic curve (AUC) = 0.72 ± 0.077 (value ± bootstrapped standard error), compared to permuted distribution through permuting NT labels (*P* = 0.0025, *n* = 55 FOVs, one-sided). **l**, Neighbouring cell numbers responding to one or both NTs with propagative activity increases, among cells with baseline propagative activity (*n* = 56 cells, 24 paired recordings, 7 slices, 4 mice). Permutation testing measures of correlation (two-sided Spearman *ρ*, *P* = 0.24) or overlap (one-sided Jaccard index, *P* = 0.96) between GABA and glutamate responses. **m**, Fraction of neighbouring cells responding with propagative increases after NT uncaging, cells equally divided by low and high baseline activity features (split at 50th percentile). Baseline activity features: fraction of propagative events (left), overall event rate (right, see Extended Data Fig. 5e). Data shown as mean ± s.e.m. (see Supplementary Table 11). Response fractions for cells with ‘low’ and ‘high’ baseline fractions were compared by permuting cells’ baseline propagation fractions for GABA (*P* = 1.0 × 10^–4^) and glutamate (*P* = 0.0012); responses for cells with ‘low’ and ‘high’ overall baseline event rates were compared similarly (GABA: *P* = 0.25; glutamate: *P* = 0.25). **n**, Integrated model of astrocyte network responses. Astrocyte networks increase general Ca^2+^ with both NTs, and propagative activity specifically with glutamate. Network responses to glutamate are faster than those to GABA. **b**,**f**,**h**,**j**,**k**,**m**, error bars by hierarchical bootstrapping. **b**,**f**, *q < 0.05, **q < 0.01, ***q < 0.001, **h**,**m**, *P < 0.05, **P < 0.01. Scale bars, 10 μm (**a**,**c** (right)) and 50 μm (**c** (left)).

**Extended Data Fig. 4:**
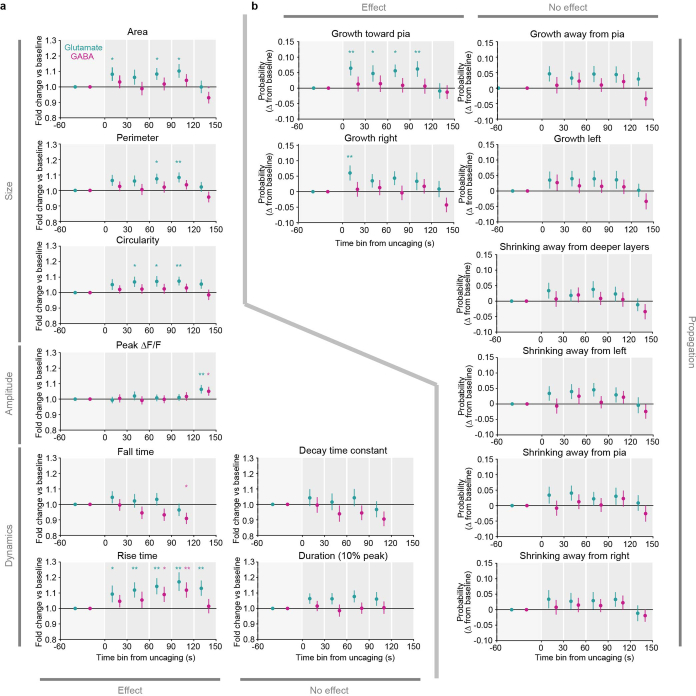
Change in individual astrocyte Ca^2+^ event features post NT-uncaging. **a**, Fold change in indicated Ca^2+^ event features among all events from all neighboring cells after GABA or glutamate uncaging, relative to 60–0 s pre-uncaging. Data shown as overall mean ± sem determined from hierarchical bootstrapping (see Methods). Two-sided *p*- and *q*-values for changes versus baseline were obtained by circularly shifting each cell’s events in time (see Methods; Supplementary Table 12). **b**, Change in the probability of a Ca^2+^ event growing or shrinking in the indicated direction among all events from neighboring cells after GABA or glutamate uncaging, relative to 60–0 s pre-uncaging. Data shown as overall probability ± standard error determined from hierarchical bootstrapping (see Methods). Two-sided *p*- and *q*-values for changes versus baseline were obtained by circularly shifting each cell’s events in time (see Methods; Supplementary Table 13). *n* = 142 cells in 28 FOV [GABA], 120 cells in 27 FOV [glutamate] in **a**,**b**.

**Extended Data Fig. 5:**
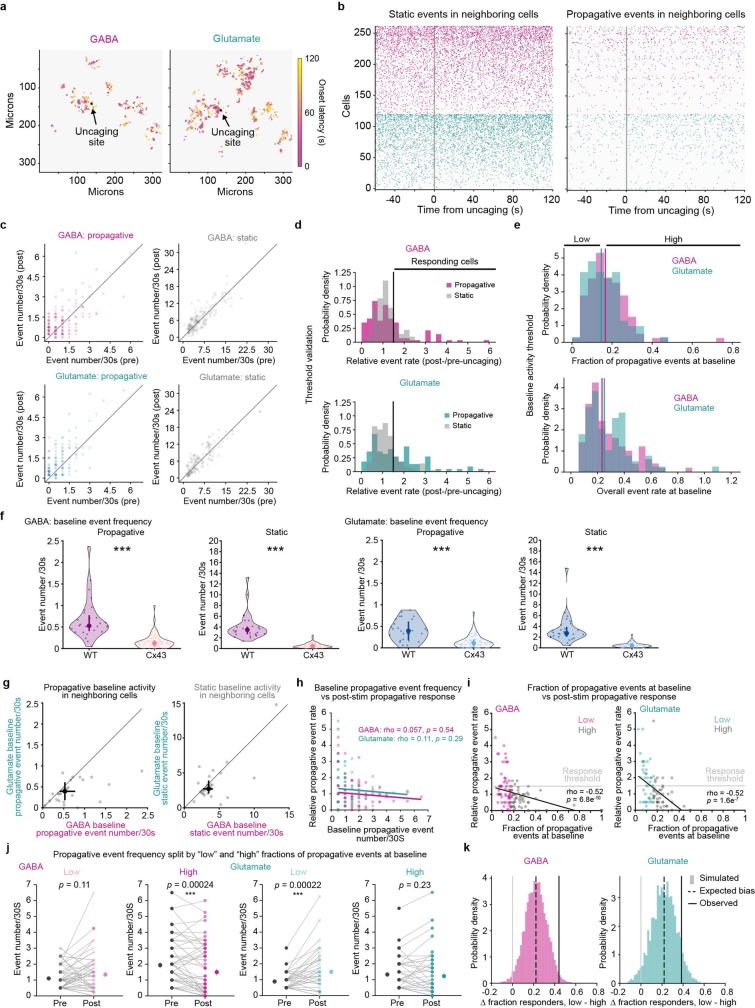
Validating changes in propagative event activity following NT-uncaging. **a**, Representative spatial maps of Ca^2+^ events in the same astrocyte network 0–120 s after GABA (left) or glutamate (right) uncaging. Events are color-coded by onset time. Black dot = NT uncaging site. Events from all time-points are distributed throughout the imaging field, with no visible wavefront of activity traveling across the imaging field or emanating from the uncaging site. Note that all panels except for (f) are data from WT slices. **b**, Raster plots of Ca^2+^ event onsets for static (left) or propagative (right) events before and after GABA (magenta) or glutamate (green) uncaging. Raster plots show all neighboring cells (astrocytes not directly stimulated by NT-uncaging) from all FOVs, with each row showing events from an individual astrocyte. Within each NT and event type, cells were sorted by the overall rate of static events from 0–120 s post-uncaging (*i.e*., the same sorting was used for the left and right raster plots). Grey line = NT uncaging start. **c**, Scatter plots of event rates (event number/30 s) within neighboring cells during the period 60–0 s pre-uncaging (*x*-axis) versus 0–120 s post-uncaging (*y*-axis). Rates of propagative (left) and static (right) events are shown for recordings of GABA (top) and glutamate (bottom) uncaging. Dots are individual neighboring cells; darker dots indicate multiple overlapping cells. **d**, Distribution of post-/pre-uncaging ratio of static (grey) or propagative (color) event rates among neighboring cells with any baseline events of the corresponding type, after GABA (magenta, top) or glutamate (green, bottom) uncaging. Ratios computed per-cell as the rate from 0–120 s post-uncaging divided by the rate from 60–0 s pre-uncaging. Vertical black lines indicate the threshold used to determine “responding” cells in Fig. 4h, l, m and Extended Data Fig. 6e (*i.e*., ≥ 1.5-fold). **e**, Top: Distribution of the fraction of events during the baseline window (60–0 s pre-uncaging) that were propagative in each neighboring cell before GABA (magenta) or glutamate (green) uncaging, among those cells that had any baseline propagative activity. Vertical magenta and green lines indicate the thresholds (50^th^ percentile) for recordings of GABA and glutamate uncaging, respectively, used in Fig. 4m left to delineate “Low” and “High” fraction of propagative events at baseline among neighboring cells. Bottom: Distribution of the overall event rate during the baseline window of 60–0 s pre– GABA (magenta) or glutamate (green) uncaging, in each neighboring cell that had baseline propagative activity. Vertical magenta and green lines indicate the thresholds (50^th^ percentile) for recordings of GABA and glutamate uncaging, respectively, used in Fig. 4m right to delineate cells with “Low” and “High” overall event rates at baseline. **f**, Baseline propagative (left) and static (right) event frequencies of astrocytes in WT or Cx43^floxed^ slices. Baseline period: 90–0 s prior to uncaging. Individual data points show average event rate from active neighboring astrocytes ( ≥ 1 AQuA-detected event during recording) for each FOV. Data shown by FOV (WT: *n* = 28 FOV for GABA and glutamate, 7 slices, 4 mice; Cx43^floxed^: *n* = 63 FOV for GABA and 61 FOV for glutamate, 16 slices, 8 mice), median, 25^th^ and 75^th^ percentile. Wilcoxon rank sum test compares WT and Cx43^floxed^ baseline event frequencies (GABA: *p* = 1.6e-10 [propagative], 7.7e-14 [static]; glutamate: *p* = 9.0e-7 [propagative], 1.1e-12 [static]). **g**, Baseline propagative (left) and static (right) event frequencies in WT networks prior to GABA and glutamate uncaging. 90–0 s before uncaging used to calculate mean number of events/30 s. Event rate per FOV calculated by averaging the event rates of active astrocytes in the FOV ( ≥ 1 AQuA-detected event during the recording), excluding the uncaging astrocyte. Data shown by FOV (grey dots, n = 28), median, 25^th^ and 75^th^ percentile (black dot and crosshairs). Wilcoxon signed-rank test compares baseline event frequencies prior to GABA and glutamate uncaging (*p* = 0.00022 [propagative] and 0.052 [static]). **h**, Spearman correlation between baseline propagative event rate and relative post-stim propagative event rate for neighboring cells in GABA (magenta) and glutamate (turquoise) recordings. Data shown by individual neighboring astrocyte (for h–i, n = 121 cells [GABA], 91 cells [glutamate] with ≥ 1 baseline propagative event); solid lines: linear regression lines. For h–i, 60–0 s before uncaging used for baseline window and relative post-stim propagative rate calculated as in d. **i**, Spearman correlation between fraction of propagative events at baseline and relative post-stim propagative event rate for neighboring cells in GABA (left) and glutamate (right) recordings. Data shown by individual neighboring astrocyte color-coded by baseline activity composition category (“low” in magenta or turquoise, “high” in grey). Light grey horizontal line = response threshold (responders ≥ 1.5-fold increase in propagative activity from baseline). Note a majority of astrocytes responding to either NT (at or above the response threshold line) display a low fraction of propagative events at baseline. **j**, Propagative event frequency pre- and post-uncaging for neighboring cells with “low” and “high” fractions of propagative events at baseline (as for Fig. 4m, left). 60–0 s before (“Pre”) and 0–120 s after (“Post”) used to calculate average event number/30 s. Data shown by cell (light dots and grey lines; n = 61 cells [GABA “low”], 60 cells [GABA “high”], 46 cells [glutamate “low”], 45 cells [glutamate “high”]) and mean ± sem (dark dots and error bars). Wilcoxon signed-rank test compare pre-and post-stim frequencies for each category. **k**, Contextualization of observed differences in response fraction among neighbor cells, between low and high fraction of propagative events at baseline, compared with surrogate distribution from structured point process simulations. The fraction of simulations with low–high differences larger than the observed difference is indicated. Note limitations on direct comparison between observed values and simulation results (Methods). All statistical tests are two-sided.

As astrocyte Ca^2+^ events are highly heterogeneous^8^, we next carried out an unbiased analysis screen for changes in 16 event characteristics from neighbouring cells (Extended Data Fig. 4a,b). The most robust and consistent NT-specific changes in neighbouring cells were in events exhibiting propagation, with directionality towards the pia (Fig. 4a,b, Extended Data Fig. 4b and Supplementary Videos 3–6), which echoed a change we observed above in populations of astrocytes following more widespread NT exposure (Fig. 1h). These discrete propagative events occurred within individual cells (Fig. 4a), and we did not observe coordinated activity propagating across populations of astrocytes with a visible wavefront (Extended Data Fig. 5a). As propagative events constituted a small subset of spontaneous ex vivo astrocyte Ca^2+^ activity (Extended Data Fig. 5b), we wanted to ensure that they reflected in vivo Ca^2+^ activity. To test this, we recorded spontaneous astrocyte Ca^2+^ activity from the same cortical region (V1) in head-fixed mice^5,8^ (Fig. 4c). We focused on spontaneous astrocyte Ca^2+^ activity when the mouse was stationary, to eliminate locomotion-triggered Ca^2+^ bursts^8–10,42^. We found a similar fraction of propagative events ex vivo and in vivo (Fig. 4d), suggesting that this small subset of Ca^2+^ activity could constitute a physiologically relevant population.

**Extended Data Fig. 6:**
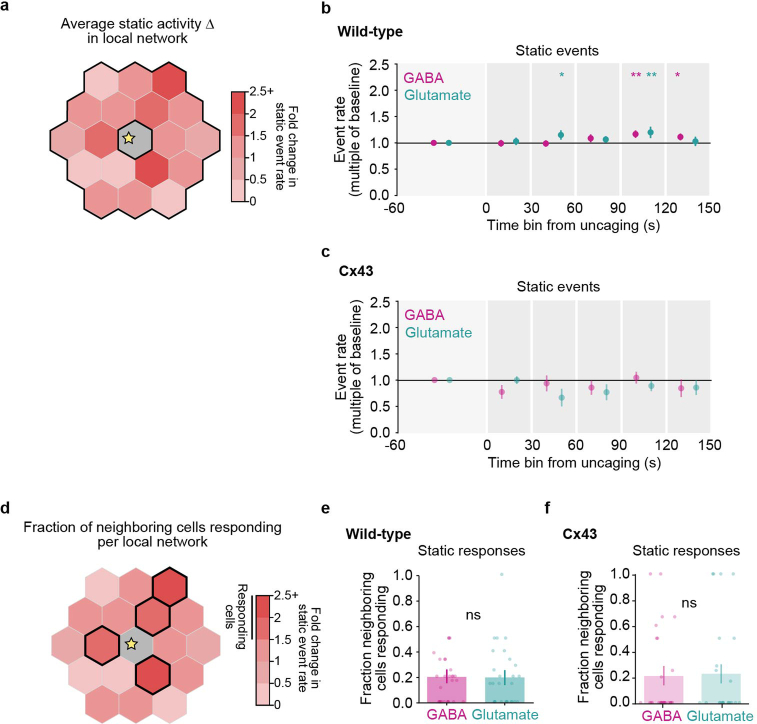
Static activity changes in the local astrocyte network are similar in response to GABA and glutamate. **a**, Analysis schematic illustrating average static activity change across all neighboring cells in the local network, as reported in b and c. Heterogeneous responses of individual neighboring cells are averaged in b and c. **b**–**c**, Fold-change in rate of static Ca^2+^ events among neighboring cells after GABA or glutamate uncaging in acute slices from WT mice (**b**) or Cx43^floxed^ mice (**c**), relative to 60–0 s pre-uncaging. Data shown as median across FOVs ± standard error via hierarchical bootstrapping (Methods; *n* in Supplementary Table 9). One-sided *p*- and *q*-values were obtained via circular permutation testing (Methods; Supplementary Table 10); *: *q* < 0.05, **: *q* < 0.01. **d**, Analysis schematic illustrating the fraction of neighboring cells per FOV that respond to NT with increases in static activity, as reported in e and f. **e**–**f**, Fraction of neighboring cells per FOV with ≥ 50% increase in static Ca^2+^ events (*responding*) after GABA or glutamate uncaging in WT (**e**) or Cx43^floxed^ slices (**f**). Data shown as mean ± sem via hierarchical bootstrapping; dots denote individual FOVs (see Methods; *n* in Supplementary Table 9). Permutation testing was used to compare fraction of cells responding to GABA and glutamate in WT slices (two-sided *p* = 1.0) and Cx43^floxed^ slices (two-sided *p* = 1.0).

Ex vivo, propagative event frequency specifically increases after glutamate uncaging, in all 30-s time-bins 0–120 s post-uncaging across neighbouring cells (Fig. 4e,f and Extended Data Fig. 5c), whereas no changes were observed across neighbouring cells after GABA uncaging in these same slices. Indeed, local network responses to glutamate and GABA uncaging can be distinguished by the fraction of cells with propagative event frequency changes (Fig. 4g), in which a higher fraction of astrocytes in each local network respond with increased propagative activity to glutamate (about 40%) compared to GABA (about 25%; Fig. 4h and Extended Data Fig. 5d). Further, the NT input received can be accurately decoded using the relative change in propagative event rate per FOV (Fig. 4i). By contrast, a similar fraction of local network astrocytes responds to GABA and glutamate with increased static event frequency (Extended Data Fig. 6). Astrocytes in the local network exhibited significantly higher baseline propagative activity and similar levels of static activity before uncaging GABA compared to glutamate (Extended Data Fig. 5g). Although this could influence results, these baseline differences do not account for the differential network responses to the two NTs, because the baseline propagative rate is not correlated with the relative post-uncaging propagative event rate (Extended Data Fig. 5h). These results indicate that glutamate and GABA are differentially encoded at the network level by engaging local network astrocytes to differing degrees through Ca^2+^ events that propagate within individual cells (Fig. 4n). As there are few propagative events at baseline, a small increase in propagative events following uncaging is a large relative activity increase, and may constitute a salient signal with a high signal-to-noise ratio. This increase in glutamate-driven propagative responses is not observed when uncaging NTs in astrocyte networks with a decreased level of Cx43 expression (Fig. 4j,k), which show significantly lower baseline activity compared to WT networks (Extended Data Fig. 5f). These data suggest that gap junction coupling may contribute to this NT-specific increase in propagative activity.

**Extended Data Fig. 7:**
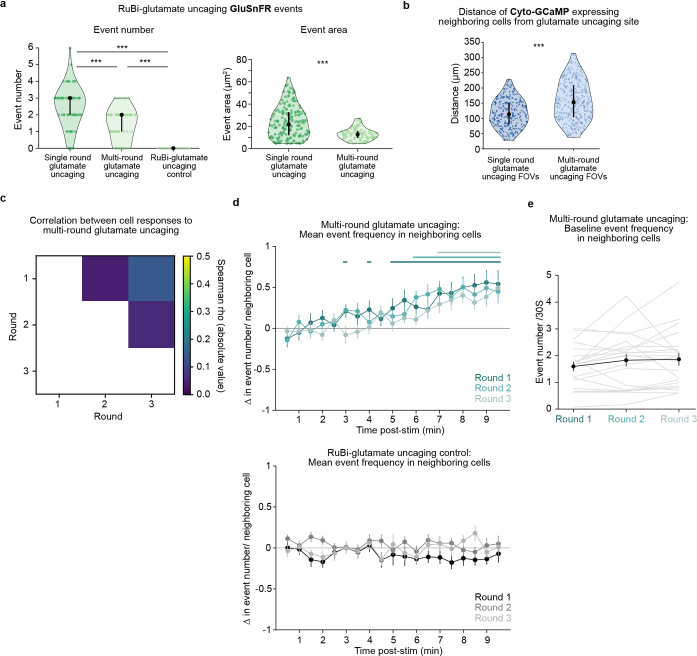
Individual neighboring astrocytes exhibit variable Ca^2+^ responses across multiple rounds of glutamate uncaging in WT networks. **a**, GluSnFR event features after RuBi-glutamate uncaging for three types of uncaging datasets. For number of events/uncaging site (left), data shown by uncaging trial, median, 25^th^ and 75^th^ percentile. For GluSnFR event area (right), data shown by GluSnFR event, median, 25^th^ and 75^th^ percentile (single round glutamate uncaging: *n* = 72 trials, 12 recordings, 4 slices, 2 mice; multi-round glutamate uncaging: *n* = 66 trials, 11 recordings, 2 slices, 1 mouse; RuBi-glutamate uncaging control: *n* = 66 trials, 11 recordings, 2 slices, 1 mouse). For number of events, one-way ANOVA followed by Tukey-Kramer Test determine significant pairwise comparisons between laser stimulation conditions. *p* = 9.7e-10 (single round glutamate uncaging v multi-round glutamate uncaging), 9.6e-10 (single round glutamate uncaging v RuBi-glutamate uncaging control) and 9.6e-10 (multi-round glutamate uncaging vs RuBi-glutamate uncaging control). For event area, rank sum test compares single round glutamate uncaging vs. multi-round glutamate uncaging, *p* = 3.6e-10. All datasets were collected in the presence of RuBi-glutamate. For single round and multi-round glutamate uncaging, the uncaging laser power was set to 70 A.U. (~8 mW at the sample). Laser re-alignment between these datasets leads to a small difference in amount of glutamate uncaged with laser stimulation (see event area on right). For RuBi-glutamate uncaging controls, the uncaging laser power was set to 25 A.U. (~2 mW at the sample), a stimulation that did not lead to detectable glutamate uncaging (see event number on left). **b**, Distance of Cyto-GCaMP-expressing neighboring astrocytes from the glutamate uncaging site. Distance measured from the centroid of each neighboring astrocyte to the centroid of the uncaging site. Data shown by active astrocyte (≥1 AQuA-detected event 0–300 s from recording onset), median, 25^th^ and 75^th^ percentile (single round glutamate uncaging: *n* = 28 FOV, 7 slices, 4 mice; multi-round glutamate uncaging: *n* = 23 FOV, 9 slices, 5 mice). Rank sum test compares datasets; *p* = 3.4e-15. **c**, Correlation between the propagative Ca^2+^ responses of individual neighboring cells to multiple rounds of glutamate uncaging. Individual cells’ binary responses to glutamate uncaging are not significantly correlated across rounds (Spearman rho = 0.040, *p* = 1.0, n = 32 cells, 15 recordings, 8 slices, 5 mice [round 1 vs 2]; Spearman rho = 0.14, *p* = 0.70, n = 30 cells, 16 recordings, 7 slices, 5 mice [round 1 vs 3]; Spearman rho = 0.059, *p* = 0.74, n = 38 cells, 17 recordings, 8 slices, 5 mice [round 2 vs 3]), showing that the response of an individual cell is variable from round to round. In each round, activity was recorded 150–0 s before and 0–600 s following uncaging, with glutamate uncaged over an area of ~12 µm^2^ (as in a, right “Multi-round glutamate uncaging”). Rounds of imaging/uncaging for each FOV were separated by ≥ 25 min. Cells included in analysis for each round had ≥ 1 propagative event during 60–0 s before uncaging. Responding cells exhibited ≥ 50% increase in propagative event frequency 300–420 s following uncaging, a time window in which activity began to increase across rounds, compared to 60–0 s before uncaging. **d**, Event frequency change in neighboring astrocytes across three rounds of glutamate uncaging (top) and RuBi-glutamate uncaging controls (bottom). 90–0 s before and 0–570 s after uncaging used to calculate mean event number/30 s in active astrocytes (astrocytes in the local network with ≥1 AQuA-detected event during recording, excluding the stimulated cell). Data shown by mean ± sem (multi-round glutamate uncaging: n = 23 FOV for Round 1 and 3, 21 FOV for Round 2, 9 slices, 5 mice; RuBi-glutamate uncaging control: n = 20 FOV, 8 slices, 5 mice). Permutation test used to determine significance. *p*-values in Supplementary Table 14. The responses in multi-round glutamate uncaging are delayed compared to the single round glutamate uncaging dataset (Extended Data Fig. 3g). Two factors may account for this delay. First, less NT is released in the multi-round glutamate uncaging dataset (a). Second, the distance of astrocytes in the local network from the uncaging site is greater in the multi-round uncaging dataset compared to the single round uncaging dataset (b). **e**, Baseline event frequencies for neighboring astrocytes across three rounds of glutamate uncaging. 90–0 s before uncaging used to calculate mean event number/30 s/active astrocytes in each FOV. Data shown by FOV (light grey lines, n = 21 FOV, 9 slices, 5 mice) and mean ± sem (black dots and error bars). Repeated measures ANOVA compares baseline frequencies across rounds (F(2,40) = 1.51, *p* = 0.23). All statistical tests are two-sided.

Similar to the finding that network-level responses to glutamate and GABA were spatially non-overlapping (Fig. 3k,l), our observations show that, of astrocytes that responded with propagative activity to increases in either NT, few were responsive with propagative activity increases to both NTs in WT networks (Fig. 4l). In fact, the number of astrocytes responsive to both NTs is not significantly different from chance, indicating that how an astrocyte in the network responds to one NT provides no information about how that same astrocyte will respond to the other. Further, when uncaging less glutamate in a different set of local networks (Extended Data Fig. 7a,b), the response profile of an individual astrocyte to three sequential rounds of NT release at the same location was variable (Extended Data Fig. 7c). This was a controlled comparison, as average increases in event frequency occurred over a similar time course (Extended Data Fig. 7d) and baseline activity was comparable in local network astrocytes across rounds (Extended Data Fig. 7e). As propagative response to a particular NT does not predict the response to the other NT or to sequential stimulation by the same NT, we next looked for metrics that instead might predict astrocyte network responses. Astrocyte Ca^2+^ activity can depend on prior and current Ca^2+^ levels^10,43,44^, which led us to investigate whether network-level propagative responses were linked to ongoing network activity. To do so, we examined whether the composition of baseline (1 min) activity in the WT network influenced the network-level response to uncaging (Extended Data Fig. 5e). Here, cells with a higher fraction of propagative events at the baseline (relative to all baseline events) exhibited a lower probability of responding to either GABA or glutamate (Fig. 4m, left, Methods and Extended Data Fig. 5i–k). By contrast, overall baseline event rate did not alter responses to either NT (Fig. 4m, right). Thus, in addition to differentiating the local astrocyte network response to GABA or glutamate, these correlational results indicate that propagative events may bias the astrocyte network’s subsequent responses to NTs.

## Discussion

Single-astrocyte simulation can cause long-lasting changes in neuronal activity and plasticity extending tens to hundreds of micrometres from the stimulation site^15,16,40^, but the mechanism(s) that drive distributed effects have not been well defined. Here, a brief, spatially restricted NT input leads to long-lasting, network-wide changes in astrocyte Ca^2+^, an effect facilitated by gap junctions. These findings could bridge the spatiotemporal gap between transient, local astrocyte stimulation and sustained, distributed effects on neurons, although the spatial extent of astrocyte network activation remains open because astrocyte Ca^2+^ changes extend beyond our FOV. What might be an effect of restricted NT inputs causing prolonged and distributed responses? For coordinated behaviour and learning, neuronal signals are integrated over seconds and minutes^45^. Models of neural integration that rely solely on neuronal activity require fine-tuned positive feedback loops to allow for integration over periods longer than tens of milliseconds^46^. Although recurrent neuronal connections enable temporal integration, astrocyte networks provide another possible mechanism to integrate inputs over long time periods^12,47,48^, linking the milliseconds timescale of neurons and the seconds-to-minutes timescales of behaviour.

Both GABA and glutamate uncaging led to sustained, far-reaching changes in astrocyte network Ca^2+^ activity, but propagative activity differentiated responses to each (Fig. 4n). Propagative events may facilitate the integration of information across cellular compartments to allow coordinated modulation of groups of nearby synapses^49^ or spatiotemporal integration of inputs across individual cells^44^. Stimulation by glutamate consistently led to greater increases in propagative activity (Figs. 1h and 4b,f,h), suggesting that cortical astrocytes are more responsive to glutamatergic than GABAergic signalling, as described for other brain regions^17^. Heightened astrocyte sensitivity to glutamate may mirror structural organization in the cortex, where astrocyte processes are closer to glutamatergic than GABAergic synapses^50^, potentially reflecting astrocytes’ key role in extracellular glutamate uptake. As surface mobility of astrocytic glutamate transporters depends on intracellular Ca^2+^ (ref. ^51^), a more robust Ca^2+^ response to glutamate may allow astrocytes to efficiently take up extracellular glutamate by increasing glutamate transporter mobility.

Astrocyte network responses to glutamate and GABA were context-dependent: responses to both NTs were lower when baseline activity had a high fraction of propagative events (Fig. 4m). Thus, as glutamatergic input preferentially recruits propagative events in the surrounding astrocyte network (Fig. 4b,f,h), it may also suppress subsequent responses to NT inputs. Although this result remains correlational, it indicates that astrocyte networks may implement combinatorial logic, integrating NT inputs across the local network by disseminating information through specific subtypes of Ca^2+^ activity.

Although most astrocytes and local networks increase Ca^2+^ in response to NT uncaging, a subset do not respond to direct or remote uncaging. This heterogeneity may be shaped by the activity state of the astrocyte and connected network during stimulation or by the subcellular location of uncaging. Alternatively, only a subset of astrocytes may be equipped to respond to NTs, given the molecular heterogeneity of astrocytes^52,53^. Future experiments imaging astrocyte responses to NTs, followed by spatial transcriptomics, could elucidate how cellular machinery may underlie heterogeneous responses.

Here, astrocytic gap junctions contribute to network activity changes, and may also regulate Ca^2+^ activity in individual cells. Molecules, including Ca^2+^ and IP_3_, can diffuse through gap junctions^54^. IP_3_ is required for Ca^2+^ release from internal stores^55^, and Ca^2+^ itself regulates Ca^2+^ release from internal stores through calcium-induced calcium release. Here, reduced gap junctional coupling between astrocytes may have altered cytosolic Ca^2+^ and IP_3_ concentrations, which could impact Ca^2+^ release from internal stores and shape Ca^2+^ dynamics within individual cells.

## Methods

### Animals

Experiments were carried out using young adult mice, in accordance with protocols approved by the University of California, San Francisco Institutional Animal Care and Use Committee. Animals were housed in a 12:12 light/dark cycle with food and water provided ad libitum. Animal housing rooms were kept at 68–74 °F and 30–70% humidity. Male and female mice were used whenever available. Transgenic mice used in this study were *Cx43*^*fl/fl*^ mice^56^ from the Bhattacharya Lab (University of California, San Francisco, USA) and EAAT2-tdT mice^57^ from the Yang Lab (Tufts University, USA). For in vivo imaging, all experiments were carried out at the same time each day.

### Surgical procedures

For viral expression for ex vivo experiments, neonatal Swiss Webster or C57Bl/6 (P0–3) mice were anaesthetized on ice for 3 min before injecting viral vectors (AAV5.GfaABC_1_D.GCaMP6f.SV40 (Addgene, 52925-AAV5), AAV9.hGfap.pinkFlamindo, pENN.AAV9.Gfap.iGluSnFr.WPRE.SV40 (Addgene, 98930-AAV9) or AAV5.GFAP(0.7).RFP.T2A.iCre (Vector Biolabs, 1133)). Pups were placed on a digital stereotax and coordinates were zeroed at lambda. Four injection sites in a 2 × 2 grid pattern over V1 were chosen. Injection sites were 0.8–0.9 mm and 1.6–1.8 mm lateral, and 0 and 0.8–0.9 mm rostral. At each injection site, 30–120 nl of virus was injected at a rate of 3 nl s^−1^ at two depths (0.1 mm and 0.2 mm ventral/below pia) using a microsyringe pump (UMP-3, World Precision Instruments).

For viral expression for the in vivo experiments, adult C57BL/6 mice (2–4 months at the time of surgery) were administered dexamethasone (5 mg kg^−1^, subcutaneously) >1 h before surgery, and anaesthetized using 1.5% isoflurane (Patterson Veterinary Supply, 78908115). After hair removal and three alternating swabs of 70% ethanol (Thermo Fisher Scientific, 04-355-720) and Betadine (Thermo Fisher Scientific, NC9850318), a custom-made titanium headplate was attached to the skull using cyanoacrylate glue and C&B Metabond (Parkell, S380). A 3-mm craniotomy was made over the right visual cortex. Virus was injected at two sites in the right visual cortex at coordinates centred on +2.4 mm and +2.7 mm medial–lateral, +0.35 mm and +0.65 mm anterior–posterior and −0.3 mm dorsal–ventral from lambda. A 300 nl volume of AAV5.GfaABC_1_D.GCaMP6f.SV40 (Addgene, 52925-AAV5) was injected at each site through a glass pipette and microsyringe pump (UMP-3, World Precision Instruments). After allowing at least 10 min for viral diffusion, the pipette was slowly withdrawn and a glass cranial window was implanted using a standard protocol.

### Ex vivo two-photon imaging and uncaging

Coronal, acute V1 slices (400-µm thick) from P28–32 (bath-application) and P27–42 (uncaging) mice were cut with a vibratome (VT 1200, Leica) in ice-cold slicing solution containing (in mM) 27 NaHCO_3_, 1.5 NaH_2_PO_4_, 222 sucrose, 2.6 KCl, 2 MgSO_4_, 2 CaCl_2_. Slices were transferred to pre-heated, continuously aerated (95% O_2_/5% CO_2_) standard artificial cerebrospinal fluid (ACSF) containing (in mM) 123 NaCl, 26 NaHCO_3_, 1 NaH_2_PO_4_, 10 dextrose, 3 KCl, 2 MgSO_4_, 2 CaCl_2_. Younger mice were sliced in the same solutions for GCaMP bath-application of LY379268 and baclofen (P20–25), Pink Flamindo (P20–22) and GluSnFR (P14–17). Slices were kept at room temperature until imaging. Bath-application experiments were carried out at room temperature and two-photon uncaging experiments were carried out at 29 °C using an in-line heater (TC-324B and SH-27B, Warner Instruments). To block neuronal action potentials during all slice imaging experiments, except for GluSnFr recordings, tetrodotoxin (TTX; 1 µM) was added to the ACSF >10 min before imaging and remained in the circulating bath for the duration of the experiments.

Images were acquired on an upright microscope (Bruker Ultima IV) equipped with two Ti:sapphire lasers (MaiTai, SpectraPhysics). Laser beam intensities were modulated using two independent Pockels cells (Conoptics) and images were acquired by scanning with linear galvanometers. Images were acquired with a 16×, 0.8 NA (Nikon) or a 40×, 0.8 NA (Nikon) water-immersion objective via photomultiplier tubes (Hamamatsu) using PrairieView (Bruker) software. For GCaMP imaging, 980-nm excitation and a 515/30 emission filter were used. For RFP imaging, 980-nm excitation and a 605/15 emission filter were used. For Pink Flamindo and Alexa Fluor 594 imaging, 1,040-nm excitation and a 605/15 emission filter were used. Images were acquired at a 1.42 Hz frame rate, 512 × 512 pixels and 0.64–1.61 µm per pixel resolution. For GluSnFR imaging alone, images were acquired at a 6.21 Hz frame rate, 200 × 200 pixels and 0.64 µm per pixel resolution, with 980-nm excitation and a 515/30 emission filter.

For bath-application experiments, a 5-min baseline was recorded to monitor spontaneous activity, after which receptor agonists were added along with a fluorescent dye (Alexa Fluor 594 hydrazide) to assess the time at which drugs reached the imaging field (except for Pink Flamindo owing to spectral overlap). An ACSF washout period (>10 min), followed by a TTX incubation period (>10 min), occurred between trials when imaging the same slice sequentially for bath-application of different receptor agonists or uncaging of different RuBi subtypes. To account for any changes resulting from prior agonist exposure or uncaging, we alternated the order of agonists between concentrations or RuBi subtypes between slices.

For simultaneous two-photon imaging and uncaging, a second Ti:sapphire laser beam was tuned to 800 nm and controlled using an independent set of linear galvanometers from those used for scanning. Laser beam intensity was modulated using an independent Pockels cell (Conoptics) to achieve a power measurement of about 2–8 mW at the slice. The beam paths for imaging and uncaging were combined after the linear galvanometers using an 855-longpass dichroic mirror (T855lpxr, Chroma). The uncaging laser was calibrated each experimental day by burning spots into a fluorescent slide. RuBi compounds (300 µM) and TTX (1 µM) were added to the ACSF >10 min before imaging each slice. FOVs were chosen on the basis of the location of GCaMP expression, which was often biased to (brighter in) deeper cortical layers (distance of FOV from pia: 615 ± 196 µm (mean ± s.d., *n* = 121 FOV)). Before imaging at each FOV, a 60-s period was recorded to identify potential uncaging sites. Areas of GCaMP expression that exhibited moderate levels of spontaneous Ca^2+^ activity were chosen as uncaging sites. For FOVs with sequential GABA and glutamate uncaging, a continuous 5-min recording was used to monitor activity in each FOV. For FOVs with three sequential rounds of glutamate uncaging, a continuous 12.5-min recording was used to monitor activity in each FOV. Each recording began with a 2.5-min baseline period, and at the 2.5-min mark, NT was uncaged with 10 × 100 ms pulses, 100 ms apart. Sequential recordings of GABA and glutamate uncaging within the same FOV were separated by >20 min. Rounds of sequential glutamate uncaging were separated by ≥25 min. Voltage from the uncaging laser Pockels cell was recorded to mark the time of uncaging pulses. As RuBi–GABA and RuBi–glutamate are light-sensitive, care was taken to ensure experiments were carried out in minimal light. The computer screen and redshifted headlamp were covered with two layers of red filter paper (Roscolux number 27 filter, Rosco) and all indicator lights on equipment were covered.

### In vivo two-photon imaging

In vivo two-photon imaging was carried out on the same microscope as ex vivo imaging, using a Nikon 16×, 0.8 NA water-dipping objective with a ×2 optical zoom (frame rate: 1.7 Hz, FOV: 412 µm^2^, resolution: 512 × 512 pixels). Animals were given >1 week after surgery for recovery and viral expression. They were then habituated on a custom-made circular running wheel over at least 2 days, and for a cumulative time of at least 2.5 h, before recording. After habituation, mice were head-fixed on the wheel and movements were recorded by monitoring deflections of coloured tabs on the edge of the wheel using an optoswitch (Newark, HOA1877-003). To compute wheel speed, a detected break in the optoswitch circuit was determined when the absolute value of the derivative of the raw voltage trace was at least 2 standard deviations above the mean. For recordings with little movement (s.d. < 0.1), this threshold generated false positives, so a set threshold of 0.1 was used. The number of breaks in the optoswitch circuit per second was then calculated, and using the circumference and number of evenly spaced coloured tabs at the edge of the wheel, the wheel speed was determined and used for all subsequent analyses using speed. Movement periods were defined by wheel speed ≥10 cm s^−1^ and movement bouts that were separated by ≤2 s were considered one event. To ensure that movement-related dynamics were not included in stationary analysis, data were excluded from <10 s around identified movement periods. GCaMP was imaged with 950-nm excitation light and a 515/30 emission filter. Recordings lasted 30 min.

### Ex vivo pharmacology

The following concentrations of each pharmacological reagent were used for experiments as indicated in the text: tetrodotoxin citrate (TTX, 1 µM, Hello Bio); carbenoxolone disodium (CBX, 50 µM, Tocris Bioscience); *R*(+)-baclofen hydrochloride (5–100 µM, Sigma-Aldrich); (1*S*,3*R*)-ACPD (t-ACPD, 5–100 µM, Tocris); LY379268 disodium salt (100 µM, Tocris); Alexa Fluor 594 hydrazide (0.1–2 µM, Thermo Fisher Scientific); RuBi GABA trimethylphosphine (RuBi-GABA-Pme_3_, 300 µM, Tocris); RuBi–Glutamate (300 µM, Tocris); CGP 55845 hydrocholoride (10 µM, Tocris); and LY341495 (10 µM, Tocris).

### Immunohistochemistry and image quantification

After recording, slices from two-photon imaging experiments were immersed in 4% PFA for 30 min and switched to 30% sucrose for 1 day at 4 °C before being embedded in OCT and stored at −80 °C. Slices were re-sectioned coronally at 40 µm on a cryostat and then stored in cryoprotectant at −20 °C until staining. For immunohistochemistry, sections were washed three times in 1× PBS for 5 min and permeabilized for 30 min with 0.01% Triton-X in 1× PBS. Sections were next blocked with 10% NGS (Abcam) for 1 h and incubated overnight with primary antibodies at 4 °C in 2% NGS. The next day, they were washed three times in 1× PBS before incubating with secondary antibodies for 2 h at room temperature. Sections were washed three times in 1× PBS for 5 min before being mounted on slides with Fluoromount-G (SouthernBiotech).

To validate reduction of Cx43 protein in astrocytes transduced with adeno-associated viruses to express GCaMP–GFP and Cre–RFP, primary antibodies to anti-Cx43 (1:1,500, rabbit, Sigma-Aldrich), anti-GFP (1:3,000, chicken, Abcam) and anti-mCherry (1:2,000, rat, Thermo Fisher Scientific) in 2% NGS were used. Secondary antibodies include anti-rabbit Alexa Fluor 405, anti-chicken Alexa Fluor 488 and anti-rat Alexa Fluor 555 (all Thermo Fisher Scientific), which were all used at 1:1,000 dilution. ×60 multi-channel *z*-stack images were acquired on a CSU-W1 spinning-disc confocal microscope (Nikon) using MicroManager from V1 in which adeno-associated viruses were injected. To quantify loss of Cx43 in RFP^+^ and RFP^−^ astrocytes, Fiji (ImageJ) was used. Through batch processing, cell maps were created through a semi-automated pipeline to segment astrocytes, with post hoc ROI adjustments for vasculature artefacts. Multi-channel *z*-stacks were split into 405, 488 and 555 channels, and unstacked into sequential 8-bit *z*-plane images. For each *z*-plane, RFP^+^ and RFP^−^ astrocytes were detected using a Gaussian blur (sigma = 3), thresholding using the Phansalkar method (radius = 1,000) and applying ImageJ’s Analyze Particles command (size > 175 µm^2^, circularity = 0–0.60) to outline ROIs using the wand tool. Corresponding Cx43 images were binarized and the Fiji plugin SynQuant^58^ was used to detect Cx43 puncta number within each RFP^+^ and RFP^−^ astrocyte in a *z*-plane’s cell map. Puncta counts were normalized to astrocyte area, and the normalized count from each *z*-stack was averaged for each slice.

### Two-photon image and data analysis

Individual-astrocyte cell maps for time-series images were created in Fiji using the following process. For each FOV, an 8-bit *z*-projection of the time series was created. The *z*-projection was binarized using the Auto Local Threshold feature, using the Niblack method and a radius of 30 or 75, for 16× and 40× images, respectively. Cell maps were drawn on binarized images using a combination of the Lasso and Blow Tool and the freehand drawing tool in Fiji, and verified on the *z*-projected image. Cell maps were also verified against a static indicator of astrocyte morphology when available (EAAT2-tdT^+^ mice for bath-application of LY379268 and baclofen; GFAP(0.7)-RFP-T2A-iCre in *Cx43*-floxed mice). To load cell masks into AQuA, regions were saved to the ROI manager and filled in with a colour. The regions were projected onto a black image the same size as the original (512 × 512 pixels). The overlay of regions was flattened, converted to an 8-bit image and saved as a tiff. For the 12.5-min recordings with sequential rounds of glutamate uncaging, drift of the slice in *x* and *y* was corrected post hoc using moco^59^.

#### AQuA

GCaMP and GluSnFR two-photon image sequences were analysed using AQuA^8^ and custom MATLAB (MATLAB R2018b) and Python (v3.8.18) code. Signal detection thresholds were adjusted for each video to account for differences in noise levels after manually checking for accurate AQuA detection. Cell maps were loaded into AQuA to define cells consistently over multiple time series featuring the same FOV. For all bath-application experiments, the direction of pia was marked as anterior. For two-photon uncaging experiments, the uncaging site was marked as a 3 × 3-pixel landmark.

#### Bath-application event-based analysis

For baclofen and t-ACPD Ca^2+^ imaging experiments, event count per frame was quantified by counting all AQuA-detected events, new or ongoing, in each frame (Fig. 1c). Percentage of field active values were calculated by recording the number of active pixels in each frame, as determined by the frame-by-frame footprints of AQuA-detected events. These values were normalized by the total number of active pixels in the recording and multiplied by 100. For the percentage of field active dose–response curve (Fig. 1e), the percentage of field active values from all frames within the chosen time points were averaged by slice. Event propagation was calculated by summing the growing propagation from all cardinal directions, using the AQuA feature propGrowOverall. For dose–response curves for discrete event features (area, duration and propagation; Fig. 1f–h), all detected Ca^2+^ events within the chosen time points were averaged by slice.

The frame in which the agonist entered the recording chamber was estimated using fluorescence from Alexa Fluor 594 (0.1–2 µM, added to the ACSF reservoir along with the agonist) by using the maximal curvature method on frames 1–600 of the raw Alexa Fluor 594 fluorescence trace. The maximum curvature method^60^ defines the onset fluorescence changes as the point of maximum curvature during the rising phase of the signal. To identify this point, traces were fitted using a modified Boltzmann’s sigmoidal equation:

$$f\left(x\right)=\frac{a}{1+{{\rm{e}}}^{\left(b-x\right)/d}}+c$$

in which *a* is the difference between the minimum and the maximum fluorescence, *b* is the inflection point, *c* is the baseline fluorescence and *d* is the slope, using a nonlinear least-squares algorithm (Levenberg–Marquardt) in MATLAB (Mathworks). Next, the frames of maximum curvature were calculated by setting the fourth derivative of the fitted curve equal to zero and solving for its three solutions. The earliest frame identified out of these three solutions was recorded as the onset frame.

#### Bath-application ROI-based analysis

Pink Flamindo and GCaMP imaging experiments were analysed using ROI-based approaches in Fiji. To identify responding cells in Pink Flamindo experiments (Extended Data Fig. 1j), sigmoidal curves were fitted to Δ*F*/*F* traces using the modified Boltzmann’s sigmoidal equation detailed above. Cells were defined as responding if the difference between the minimum and maximum values of the fit curve (*a* in the Boltzmann’s sigmoidal equation) was greater than the baseline noise (3 s.d. of baseline fluorescence). Responding cells were defined as increasing if $f\left({x}_{{\rm{start}}}\right) < f\left({x}_{{\rm{end}}}\right)$ and decreasing if $f({x}_{{\rm{start}}}) > f({x}_{{\rm{end}}})$.

To identify fluctuations in Pink Flamindo and GCaMP fluorescence (Extended Data Fig. 1k), peaks were detected from Δ*F*/*F* traces from individual cells. Peaks were counted if they were 3 s.d. above the mean baseline fluorescence, and had a minimum peak width of 5 frames and a minimum distance of 10 frames between detected peaks. The baseline period was defined as all frames before the frame of agonist entry. For GCaMP, all astrocytes exhibiting ≥1 AQuA-detected event during the 10-min recording were run through peak finding. For Pink Flamindo, all detected astrocytes were run through peak finding.

For GCaMP experiments, the frame in which the agonist entered the recording chamber was estimated using the fluorescence from Alexa Fluor 594 (0.1–2 µM) added to the ACSF reservoir along with the agonist. The time of agonist entry in the recording chamber was estimated by identifying the first frame in which Alexa Fluor 594 fluorescence reached ≥3 s.d. above the baseline mean (frames 1–300); only frames >375 were considered for evaluation of exceeding the threshold. For Pink Flamindo experiments, dye was not added with the agonist to avoid spectral overlap. The time of agonist entry in the recording chamber was estimated by adding 90 frames (the average number of frames for ACSF to travel from the reservoir to the recording chamber) to the frame in which the agonist was added to the reservoir of ACSF.

#### Two-photon uncaging event-based analysis

Individual astrocytes were excluded from analyses (Figs. 2–4 and Extended Data Figs. 2–7) if the baseline event rate changed significantly. Changes in baseline event rate for each cell were determined by carrying out Poisson regression of events in 1-s bins during the period from 90 to 10 s pre-uncaging. Cells with a regression coefficient with *P* < 0.1 at the baseline and with >5 AQuA-detected events throughout the recording were excluded from all analyses, except for Extended Data Fig. 7d RuBi–glutamate uncaging control. ∆*F*/*F* values in raster plots (Figs. 2h and 3c) were calculated using the AQuA output dffMatFilter(:,:,2), the ∆*F*/*F* traces from events after removing the contributions from other events in the same location. Cells (Fig. 2h) or local astrocyte networks (Fig. 3c) were sorted on the basis of the onset time of a response following uncaging. A response was defined as the first post-stimulus peak greater than or equal to the threshold (mean baseline Δ*F*/*F* + 3 s.d.), with thresholds calculated by cell or local network using 90–0 s before uncaging. For Fig. 3c, the *z* score of each local network was calculated using the mean Δ*F*/*F* from AQuA-detected events in the network, using a baseline period of 90–0 s before uncaging. For the Sholl-like analysis (Fig. 3h), events were sorted into 50-µm bands radiating out from the uncaging site using the minimum distance between an event and the uncaging site at event onset (using the AQuA output ftsFilter.region.landmarkDist.distPerFrame). The 50-µm-wide bands began 25 μm and ended 175 µm from uncaging, as events <25 μm from the uncaging site occur within the stimulated astrocyte and those >175 μm from the uncaging site can be outside the FOV (Extended Data Fig. 3i). The periods 90–0 s before and 0–150 s after uncaging were used to calculate the change in event number per 30 s per band. To categorize events as propagative or static (Fig. 4d–m and Extended Data Figs. 5b–j, 6 and 7c), the total propagation distance of each event was computed by summing the growing propagation from all cardinal directions, using the AQuA feature propGrowOverall. Events were categorized as propagative if the total propagation distance was >1 µm.

### Statistics and reproducibility for representative micrographs and spatial heat maps

Representative micrographs were chosen from experiments repeated with similar results from the following *n*—Fig. 1b: *n* = 4 slices, 4 mice; Fig. 2c: *n* = 72 trials, 12 recordings, 4 slices, 2 mice; Fig. 2f,g: *n* = 28 astrocytes, 7 slices, 4 mice (note the heterogeneity shown in Fig. 2h for individual astrocyte responses to NT); Fig. 3b: *n* = 28 FOV, 7 slices, 4 mice; Fig. 4a: *n* = 28 FOVs, 7 slices, 4 mice; Fig. 4c: *n* = 15 recordings, 5 mice; Extended Data Fig. 1i: *n* = 8 slices, 3 mice; Extended Data Fig. 3b: *n* = 91 FOVs, 16 slices, 8 mice; Extended Data Fig. 5a: *n* = 28 FOVs, 7 slices, 4 mice.

### Statistics for Figs. 1–3 and associated Extended Data figures

All statistical tests used and the exact *n* values can be found for each figure in the corresponding figure legend. Adjustments for multiple comparisons using Bonferroni–Holm correction were implemented using fwer_holmbonf^61^. Significance levels were defined as follows: NS: *P* ≥ 0.05; *P < 0.05; **P < 0.01; ***P < 0.001.

#### Permutation testing

Statistical significance for time-series data was computed using permutation testing with custom-written code in MATLAB. A total of 10,000 permutations were run and one- or two- sided *P* values for each time point were calculated. *P* values were corrected for multiple comparisons using the Benjamini–Yekutieli procedure (implemented using ref. ^62^) with a false discovery rate of ≤0.05.

Data were shuffled (permuted) in the following way. To test change in event number per cell (Fig. 1c and Extended Data Figs. 2b and 3g,h), events were shuffled independently for each active cell (≥1 AQuA-detected event) in each time series. For each active cell, events were randomly placed in time bins spanning the duration of the recording (time bins of 60 s (Fig. 1c) and 30 s (Extended Data Figs. 2b and 3g,h)) and the change in number of events per time bin was calculated as for the experimental data. Permuted changes in event number per cell were averaged across active cells in each time series and across all time series to obtain the permuted mean for one round of permutation testing.

To test change in event number per band (Fig. 3h), permutation tests were run separately for each band and events were shuffled independently for each time series. For each time series, events from the tested band were randomly placed in 30-s time bins spanning the duration of the recording, and the change in event number per 30 s was calculated as for the experimental data. Permuted changes in event number per 30 s were averaged across all time series to obtain the permuted mean for one round of permutation testing. To test the magnitude of change in experimental data versus permuted data, two-sided *P* values were calculated as:

$$\frac{({\rm{number}}\,{\rm{of}}\,{\rm{times}}| {\rm{permuted}}\,{\rm{change}}| \ge | {\rm{experimental}}\,{\rm{change}}| )+1}{{\rm{number}}\,{\rm{of}}\,{\rm{permutations}}+1}$$

For testing increases in ∆*F*/*F* (Extended Data Fig. 1d), frames were shuffled independently for each time series. For each time series, the average ∆*F*/*F* per frame from active regions (≥1 AQuA-detected event in either condition (baclofen or t-ACPD)) was calculated, the frame order was shuffled, and the mean ∆*F*/*F* per 30 s was calculated. Permuted mean ∆*F*/*F* was averaged across all time series to obtain the permuted mean for one round of permutation testing. To test the magnitude of increases in experimental data versus permuted data, one-sided *P* values were calculated as:

$$\frac{({\rm{number}}\,{\rm{of}}\,{\rm{times}}\,{\rm{the}}\,{\rm{permuted}}\,{\rm{mean}}\ge {\rm{the}}\,{\rm{experimental}}\,{\rm{mean}})+1}{{\rm{number}}\,{\rm{of}}\,{\rm{permutations}}+1}$$

### Statistics for Figs. 3i–l and 4, and associated Extended Data figures

#### Two-photon uncaging grid-based ROI analysis

Grid-based ROIs were determined by dividing the 300 × 300 µm imaging field into a uniform 20 × 20 µm grid (Fig. 3i–l). Each identified Ca^2+^ event was assigned to the ROI in which the centroid of its spatial footprint was located. ROIs with any baseline events were identified as ROIs with ≥1 events in the baseline window 60–0 s before uncaging. Active ROIs for each NT were identified as ROIs with a ≥50% increase in event rate in the window 0–120 s after uncaging for that NT, as compared with the rate during the baseline window. Active ROIs were a subset of ROIs with baseline events, as the relative increase in event rate is not defined when there are no baseline events, which results in division by 0. The distance from the uncaging site to each active ROI was determined using the Euclidean distance between the uncaging site, at (0, 0), and the centre of each grid ROI (Fig. 3j).

The fraction of overlap (that is, Jaccard index) *O*_*i*_ between active ROIs for GABA and glutamate was determined for the *i*th FOV by

$${O}_{i}=\frac{\left|{A}_{\text{GABA},i}\cap {A}_{\text{glutamate},i}\right|}{\left|{A}_{\text{GABA},i}\cup {A}_{\text{glutamate},i}\right|}$$

in which *A*_GABA,*i*_ and *A*_glutamate,*i*_ are the sets of active ROIs for GABA and glutamate, respectively, and and |*X*| denotes the number of elements of the set *X*. The overall fraction of overlap *O* between active ROIs for GABA and glutamate was computed as the mean of the individual *O*_*i*_ (Fig. 3l).

To determine whether the observed fraction of overlap was expected because of chance, a distribution of *n* = 10,000 surrogate fractions of overlap was computed. The *k*th surrogate value, ${\widetilde{O}}^{(k)}$, was computed as above, but replacing, for each NT, the set of active ROIs *A*_NT,*i*_ with a new set, ${\widetilde{A}}_{\text{NT},i}^{(k)}$, which was chosen as a random subset of size |*A*_NT,*i*_| of the set of ROIs with any baseline events for that NT. The *P* value for this comparison was estimated^63^ as

1 $$P=\frac{({\rm{number}}\,{\rm{of}}\,{\widetilde{O}}^{(k)}\ge O)+1}{n+1}$$

#### Propagation probability (Fig. 4b)

Each Ca^2+^ event was identified as growing in the depth axis if the frontier of that event’s spatial footprint extended over time either towards the pia or away from the pia, as determined by the posterior and anterior component of the propGrowOverall metric computed through segmentation by AQuA^8^.

The probability of events growing in the depth axis was computed separately for recordings of GABA and glutamate uncaging within each examined time window. Probabilities were estimated for the baseline window of 60–0 s before uncaging, as well as in non-overlapping 30-s bins ranging from 0 to 150 s post-uncaging, by computing the fraction of events that were identified as growing in the depth axis among all events from all recordings within the relevant time window. The change in the probability of events growing in the depth axis was then estimated for each bin as the difference between the fraction of events growing in the depth axis for that bin versus for the baseline period.

To empirically determine the distribution of each of these estimators, we carried out this same procedure for estimating the probability of events growing in the depth axis for each NT and time bin on surrogate data generated by hierarchically bootstrapping Ca^2+^ event data, for which the hierarchy was sampled cells within sampled recordings (that is, all events for an individual recording of one individual cell always remained together); this procedure was repeated 10,000 times for each bin. Standard errors were computed as the standard deviation of these empirical distributions.

To determine the probability of observing effects this large under a null hypothesis of no effect of time on the probability of events growing in the depth axis, we computed the distribution of the estimator under an imposed condition in which the overall temporal structure of astrocyte Ca^2+^ events was disrupted. To do this, we carried out the same procedure as above for estimating the probability of events growing in the depth axis for each bin, but on surrogate data generated by circularly shifting the timing of each individual cell’s Ca^2+^ events from 90 s before to 150 s after uncaging by its own independent, uniform random shift between 0 s and 240 s; this procedure was repeated *n* = 10,000 times for each bin. As it was unknown whether event propagation would increase or decrease post-uncaging, two-sided *P* values were estimated^63^ as

2 $$P=\frac{({\rm{number}}\,{\rm{of}}\,| {\widetilde{X}}^{(k)}| \ge | X| )+1}{n+1}$$

in which *X* denotes the actual observed value of the estimator, and each ${\widetilde{X}}^{(k)}$ is the value of the estimator computed from the *k*th shifted dataset. These *P* values were adjusted across tested time bins and NTs using the Benjamini–Hochberg procedure to obtain *q* values, as implemented in statsmodels 0.12.2 (ref. ^64^).

#### Event feature changes (Extended Data Fig. 4a,b)

Each Ca^2+^ event is assigned several metrics by AQuA-segmentation^8^, including size (area, perimeter, circMetric (circularity, based on area and perimeter)), amplitude (dffMax) and dynamics (rise19 (rise time), fall91 (fall time), decayTau (decay time constant), width11 (duration)). For each non-propagation metric, the mean metric value among events was computed separately for recordings of GABA and glutamate uncaging for the baseline window 60–0 s before uncaging, as well as in non-overlapping 30-s bins from 0 to 150 s post-uncaging. For each bin, the ratio of that bin’s mean metric value to the baseline mean metric value was computed.

AQuA metrics also capture information about events’ directional propagation. Each Ca^2+^ event was identified as growing or shrinking in each cardinal direction if the frontier of that event’s spatial footprint extended or receded, respectively, over time in that direction, as determined by the components of the propGrowOverall and propShrinkOverall metrics. For each propagation metric, the change in the probability of events growing or shrinking in each axis was computed separately for recordings of GABA and glutamate uncaging within each examined time window, as in the section entitled “Propagation probability”, but using the ‘growing’ or ‘shrinking’ identifiers for each cardinal direction.

To empirically determine the distribution of each of these estimators (that is, binned post/baseline ratio for non-propagation metrics, binned change in growing or shrinking probability for propagation metrics), we carried out the same procedures for computing each metric’s relevant estimators for each NT and time bin outlined above on 10,000 surrogate datasets generated by hierarchically bootstrapping Ca^2+^ event data, as described in the section entitled “Propagation probability”. Standard errors were computed as the standard deviation of these empirical distributions.

To determine the probability of observing effects this large under a null hypothesis of no effect of time on the probability of events growing in the depth axis, we computed the distribution of each estimator under 10,000 realizations of an imposed condition in which the overall temporal structure of astrocyte Ca^2+^ events was disrupted by randomly circularly shifting each cell’s Ca^2+^ events, as described in the section entitled “Propagation probability”. As it was unknown whether event propagation would increase or decrease post-uncaging, two-sided *P* values were estimated using equation (2) above^63^. These *P* values were adjusted across tested time bins and NTs using the Benjamini–Hochberg procedure to obtain *q* values, as implemented in statsmodels 0.12.2 (ref. ^64^).

#### Comparison of in vivo and ex vivo event propagation (Fig. 4d)

Events were categorized as propagative or static, as outlined in the section ‘Two-photon uncaging event-based analysis’. The fraction of propagative events observed in vivo and ex vivo was calculated using baseline events. Ca^2+^ events in in vivo recordings were labelled as baseline events if they occurred during periods when the mouse was stationary, as outlined in the section entitled “In vivo two-photon imaging”. Ca^2+^ events in ex vivo recording were labelled as baseline events if they occurred in neighbouring astrocytes (that is, cells not directly stimulated by NT) during the 60–0 s before NT uncaging.

To determine the distribution of the two median propagative event fractions empirically, we computed the medians of 10,000 bootstrapped samples of the per-recording fractions for each setting. Standard errors for each statistic were determined from the standard deviations of these empirical distributions.

#### Computing rate changes for propagative and static events (Fig. 4f,j and Extended Data Fig. 6b,c)

The overall rates of propagative and static events for neighbouring astrocytes were computed separately for recordings of GABA and glutamate uncaging.

For each event class (that is, propagative and static events), for each recording, the event rate was computed in each time window as the total number of events from all neighbouring cells in that recording in the given time window divided by the duration of that time window. These recording-level rates were computed for the baseline window of 60–0 s before uncaging and in non-overlapping 30-s bins ranging from 0 to 150 s post-uncaging. For each recording, the relative rate of propagative and static events was computed for each time bin as the ratio of the event rate for the given event class in that time bin divided by the corresponding event rate in the baseline window. For each time bin, the overall relative rate was estimated as the median of the per-recording relative rates in that time bin.

To determine the distribution of each of these relative rate estimators empirically, we carried out this same procedure for estimating relative event rates on surrogate data generated by hierarchically bootstrapping Ca^2+^ event data 10,000 times for each bin (as in the section ‘Propagation probability’). Standard errors were computed as the standard deviation of these empirical distributions.

To determine the probability of observing effects this large under a null hypothesis of no effect of time post-uncaging on the rate of astrocyte Ca^2+^ events, we computed the distribution of the relative rate estimators under an imposed condition in which the overall temporal structure of astrocyte Ca^2+^ events was disrupted using a random circular shift of the events in each cell, as in Fig. 4b; this procedure was repeated *n* = 10,000 times for each bin. Motivated by results in bath-application experiments above demonstrating robust aggregate astrocyte Ca^2+^ event rate increases in response to agonism of glutamate or GABA receptors (Fig. 1c), we estimated one-sided *P* values from these permuted datasets, as in equation (1). These *P* values were adjusted across tested time bins and NTs using the Benjamini–Hochberg procedure to obtain *q* values, as implemented in statsmodels 0.12.2 (ref. ^64^).

#### Determining responding cells on the basis of static and propagative events (Fig. 4h,k and Extended Data Fig. 6e,f)

The overall rates of propagative and static events were computed for each neighbouring astrocyte, with paired measurements made for recordings of GABA and glutamate uncaging. For each neighbouring astrocyte, for each event class (that is, propagative and static events), the event rate was computed in each time window as the total number of events from that cell in the given time window divided by the window’s duration (baseline window: 60–0 s before uncaging, response window: 0–120 s after NT uncaging; Extended Data Fig. 5c). Relative event rates were calculated as for Fig. 4f,j and Extended Data Fig. 6b,c. Cell-recording combinations with zero events of a given type in the baseline window were excluded for computation of relative rates of propagative (GABA: 36 recordings of cells (26.7% of total); glutamate: 37 (32.2%)) and static (GABA: 0; glutamate: 0) events, as the relative rate would require a division by zero and be undefined in those cases. Astrocytes were identified as ‘responders’ with a particular event type (that is, static or propagative) to GABA or glutamate if their relative rate of that type of event was ≥1.5 for the corresponding NT uncaging recording (Extended Data Fig. 5d). The fraction of astrocytes that were responders was computed for each individual recording, as well as the overall fraction of responders averaged across all recordings for each NT.

To determine the distribution of these overall responder fractions, we carried out this same procedure for estimating relative event rates on surrogate data generated by hierarchically bootstrapping Ca^2+^ event data 10,000 times (as in the section ‘Propagation probability’). Standard errors were computed as the standard deviation of these empirical distributions.

To determine whether there were significant differences between the overall responder fractions for GABA and glutamate, we computed the distribution of the difference between these two fractions under an imposed condition in which there was no systematic difference between GABA and glutamate. To do this, we carried out the same procedure as above for estimating the difference between the overall responder fractions for ‘GABA’ and ‘glutamate’, but on surrogate data generated by, for each cell, swapping the labels for ‘GABA’ and ‘glutamate’ responses from that in the experimental data with probability 1/2; this procedure was repeated 10,000 times. As it was unknown a priori whether GABA or glutamate would have a higher fraction of responder cells, a two-sided *P* value was estimated as in equation (2).

#### Decoding NT identity from propagative event responses (Fig. 4i)

To quantify the extent to which the observed difference in propagative event responses to uncaged glutamate and GABA enabled reliable identification of NT identity on a trial-by-trial basis, we built a simple classifier that took as input a single value, the relative change in propagative event rate across a FOV in the window 0–120 s post-uncaging relative to the window 60–0 s pre-uncaging, and classified that FOV as responding to glutamate if the value was greater than or equal to a set threshold, and GABA if the value was less than the threshold. To evaluate this classifier’s performance, we built a receiver operating characteristic curve by varying the classification threshold across the entire domain of the feature, and at each value of the threshold, computing the empirical true positive rate and false negative rate of the classifier. With the threshold fixed in the receiver operating characteristic analysis, the classifier did not have any remaining free parameters, so did not need to be trained on data and was therefore not a function of any of the data, obviating the need for cross-validation. We computed the AUC using the trapezoidal rule. To determine the distribution of the observed AUC statistic, we carried out this same analysis on 10,000 surrogate datasets generated by bootstrapping (that is, resampling FOVs with replacement). To determine whether the observed AUC statistic was above 0.5 (indicating completely non-informative decoding) to a degree greater than expected by chance alone, we carried out this same analysis on 10,000 surrogate datasets generated by permuting the NT labels.

#### Determining correlations between GABA and glutamate responses (Fig. 4l)

To determine whether individual cells’ responses to GABA and glutamate—as determined in Fig. 4h—were correlated, we computed the Spearman *ρ* between the binary paired responses to GABA and glutamate across cells that could be assessed in both conditions (that is, had >0 propagating baseline Ca^2+^ events in both recordings) using SciPy 1.6.2 (ref. ^65^). To determine the probability of observing a correlation at least this large under a null hypothesis of independence between cells’ responses for GABA and glutamate, we computed the Spearman *ρ* on surrogate data in which the identities of the cells’ responses to GABA and glutamate were independently permuted; this procedure was repeated 10,000 times. To maintain the ability to identify correlation or anticorrelation, we estimated a two-sided *P* value from these surrogate values, as in equation (2).

To complement this analysis, we computed the fraction of overlap (that is, Jaccard index) between the sets ${C}_{\text{GABA}}$ and ${C}_{\text{glu}}$ of cells that were responders to GABA and glutamate, respectively:

$$O=\frac{\left|{C}_{\text{GABA}}\cap {C}_{\text{glu}}\right|}{\left|{C}_{\text{GABA}}\cup {C}_{\text{glu}}\right|}$$

This statistic is larger when the fraction of overlap between responders for the two NTs is larger. To determine the probability of observing an overlap at least this large under a null hypothesis of independent responses for GABA and glutamate, we computed this same statistic, but on 10,000 permuted surrogate datasets, as above. To determine significant overlap, we estimated a one-sided *P* value from these surrogate values, as in equation (1).

#### Segregating responding cells on the basis of baseline propagation (Fig. 4m)

For each neighbouring astrocyte with propagative events during the baseline period of 60–0 s pre-uncaging, we computed the fraction of baseline events that were propagative (number of propagative baseline events/total number of baseline events). Separately for GABA and glutamate, we used the propagative fraction across all given astrocytes to define the threshold fraction of baseline propagative activity, *f*_50_, as the 50th percentile of all observed values; cells with fractions strictly less than *f*_50_ were said to have a low fraction of propagative events at the baseline, whereas cells with fractions greater than or equal to *f*_50_ were said to have a high fraction of propagative events at the baseline (Extended Data Fig. 5e, top). The fraction of astrocytes that were responders with propagative events to GABA or glutamate were separately estimated from among those astrocytes that had low baseline propagation and those that had high baseline propagation, as described in the section entitled “Determining responding cells based on static and propagative events”. Owing to the low number of cells in each split group for individual FOVs, the overall average was estimated by pooling all neighbouring astrocytes in each group across FOVs.

Similarly for each neighbouring astrocyte with baseline propagative events, we computed the rate of all events within the baseline period. Separately for GABA and glutamate, we used the baseline event rate across all neighbouring astrocytes to define the threshold baseline event rate, *r*_50_, as the 50th percentile of all observed values; cells with baseline rates strictly less than *r*_50_ were said to have low overall baseline event rates, whereas cells with fractions greater than or equal to *r*_50_ were said to have high overall baseline event rates (Extended Data Fig. 5e, bottom). The fraction of astrocytes that were responders with propagative events to GABA or glutamate were separately estimated from among those astrocytes that had low overall baseline event rates and those that had high overall baseline event rates, as above.

To determine the distribution of these responder fractions (among astrocytes with low and high fractions of propagative events at the baseline, or among astrocytes with low and high overall baseline event rates), we carried out the same procedure for estimating these fractions on surrogate data generated by hierarchically bootstrapping Ca^2+^ event data 10,000 times (as in the section entitled “Propagation probability”). Standard errors were computed as the standard deviation of these empirical distributions.

For each NT, we next sought to determine whether there were significant differences between the fraction of astrocytes that were responders with propagative events among cells within the two groupings (that is, a low versus a high fraction of propagative events at the baseline; low versus high overall baseline event rate). Separately for GABA and glutamate, for each group comparison, we computed the difference between the two responder fractions, as well as the distribution of this difference under an imposed condition in which there was no systematic difference in uncaging response between astrocytes in the two groups. To do this, we carried out the same procedure as above for estimating responder fractions in the specified groups (for example, low fraction of propagative events at the baseline and high fraction of propagative events at the baseline) as well as the difference between the two, but on surrogate data generated by permuting the group labels; this procedure was repeated 10,000 times. As it was unknown a priori which group in either comparison—low or high baseline propagation, or low or high overall baseline event rate—would have a higher fraction of responder cells, a two-sided *P* value was estimated from these surrogate values, as in equation (2).

#### Simulations to validate characteristics of responder fraction estimates (Extended Data Fig. 5k)

Stratifying propagative event responses by the fraction of propagative events in the baseline may induce regression to the mean (RTM) effects, resulting in a bias towards higher observed responsiveness in the low fraction of propagative events at the baseline group as compared to the high-fraction group. In general, observed effects in differences of repeated measurements stratified by baseline values can arise from a combination of RTM effects and real effects—with the strength of the contribution from RTM depending on the dependency structure and measurement error characteristics in the data—complicating attribution of the observed total effect. To contextualize the observed effect sizes relative to the distribution of effects produced from a pure RTM process, we carried out the same procedure as above for estimating responder fractions in the low and high fraction of propagative events at the baseline groups, but using surrogate data generated using a random point process model. This model produced simulated event data structured in the same way as the observed dataset: for each cell, the model generated two independent homogeneous Poisson processes, one corresponding to static events and the other corresponding to propagative events. During the simulated baseline period, from 60 s to 0 s pre-‘uncaging’, the rates of these two processes in each cell were set to the observed rate of the corresponding type of event during the veridical baseline period. During the simulated post-‘uncaging’ period, from 0 s to 120 s, the rates of these two processes in each cell were determined by multiplying that cell’s baseline rate for the corresponding event type by a response ratio, which was chosen from the empirical distribution of observed post-/pre-uncaging event ratios from among all neighbouring cells for the given event type. In this way, the simulation modelled the overall response characteristics for propagative events, but in a way that was decoupled from the propagative event fraction in the baseline period.

This simulation procedure was repeated 10,000 times, resulting in a distribution of low–high response fraction differences observed in surrogate data structured in the same way as either the GABA or glutamate uncaging datasets, but with no explicit dependence of cells’ propagative event responses on the baseline propagative event fraction. To summarize the observed effect relative to the effects seen in these simulations, we calculated the fraction of simulations with low–high differences larger than the observed effect.

### Reporting summary

Further information on research design is available in the Nature Portfolio Reporting Summary linked to this article.

## Online content

Any methods, additional references, Nature Portfolio reporting summaries, source data, extended data, supplementary information, acknowledgements, peer review information; details of author contributions and competing interests; and statements of data and code availability are available at 10.1038/s41586-024-07311-5.

### Supplementary information

Supplementary Tables Supplementary Tables 1–14.

Reporting Summary

Supplementary Video 1 Astrocyte Ca^2+^ response to GABA receptor activation of V1 slices. Bath-application of GABA_B_R agonist (Baclofen, 50 μM) in V1 slices expressing GCaMP6f in astrocytes increases population Ca^2+^ activity. Ca^2+^ increases are larger in response to t-ACPD (Supplementary Video 2) compared to Baclofen (Supplementary Video 1) within the same astrocyte population. (Action potentials are blocked with TTX.) Left: raw movie; right: AQuA-detected Ca^2+^ events (colors are individual events). Agonist enters the recording chamber when agonist name appears in upper right corner. The 810 × 810 µm field-of-view (FOV) encompasses most, if not all, cortical layers with pia visible at the top of the FOV. Images acquired at 1.4 Hz; playback speed 60 frames/s.

Supplementary Video 2 Astrocyte Ca^2+^ response to glutamate receptor activation of V1 slices. Bath-application of mGluR agonist (t-ACPD, 50 µM) in V1 slices expressing GCaMP6f in astrocytes increases population Ca^2+^ activity. Ca^2+^ increases are larger in response to t-ACPD (Supplementary Video 2) compared to Baclofen (Supplementary Video 1) within the same astrocyte population. (Action potentials are blocked with TTX.) Left: raw movie; right: AQuA-detected Ca^2+^ events (colors are individual events). Agonist enters the recording chamber when agonist name appears in upper right corner. The 810 × 810 µm field-of-view (FOV) encompasses most, if not all, cortical layers with pia visible at the top of the FOV. Images acquired at 1.4 Hz; playback speed 60 frames/s.

Supplementary Video 3 Single astrocyte Ca^2+^ response to local release of GABA. 2P uncaging of RuBi-GABA (300 µM) in a single GCaMP6f-expressing cell leads to prolonged astrocyte Ca^2+^ increases near the site of neurotransmitter release, and throughout the stimulated astrocyte. (Action potentials are blocked with TTX.) Left: raw movie; right: AQuA-detected Ca^2+^ events (colors are individual events). Neurotransmitter has been uncaged when white arrow pointing to uncaging site and neurotransmitter name appear. Images acquired at 1.4 Hz, with every 2 frames averaged (raw fluorescence) or summed (AQuA overlay); playback speed 15 frames/s.

Supplementary Video 4 Single astrocyte Ca^2+^ response to local release of glutamate. 2P uncaging of RuBi-glutamate (300 µM) in a single GCaMP6f-expressing cell leads to prolonged astrocyte Ca^2+^ increases near the site of neurotransmitter release, and throughout the stimulated astrocyte. (Action potentials are blocked with TTX.) Left: raw movie; right: AQuA-detected Ca^2+^ events (colors are individual events). Neurotransmitter has been uncaged when white arrow pointing to uncaging site and neurotransmitter name appear. Images acquired at 1.4 Hz, with every 2 frames averaged (raw fluorescence) or summed (AQuA overlay); playback speed 15 frames/s.

Supplementary Video 5 Network-level astrocyte Ca^2+^ response to local release of GAB 2P uncaging of RuBi-GABA (300 µM) in GCaMP6f-expressing astrocytes leads to prolonged increases in Ca^2+^ activity throughout the local astrocyte network. (Action potentials are blocked with TTX.) Raw movies are pseudo-colored with black/red representing relatively low fluorescence levels and yellow/white representing relatively high fluorescence levels. Neurotransmitter has been uncaged when white arrow pointing to uncaging site and neurotransmitter name appear. Images acquired at 1.4 Hz; playback speed 30 frames/s.

Supplementary Video 6 Network-level astrocyte Ca^2+^ response to local release of glutamate. 2P uncaging of RuBi-glutamate (300 µM) in GCaMP6f-expressing astrocytes leads to prolonged increases in Ca^2+^ activity throughout the local astrocyte network. (Action potentials are blocked with TTX.) Raw movies are pseudo-colored with black/red representing relatively low fluorescence levels and yellow/white representing relatively high fluorescence levels. Neurotransmitter has been uncaged when white arrow pointing to uncaging site and neurotransmitter name appear. Images acquired at 1.4 Hz; playback speed 30 frames/s.

Supplementary Video 7 Reduced Cx43 expression in *Cx43*^*fl/fl*^ Cre^+^ astrocytes. A 5 µm confocal z-stack of immunostained tissue showing expression of astrocytic RFP-Cre (magenta, left & right) and Cx43 (cyan, middle & right). The z-stack was acquired with a 0.25 µm z-step.

## Data Availability

All data used for this study are available in the public repository Dryad^66^ at 10.5061/dryad.83bk3jb0j. Ribosomal mRNA expression data in visual cortex astrocytes were obtained from the public database the National Center for Biotechnology Information Gene Expression Omnibus with the accession number GSE161398.

## Code availability

All analysis code used for this study is available in the public repository Zenodo^67^ at 10.5281/zenodo.10681987.

## References

[1] Poskanzer KE, Yuste R (2016). Astrocytes regulate cortical state switching in vivo. Proc. Natl Acad. Sci. USA.

[2] Mariotti L, Losi G, Sessolo M, Marcon I, Carmignoto G (2016). The inhibitory neurotransmitter GABA evokes long-lasting Ca^2+^ oscillations in cortical astrocytes. Glia.

[3] Bindocci E (2017). Three-dimensional Ca^2+^ imaging advances understanding of astrocyte biology. Science.

[4] Di Castro MA (2011). Local Ca^2+^ detection and modulation of synaptic release by astrocytes. Nat. Neurosci..

[5] Vaidyanathan TV, Collard M, Yokoyama S, Reitman ME, Poskanzer KE (2021). Cortical astrocytes independently regulate sleep depth and duration via separate GPCR pathways. eLife.

[6] Perea G, Araque A (2005). Properties of synaptically evoked astrocyte calcium signal reveal synaptic information processing by astrocytes. J. Neurosci..

[7] Perea G (2016). Activity-dependent switch of GABAergic inhibition into glutamatergic excitation in astrocyte-neuron networks. eLife.

[8] Wang Y (2019). Accurate quantification of astrocyte and neurotransmitter fluorescence dynamics for single-cell and population-level physiology. Nat. Neurosci..

[9] Paukert M (2014). Norepinephrine controls astroglial responsiveness to local circuit activity. Neuron.

[10] Nimmerjahn A, Mukamel EA, Schnitzer MJ (2009). Motor behavior activates Bergmann glial networks. Neuron.

[11] Reitman ME (2023). Norepinephrine links astrocytic activity to regulation of cortical state. Nat. Neurosci..

[12] Mu Y (2019). Glia accumulate evidence that actions are futile and suppress unsuccessful behavior. Cell.

[13] Ma Z, Stork T, Bergles DE, Freeman MR (2016). Neuromodulators signal through astrocytes to alter neural circuit activity and behaviour. Nature.

[14] Katz M (2019). Glutamate spillover in *C. elegans* triggers repetitive behavior through presynaptic activation of MGL-2/mGluR5. Nat. Commun..

[15] Gordon GRJ (2009). Astrocyte-mediated distributed plasticity at hypothalamic glutamate synapses. Neuron.

[16] Poskanzer KE, Yuste R (2011). Astrocytic regulation of cortical UP states. Proc. Natl Acad. Sci. USA.

[17] Kang J, Jiang L, Goldman SA, Nedergaard M (1998). Astrocyte-mediated potentiation of inhibitory synaptic transmission. Nat. Neurosci..

[18] Farhy-Tselnicker I (2021). Activity-dependent modulation of synapse-regulating genes in astrocytes. eLife.

[19] Srinivasan R (2016). New transgenic mouse lines for selectively targeting astrocytes and studying calcium signals in astrocyte processes in situ and in vivo. Neuron.

[20] Serrano A, Haddjeri N, Lacaille J-C, Robitaille R (2006). GABAergic network activation of glial cells underlies hippocampal heterosynaptic depression. J. Neurosci..

[21] Tang W (2015). Stimulation-evoked Ca^2+^ signals in astrocytic processes at hippocampal CA3-CA1 synapses of adult mice are modulated by glutamate and ATP. J. Neurosci..

[22] Sun W (2013). Glutamate-dependent neuroglial calcium signaling differs between young and adult brain. Science.

[23] Harada K (2017). Red fluorescent protein-based cAMP indicator applicable to optogenetics and in vivo imaging. Sci. Rep..

[24] Moldrich RX, Apricó K, Diwakarla S, O’Shea RD, Beart PM (2002). Astrocyte mGlu_2/3_-mediated cAMP potentiation is calcium sensitive: studies in murine neuronal and astrocyte cultures. Neuropharmacology.

[25] Oe Y (2020). Distinct temporal integration of noradrenaline signaling by astrocytic second messengers during vigilance. Nat. Commun..

[26] Kellner V (2021). Dual metabotropic glutamate receptor signaling enables coordination of astrocyte and neuron activity in developing sensory domains. Neuron.

[27] Hirono M, Yoshioka T, Konishi S (2001). GABA_B_ receptor activation enhances mGluR-mediated responses at cerebellar excitatory synapses. Nat. Neurosci..

[28] New DC, An H, Ip NY, Wong YH (2006). GABA_B_ heterodimeric receptors promote Ca^2+^ influx via store-operated channels in rat cortical neurons and transfected Chinese hamster ovary cells. Neuroscience.

[29] Zeng W (2003). A new mode of Ca^2+^ signaling by G protein-coupled receptors: gating of IP_3_ receptor Ca^2+^ release channels by Gβγ. Curr. Biol..

[30] Durkee CA (2019). G_i/o_ protein-coupled receptors inhibit neurons but activate astrocytes and stimulate gliotransmission. Glia.

[31] Araya R, Vogels TP, Yuste R (2014). Activity-dependent dendritic spine neck changes are correlated with synaptic strength. Proc. Natl Acad. Sci. USA.

[32] Bernardinelli Y (2014). Activity-dependent structural plasticity of perisynaptic astrocytic domains promotes excitatory synapse stability. Curr. Biol..

[33] Filevich O, Etchenique R (2013). RuBiGABA-2: a hydrophilic caged GABA with long wavelength sensitivity. Photochem. Photobiol. Sci..

[34] Fino E (2009). RuBi-glutamate: two-photon and visible-light photoactivation of neurons and dendritic spines. Front. Neural Circuits.

[35] Panatier A (2011). Astrocytes are endogenous regulators of basal transmission at central synapses. Cell.

[36] Zheng K (2015). Time-resolved imaging reveals heterogeneous landscapes of nanomolar Ca^2+^ in neurons and astroglia. Neuron.

[37] Marvin JS (2013). An optimized fluorescent probe for visualizing glutamate neurotransmission. Nat. Methods.

[38] Rouach N, Koulakoff A, Abudara V, Willecke K, Giaume C (2008). Astroglial metabolic networks sustain hippocampal synaptic transmission. Science.

[39] Papouin T, Dunphy JM, Tolman M, Dineley KT, Haydon PG (2017). Septal cholinergic neuromodulation tunes the astrocyte-dependent gating of hippocampal NMDA receptors to wakefulness. Neuron.

[40] Chever O, Dossi E, Pannasch U, Derangeon M, Rouach N (2016). Astroglial networks promote neuronal coordination. Sci. Signal..

[41] Han Y (2014). Astrocyte-restricted disruption of connexin-43 impairs neuronal plasticity in mouse barrel cortex. Eur. J. Neurosci..

[42] Ding F (2013). α1-Adrenergic receptors mediate coordinated Ca^2+^ signaling of cortical astrocytes in awake, behaving mice. Cell Calcium.

[43] King CM (2020). Local resting Ca^2+^ controls the scale of astroglial Ca^2+^ signals. Cell Rep..

[44] Rupprecht, P. et al. Centripetal integration of past events by hippocampal astrocytes and its regulation by the locus coeruleus. Preprint at *bioRxiv*10.1101/2022.08.16.504030 (2023).

[45] Gerstner W, Lehmann M, Liakoni V, Corneil D, Brea J (2018). Eligibility traces and plasticity on behavioral time scales: experimental support of neoHebbian three-factor learning rules. Front. Neural Circuits.

[46] Goldman, M. S., Compte, A. & Wang, X. J. in *Encyclopedia of Neuroscience* 165–178 (Elsevier, 2009).

[47] Deemyad T, Lüthi J, Spruston N (2018). Astrocytes integrate and drive action potential firing in inhibitory subnetworks. Nat. Commun..

[48] Doron A (2022). Hippocampal astrocytes encode reward location. Nature.

[49] Arizono M (2020). Structural basis of astrocytic Ca^2+^ signals at tripartite synapses. Nat. Commun..

[50] Brunskine C, Passlick S, Henneberger C (2022). Structural heterogeneity of the gabaergic tripartite synapse. Cells.

[51] Michaluk P, Heller JP, Rusakov DA (2021). Rapid recycling of glutamate transporters on the astroglial surface. eLife.

[52] Batiuk MY (2020). Identification of region-specific astrocyte subtypes at single cell resolution. Nat. Commun..

[53] Bayraktar OA (2020). Astrocyte layers in the mammalian cerebral cortex revealed by a single-cell in situ transcriptomic map. Nat. Neurosci..

[54] Toyofuku T (1998). Intercellular calcium signaling via gap junction in connexin-43-transfected cells. J. Biol. Chem..

[55] Khodakhah K, Ogden D (1993). Functional heterogeneity of calcium release by inositol trisphosphate in single Purkinje neurones, cultured cerebellar astrocytes, and peripheral tissues. Proc. Natl Acad. Sci. USA.

[56] Wiencken-Barger AE, Djukic B, Casper KB, McCarthy KD (2007). A role for Connexin43 during neurodevelopment. Glia.

[57] Yang Y (2011). Molecular comparison of GLT1^+^ and ALDH1L1^+^ astrocytes in vivo in astroglial reporter mice. Glia.

[58] Wang Y (2020). SynQuant: an automatic tool to quantify synapses from microscopy images. Bioinformatics.

[59] Dubbs A, Guevara J, Yuste R (2016). moco: fast motion correction for calcium imaging. Front. Neuroinformatics.

[60] Fedchyshyn MJ, Wang L-Y (2007). Activity-dependent changes in temporal components of neurotransmission at the juvenile mouse calyx of Held synapse. J. Physiol..

[61] Martínez-Cagigal, V. Multiple Testing Toolbox, 1.1.0. *MATLAB Central File Exchange* https://www.mathworks.com/matlabcentral/fileexchange/70604-multiple-testing-toolbox (2021).

[62] Groppe, D. fdr_bh, 2.3.0.0. *MATLAB Central File Exchange* https://www.mathworks.com/matlabcentral/fileexchange/27418-fdr_bh (2010).

[63] Phipson, B. & Smyth, G. K. Permutation P-values should never be zero: calculating exact P-values when permutations are randomly drawn. *Stat. Appl. Genet. Mol. Biol.*10.2202/1544-6115.1585 (2010). 10.2202/1544-6115.1585 21044043

[64] Seabold, S. & Perktold, J. Statsmodels: econometric and statistical modeling with Python. In *Proc. 9th**Python in Science Conference* 92–96 (SciPy, 2010).

[65] Virtanen P (2020). SciPy 1.0: fundamental algorithms for scientific computing in Python. Nat. Methods.

[66] Cahill, M. et al. Network-level encoding of local neurotransmitters in cortical astrocytes. *Dryad*10.5061/dryad.83bk3jb0j (2024). 10.1038/s41586-024-07311-5 PMC11062919 38632406

[67] Poskanzer, K. Network-level encoding of local neurotransmitters in cortical astrocytes. Network-level encoding of local neurotransmitters in cortical astrocytes. *Zenodo*10.5281/zenodo.10681987 (2024).

[68] Kingston AE (1998). LY341495 is a nanomolar potent and selective antagonist of group II metabotropic glutamate receptors. Neuropharmacology.

