# Publisher Correction: Network-level encoding of local neurotransmitters in cortical astrocytes

**DOI:** 10.1038/s41586-024-07468-z

**Published:** 2024-05-13

**Authors:** Michelle K. Cahill, Max Collard, Vincent Tse, Michael E. Reitman, Roberto Etchenique, Christoph Kirst, Kira E. Poskanzer

**Affiliations:** 1 Department of Biochemistry & Biophysics, University of California, San Francisco, CA USA; 2 Neuroscience Graduate Program, University of California, San Francisco, CA USA; 3 Departamento de Química Inorgánica, Analítica y Química Física, INQUIMAE, Facultad de Ciencias Exactas y Naturales, Universidad de Buenos Aires, CONICET, Buenos Aires, Argentina; 4 Department of Anatomy, University of California, San Francisco, CA USA; 5 Kavli Institute for Fundamental Neuroscience, San Francisco, CA USA; 6 Lawrence Berkeley National Laboratory, Berkeley, CA USA

**Keywords:** Astrocyte, Cellular neuroscience

Correction to: *Nature* 10.1038/s41586-024-07311-5 Published online 17 April 2024

In the version of this article initially published, an erroneous grey hexagon appeared next to the bottom label in Fig. 3a. It has now been removed from the HTML and PDF versions of the article.

