# Supplementary material for: Network-level encoding of local neurotransmitters in cortical astrocytes
Source: Nature. 2024 Apr 17;629(8010):146–53. doi: 10.1038/s41586-024-07311-5 (PMC11062919; doi:10.1038/s41586-024-07311-5)
Supplement: Supplementary file 1 — Supplementary Tables 1–14. [file 41586_2024_7311_MOESM1_ESM.docx]

**Supplementary Table 1.**

| **Condition** | **Time relative to agonist entry (s)** | ***p-*values (raw)** | ***p*-values (adjusted)** |
| --- | --- | --- | --- |
| **Baclofen** | **-240 ­­– -180** | 0.598040195980402 | 1.90331632015370 |
|  | **-180 – -120** | 0.429157084291571 | 1.82110765113965 |
|  | **-120 – -60** | 0.374462553744626 | 1.82110765113965 |
|  | **-60 – 0** | 0.570242975702430 | 1.90331632015370 |
|  | **0–60** | 0.339466053394661 | 1.82110765113965 |
|  | **60–120** | 9.99900009999000e-05 | **0.000848605615628913** |
|  | **120–180** | 9.99900009999000e-05 | **0.000848605615628913** |
|  | **180–240** | 9.99900009999000e-05 | **0.000848605615628913** |
| **t-ACPD** | **-240 ­­– -180** | 0.950704929507049 | 2.80096426865250 |
|  | **-180 – -120** | 0.939206079392061 | 2.80096426865250 |
|  | **-120 – -60** | 0.851514848515149 | 2.80096426865250 |
|  | **-60 – 0** | 0.990100989901010 | 2.80096426865250 |
|  | **0–60** | 9.99900009999000e-05 | **0.000848605615628913** |
|  | **60–120** | 9.99900009999000e-05 | **0.000848605615628913** |
|  | **120–180** | 9.99900009999000e-05 | **0.000848605615628913** |
|  | **180–240** | 0.0241975802419758 | 0.154021919236648 |

**Statistics for Fig. 1c: Change in event frequency in astrocytes during bath-application of agonist.** Permutation testing used to identify time-points with changes in event frequency greater than chance for each agonist. *p*-values corrected for multiple comparisons using Benjamini-Yekutieli procedure with FDR ≤ 0.05. Adjusted *p*-values < 0.05 are bold.

**Supplementary Table 2.**

| **Feature** | **Agonist concentration (μM)** | ***p-*values (raw)** | ***p*-values (adjusted)** |
| --- | --- | --- | --- |
| **Percent field active** | 5 | 0.106884262919877 | 0.106884262919877 |
|  | 25 | 0.00453636186896923 | **0.00907272373793845** |
|  | 50 | 0.000175165474580620 | **0.000700661898322481** |
|  | 100 | 0.000644835771994738 | **0.00193450731598421** |
| **Event area** | 5 | 0.134291909278406 | 0.134291909278406 |
|  | 25 | 0.00336246922231183 | **0.0100874076669355** |
|  | 50 | 0.00274661524985296 | **0.0109864609994119** |
|  | 100 | 0.0355015432722231 | 0.0710030865444463 |
| **Event duration** | 5 | 0.794523834669017 | 0.794523834669017 |
|  | 25 | 0.00693357100768626 | **0.0208007130230588** |
|  | 50 | 0.000707221042301198 | **0.00282888416920479** |
|  | 100 | 0.0222444305319271 | **0.0444888610638542** |
| **Event propagation** | 5 | 0.503037904758348 | 0.503037904758348 |
|  | 25 | 0.00201639415469164 | **0.00806557661876658** |
|  | 50 | 0.00332572662050501 | **0.00997717986151502** |
|  | 100 | 0.0102174453241840 | **0.0204348906483680** |

**Statistics for Fig. 1e–h: Dose-response curves.** Paired t-tests at each concentration compare response to each agonist for each feature. *p*-values corrected for multiple comparisons using Bonferroni-Holm correction with FWER ≤ 0.05. Adjusted *p*-values < 0.05 are bold.

**Supplementary Table 3.**

| **Condition** | **Time relative to agonist entry (s)** | ***p-*values (raw)** | ***p*-values (adjusted)** |
| --- | --- | --- | --- |
| **Baclofen** | **-300 – -270** | 0.950904909509049 | 3.48427432652907 |
|  | **-270 – -240** | 0.617338266173383 | 3.48427432652907 |
|  | **-240 – -210** | 0.853814618538146 | 3.48427432652907 |
|  | **-210 – -180** | 0.391760823917608 | 3.08080444588937 |
|  | **-180 – -150** | 0.996900309969003 | 3.48427432652907 |
|  | **-150 – -120** | 0.528947105289471 | 3.48427432652907 |
|  | **-120 – -90** | 0.957004299570043 | 3.48427432652907 |
|  | **-90 – -60** | 0.982301769823018 | 3.48427432652907 |
|  | **-60 – -30** | 0.995700429957004 | 3.48427432652907 |
|  | **-30–0** | 0.654434556544346 | 3.48427432652907 |
|  | **0–30** | 0.994300569943006 | 3.48427432652907 |
|  | **30–60** | 0.00129987001299870 | **0.0157264137105124** |
|  | **60–90** | 9.99900009999000e-05 | **0.00314528274210247** |
|  | **90–120** | 0.00149985001499850 | **0.0157264137105124** |
|  | **120–150** | 9.99900009999000e-05 | **0.00314528274210247** |
|  | **150–180** | 0.0198980101989801 | 0.178831790193826 |
|  | **180–210** | 0.000499950004999500 | **0.00786320685525618** |
|  | **210–240** | 0.000199980001999800 | **0.00419371032280330** |
| **t-ACPD** | **-300 – -270** | 0.925507449255074 | 3.49510807819631 |
|  | **-270 – -240** | 0.923607639236076 | 3.49510807819631 |
|  | **-240 – -210** | 0.895510448955105 | 3.49510807819631 |
|  | **-210 – -180** | 0.886511348865114 | 3.49510807819631 |
|  | **-180 – -150** | 0.888211178882112 | 3.49510807819631 |
|  | **-150 – -120** | 0.865413458654135 | 3.49510807819631 |
|  | **-120 – -90** | 0.952504749525048 | 3.49510807819631 |
|  | **-90 – -60** | 0.707029297070293 | 3.49510807819631 |
|  | **-60 – -30** | 0.919608039196080 | 3.49510807819631 |
|  | **-30–0** | 0.905409459054095 | 3.49510807819631 |
|  | **0–30** | 9.99900009999000e-05 | **0.00314528274210247** |
|  | **30–60** | 9.99900009999000e-05 | **0.00314528274210247** |
|  | **60–90** | 1 | 3.49510807819631 |
|  | **90–120** | 1 | 3.49510807819631 |
|  | **120–150** | 1 | 3.49510807819631 |
|  | **150–180** | 1 | 3.49510807819631 |
|  | **180–210** | 1 | 3.49510807819631 |
|  | **210–240** | 1 | 3.49510807819631 |

**Statistics for Extended Data Fig. 1d: Mean ∆F/F in astrocytes during bath-application of agonist.** Permutation testing used to identify time-points with increases in ∆F/F greater than chance for each agonist. *p*-values corrected for multiple comparisons using Benjamini-Yekutieli procedure with FDR ≤ 0.05. Adjusted *p*-values < 0.05 are bold.

**Supplementary Table 4.**

| **Feature** | **Test** | **Comparison** | ***p-*values** |
| --- | --- | --- | --- |
| **Area** | 1-way ANOVA |  | **2.64502036644004e-13** |
|  | Tukey-Kramer Test | Bac_pre v Bac_post | 0.898277274752255 |
|  |  | Bac_pre v tACPD_pre | 0.995109946477354 |
|  |  | Bac_pre v tACPD_post | 7.59031018511269e-05 |
|  |  | Bac_post v tACPD_pre | 0.975557200328527 |
|  |  | Bac_post v tACPD_post | **8.83569961640518e-09** |
|  |  | tACPD_pre v tACPD_post | **0.000158328341554381** |
| **Duration** | 1-way ANOVA |  | **2.90200679997044e-19** |
|  | Tukey-Kramer Test | Bac_pre v Bac_post | 0.705179159520994 |
|  |  | Bac_pre v tACPD_pre | 0.997414358965489 |
|  |  | Bac_pre v tACPD_post | 0.000778858034344410 |
|  |  | Bac_post v tACPD_pre | 0.822905967024531 |
|  |  | Bac_post v tACPD_post | **3.76825814996096e-09** |
|  |  | tACPD_pre v tACPD_post | **0.000133775981740025** |
| **Propagation distance** | 1-way ANOVA |  | **5.06378633615887e-15** |
|  | Tukey-Kramer Test | Bac_pre v Bac_post | 0.913414390535003 |
|  |  | Bac_pre v tACPD_pre | 0.998952459006666 |
|  |  | Bac_pre v tACPD_post | 3.10437553621146e-05 |
|  |  | Bac_post v tACPD_pre | 0.959725706670736 |
|  |  | Bac_post v tACPD_post | **4.12490908097141e-09** |
|  |  | tACPD_pre v tACPD_post | **3.44219605847851e-05** |

**Statistics for Extended Data Fig. 1g: Event features pre- and post-agonist addition**. Comparison of distributions of event area, duration, and propagation 120–0s before (“Pre”) or 0–120s after (“Post”) addition of baclofen (50 μM) or t-ACPD (50 μM). One-way ANOVA followed by Tukey-Kramer Test determine significant pairwise comparisons between conditions. *p*-values < 0.05 are bold, greyed-out cells are pairwise comparisons that are not relevant.

**Supplementary Table 5.**

| **Condition** | **Time relative to uncaging (s)** | ***p-*values (raw)** | ***p*-values (adjusted)** |
| --- | --- | --- | --- |
| **GABA** | 0–30 | 0.196880311968803 | 0.449543378995434 |
|  | 30–60 | 0.149385061493851 | 0.426369863013699 |
|  | 60–90 | 0.0115988401159884 | **0.0441400304414003** |
|  | 90–120 | 9.99900009999000e-05 | **0.00114155251141553** |
|  | 120–150 | 0.00229977002299770 | **0.0131278538812785** |
| **Glutamate** | 0–30 | 0.118788121187881 | 0.339041095890411 |
|  | 30–60 | 0.153984601539846 | 0.351598173515982 |
|  | 60–90 | 0.000399960003999600 | **0.00228310502283105** |
|  | 90–120 | 9.99900009999000e-05 | **0.00114155251141553** |
|  | 120–150 | 0.0591940805919408 | 0.225266362252664 |
| **GABA + CGP55845** | 0–30 | 0.237476252374763 | 0.906392694063927 |
|  | 30–60 | 0.363263673632637 | 1.03681506849315 |
|  | 60–90 | 0.238176182381762 | 0.906392694063927 |
|  | 90–120 | 0.00279972002799720 | **0.0319634703196347** |
|  | 120–150 | 0.927407259274073 | 2.11757990867580 |
| **Glutamate + LY341495** | 0–30 | 0.0266973302669733 | 0.304794520547945 |
|  | 30–60 | 0.883111688831117 | 2.23515981735160 |
|  | 60–90 | 0.978902109789021 | 2.23515981735160 |
|  | 90–120 | 0.919108089191081 | 2.23515981735160 |
|  | 120–150 | 0.650334966503350 | 2.23515981735160 |
| **Laser uncaging control** | 0–30 | 0.0105989401059894 | 0.121004566210046 |
|  | 30–60 | 0.0492950704929507 | 0.187595129375951 |
|  | 60–90 | 0.407059294070593 | 1.16181506849315 |
|  | 90–120 | 0.0379962003799620 | 0.187595129375951 |
|  | 120–150 | 0.622337766223378 | 1.42100456621005 |

**Statistics for Extended Data Fig. 2b: Change in event frequency in astrocytes directly stimulated with NT.** Permutation testing used to identify time-points with changes in event frequency greater than chance for each condition. *p*-values corrected for multiple comparisons using Benjamini-Yekutieli procedure with FDR ≤ 0.05. Adjusted *p*-values < 0.05 are bold.

**Supplementary Table 6.**

| **NT** | **Distance from uncaging (µm)** | **Time relative to uncaging (s)** | ***p-*values (raw)** | ***p*-values (adjusted)** |
| --- | --- | --- | --- | --- |
| **GABA** | **25–75** | 0–30 | 0.0569943005699430 | 0.216894977168950 |
|  |  | 30–60 | 0.798720127987201 | 1.82374429223744 |
|  |  | 60–90 | 0.0429957004299570 | 0.216894977168950 |
|  |  | 90–120 | 0.00599940005999400 | 0.0684931506849315 |
|  |  | 120–150 | 0.535546445355465 | 1.52853881278539 |
|  | **75–125** | 0–30 | 0.0666933306669333 | 0.276636225266362 |
|  |  | 30–60 | 0.187281271872813 | 0.534531963470320 |
|  |  | 60–90 | 0.0726927307269273 | 0.276636225266362 |
|  |  | 90–120 | 0.00299970002999700 | **0.0342465753424658** |
|  |  | 120–150 | 0.307269273072693 | 0.701598173515982 |
|  | **125–175** | 0–30 | 0.0418958104189581 | 0.0956621004566210 |
|  |  | 30–60 | 0.0140985901409859 | 0.0536529680365297 |
|  |  | 60–90 | 0.0192980701929807 | 0.0550799086757991 |
|  |  | 90–120 | 9.99900009999000e-05 | **0.00114155251141553** |
|  |  | 120–150 | 0.00379962003799620 | **0.0216894977168950** |
| **glutamate** | **25–75** | 0–30 | 0.00169983001699830 | **0.00646879756468798** |
|  |  | 30–60 | 0.00119988001199880 | **0.00646879756468798** |
|  |  | 60–90 | 0.00799920007999200 | **0.0228310502283105** |
|  |  | 90–120 | 9.99900009999000e-05 | **0.00114155251141553** |
|  |  | 120–150 | 0.0128987101289871 | **0.0294520547945206** |
|  | **75–125** | 0–30 | 0.255074492550745 | 0.728025114155251 |
|  |  | 30–60 | 0.00459954004599540 | **0.0262557077625571** |
|  |  | 60–90 | 0.00779922007799220 | **0.0296803652968037** |
|  |  | 90–120 | 9.99900009999000e-05 | **0.00114155251141553** |
|  |  | 120–150 | 0.576442355764424 | 1.31621004566210 |
|  | **125–175** | 0–30 | 0.113988601139886 | 0.260273972602740 |
|  |  | 30–60 | 0.0355964403559644 | 0.135464231354642 |
|  |  | 60–90 | 0.00949905009499050 | 0.0542237442922375 |
|  |  | 90–120 | 9.99900009999000e-05 | **0.00114155251141553** |
|  |  | 120–150 | 0.0554944505549445 | 0.158390410958904 |

**Statistics for Fig. 3h: Change in event frequency in the astrocyte network via Sholl-like analysis.** Permutation testing used to identify time-points with changes in event frequency greater than chance for each distance band and NT. *p*-values corrected for multiple comparisons using Benjamini-Yekutieli procedure with FDR ≤ 0.05. Adjusted *p*-values < 0.05 are bold.

**Supplementary Table 7.**

| **Condition** | **Time relative to uncaging (s)** | ***p-*values (raw)** | ***p*-values (adjusted)** |
| --- | --- | --- | --- |
| **GABA WT** | 0–30 | 0.00409959004099590 | **0.0156012176560122** |
|  | 30–60 | 0.0294970502949705 | 0.0841894977168950 |
|  | 60–90 | 0.000299970002999700 | **0.00171232876712329** |
|  | 90–120 | 9.99900009999000e-05 | **0.00114155251141553** |
|  | 120–150 | 0.519848015198480 | 1.18698630136986 |
| **Glutamate WT** | 0–30 | 0.00479952004799520 | **0.0136986301369863** |
|  | 30–60 | 9.99900009999000e-05 | **0.000570776255707763** |
|  | 60–90 | 0.000299970002999700 | **0.00114155251141553** |
|  | 90–120 | 9.99900009999000e-05 | **0.000570776255707763** |
|  | 120–150 | 0.0480951904809519 | 0.109817351598174 |
| **GABA Cx43^floxed^** | 0–30 | 0.0586941305869413 | 0.335045662100457 |
|  | 30–60 | 0.943105689431057 | 2.15342465753425 |
|  | 60–90 | 0.373262673732627 | 1.42047184170472 |
|  | 90–120 | 0.000499950004999500 | **0.00570776255707763** |
|  | 120–150 | 0.814718528147185 | 2.15342465753425 |
| **Glutamate Cx43^floxed^** | 0–30 | 0.668933106689331 | 1.90924657534247 |
|  | 30–60 | 0.878712128787121 | 2.00639269406393 |
|  | 60–90 | 0.0154984501549845 | 0.176940639269406 |
|  | 90–120 | 0.0617938206179382 | 0.352739726027397 |
|  | 120–150 | 0.666933306669333 | 1.90924657534247 |
| **GABA + CGP55845** | 0–30 | 0.229877012298770 | 1.32363013698630 |
|  | 30–60 | 0.460253974602540 | 1.32363013698630 |
|  | 60–90 | 0.234976502349765 | 1.32363013698630 |
|  | 90–120 | 0.463753624637536 | 1.32363013698630 |
|  | 120–150 | 0.971902809719028 | 2.21917808219178 |
| **Glutamate + LY341495** | 0–30 | 0.181181881811819 | 0.997716894977169 |
|  | 30–60 | 0.427057294270573 | 1.21889269406393 |
|  | 60–90 | 0.160683931606839 | 0.997716894977169 |
|  | 90–120 | 0.660733926607339 | 1.50867579908676 |
|  | 120–150 | 0.262173782621738 | 0.997716894977169 |
| **Laser uncaging control** | 0–30 | 0.00899910008999100 | 0.102739726027397 |
|  | 30–60 | 0.498050194980502 | 1.42151826484018 |
|  | 60–90 | 0.668633136686331 | 1.52671232876712 |
|  | 90–120 | 0.130786921307869 | 0.746575342465753 |
|  | 120–150 | 0.239876012398760 | 0.912861491628615 |

**Statistics for Extended Data Fig. 3g,h: Change in event frequency in individual astrocytes in the network.** Permutation testing used to identify time-points with changes in event frequency greater than chance for each condition. *p*-values corrected for multiple comparisons using Benjamini-Yekutieli procedure with FDR ≤ 0.05. Adjusted *p*-values < 0.05 are bold.

**Supplementary Table 8.**

| **NT** | **Time relative to uncaging** | ***p*-value** | ***q*-value** |
| --- | --- | --- | --- |
| **GABA** | **-60–0** | 1.0 | — |
|  | **0–30** | 0.10908909109089 | 0.218178182181781 |
|  | **30–60** | 0.132586741325867 | 0.219406630765494 |
|  | **60–90** | 0.350264973502649 | 0.389183303891833 |
|  | **90–120** | 0.276672332766723 | 0.345840415958404 |
|  | **120–150** | 0.153584641535846 | 0.219406630765494 |
| **glutamate** | **-60–0** | 1.0 | — |
|  | **0–30** | 0.0011998800119988 | **0.005999400059994** |
|  | **30–60** | 0.0161983801619838 | **0.0404959504049595** |
|  | **60–90** | 0.002999700029997 | **0.00999900009999** |
|  | **90–120** | 0.0008999100089991 | **0.005999400059994** |
|  | **120–150** | 0.651534846515348 | 0.651534846515348 |

**Statistics for Fig. 4b: Change in probability of Ca^2+^ events propagating toward or away from pia compared to baseline.** Two-sided permutation testing was used to identify time bins with changes in propagative event probability compared to baseline. *p*-values were adjusted across tested time bins using the Benjamini-Hochberg procedure to obtain *q*-values. *q*-values < 0.05 are bold.

**Supplementary Table 9.**

|  |  |  | **N** | | | | |
| --- | --- | --- | --- | --- | --- | --- | --- |
| **Genotype** | **Event Category** | **NT** | **event** | **cell** | **FOV** | **slice** | **mice** |
| **WT** | **static** | **GABA** | 8417 | 142 | 28 | 7 | 4 |
|  |  | **glutamate** | 6998 | 120 | 27 | 7 | 4 |
|  | **propagative** | **GABA** | 1358 | 135 | 28 | 7 | 4 |
|  |  | **glutamate** | 1112 | 115 | 27 | 7 | 4 |
| **Cx43^floxed^** | **static** | **GABA** | 1566 | 60 | 28 | 14 | 8 |
|  |  | **glutamate** | 1215 | 47 | 23 | 14 | 8 |
|  | **propagative** | **GABA** | 487 | 57 | 28 | 14 | 8 |
|  |  | **glutamate** | 348 | 47 | 23 | 14 | 8 |

**N for Fig. 4f, h, j, k and Extended Data Fig. 6: Fold-change in rate of static or propagative Ca^2+^ events in neighboring cells post NT-uncaging in WT and Cx43^floxed^ mice.** Event rate changes were used to calculate the fraction of neighboring cells/FOV responding to NT-uncaging for each condition (Fig. 4 h and k and Extended Data Fig. 6 e–f).

**Supplementary Table 10.**

| **Genotype** | **Event Category** | **NT** | **Time relative to uncaging** | ***p*-value** | ***q*-value** |
| --- | --- | --- | --- | --- | --- |
| **WT** | **static** | **GABA** | **-60–0** | 1.0 | — |
|  |  |  | **0–30** | 0.5649435056494350 | 0.5888411158884110 |
|  |  |  | **30–60** | 0.5888411158884110 | 0.5888411158884110 |
|  |  |  | **60–90** | 0.0560943905609439 | 0.1121887811218870 |
|  |  |  | **90–120** | 0.0012998700129987 | **0.0064993500649935** |
|  |  |  | **120–150** | 0.0150984901509849 | **0.0377462253774622** |
|  |  | **glutamate** | **-60–0** | 1.0 | — |
|  |  |  | **0–30** | 0.2686731326867310 | 0.3735876412358760 |
|  |  |  | **30–60** | 0.0066993300669933 | **0.0223311002233110** |
|  |  |  | **60–90** | 0.1374862513748620 | 0.2291437522914370 |
|  |  |  | **90–120** | 0.0009999000099990 | **0.0064993500649935** |
|  |  |  | **120–150** | 0.2988701129887010 | 0.3735876412358760 |
|  | **propagative** | **GABA** | **-60–0** | 1.0 | — |
|  |  |  | **0–30** | 0.0341965803419658 | 0.0683931606839316 |
|  |  |  | **30–60** | 0.6876312368763120 | 0.7640347076403470 |
|  |  |  | **60–90** | 0.2723727627237270 | 0.3404659534046590 |
|  |  |  | **90–120** | 0.0726927307269273 | 0.1211545512115450 |
|  |  |  | **120–150** | 0.9524047595240470 | 0.9524047595240470 |
|  |  | **glutamate** | **-60–0** | 1.0 | — |
|  |  |  | **0–30** | 0.0071992800719928 | **0.0179982001799820** |
|  |  |  | **30–60** | 0.0071992800719928 | **0.0179982001799820** |
|  |  |  | **60–90** | 0.0000999900009999 | **0.0004999500049995** |
|  |  |  | **90–120** | 0.0000999900009999 | **0.0004999500049995** |
|  |  |  | **120–150** | 0.2249775022497750 | 0.3213964317853920 |
| **Cx43^floxed^** | **static** | **GABA** | **-60–0** | 1.0 | — |
|  |  |  | **0–30** | 0.961603839616038 | 0.995600439956004 |
|  |  |  | **30–60** | 0.698030196980302 | 0.995600439956004 |
|  |  |  | **60–90** | 0.859614038596140 | 0.995600439956004 |
|  |  |  | **90–120** | 0.198480151984801 | 0.995600439956004 |
|  |  |  | **120–150** | 0.874012598740125 | 0.995600439956004 |
|  |  | **glutamate** | **-60–0** | 1.0 | — |
|  |  |  | **0–30** | 0.559344065593440 | 0.995600439956004 |
|  |  |  | **30–60** | 0.995600439956004 | 0.995600439956004 |
|  |  |  | **60–90** | 0.915308469153084 | 0.995600439956004 |
|  |  |  | **90–120** | 0.700929907009299 | 0.995600439956004 |
|  |  |  | **120–150** | 0.798820117988201 | 0.995600439956004 |
|  | **propagative** | **GABA** | **-60–0** | 1.0 | — |
|  |  |  | **0–30** | 0.812918708129187 | 0.933684409336844 |
|  |  |  | **30–60** | 0.939206079392060 | 0.939206079392060 |
|  |  |  | **60–90** | 0.444755524447555 | 0.933684409336844 |
|  |  |  | **90–120** | 0.830316968303169 | 0.933684409336844 |
|  |  |  | **120–150** | 0.813818618138186 | 0.933684409336844 |
|  |  | **glutamate** | **-60–0** | 1.0 | — |
|  |  |  | **0–30** | 0.793020697930207 | 0.933684409336844 |
|  |  |  | **30–60** | 0.834616538346165 | 0.933684409336844 |
|  |  |  | **60–90** | 0.397960203979602 | 0.933684409336844 |
|  |  |  | **90–120** | 0.379562043795620 | 0.933684409336844 |
|  |  |  | **120–150** | 0.840315968403159 | 0.933684409336844 |

**Statistics for Fig. 4f, j and Extended Data Fig. 6 b–c: Fold-change in rate of static or propagative Ca^2+^ events among neighboring cells post NT-uncaging in WT and Cx43^floxed^ mice.** One-sided permutation test used to identify time bins with static or propagative event rate increases compared to baseline. *p*-values were adjusted across tested time bins using the Benjamini-Hochberg procedure to obtain *q*-values. *q*-values < 0.05 are bold.

**Supplementary Table 11.**

|  |  | **N** | | | | |
| --- | --- | --- | --- | --- | --- | --- |
| **Event Category** | **NT** | **event** | **cell** | **FOV** | **slice** | **mice** |
| **all** | **GABA** | 9775 | 142 | 28 | 7 | 4 |
|  | **glutamate** | 8110 | 120 | 27 | 7 | 4 |
| **propagative** | **GABA** | 1358 | 135 | 28 | 7 | 4 |
|  | **glutamate** | 1112 | 115 | 27 | 7 | 4 |

**N for Fig. 4m: Fraction of neighboring cells responding to NT-uncaging with propagative event frequency increases separated by baseline activity levels.** “All” (static and propagative) events were used to calculate overall baseline event rate and fraction of propagative events in the baseline period to separate cells into “low” and “high” overall baseline activity or “low” and “high” baseline propagation, respectively. Propagative events were used to categorize cells as “responders” or “non-responders” to NT-uncaging.

**Supplementary Table 12.**

| **Event Feature** | **NT** | **Time relative to uncaging** | ***p*-value** | ***q*-value** |
| --- | --- | --- | --- | --- |
| **Area** | **GABA** | **-60–0** | 1.0 | — |
|  |  | **0–30** | 0.2707292707292700 | 0.3867561010418150 |
|  |  | **30–60** | 0.7262737262737260 | 0.8069708069708070 |
|  |  | **60–90** | 0.5004995004995000 | 0.6256243756243750 |
|  |  | **90–120** | 0.1428571428571420 | 0.2380952380952380 |
|  |  | **120–150** | 0.0159840159840159 | **0.0474525474525474** |
|  | **glutamate** | **-60–0** | 1.0 | — |
|  |  | **0–30** | 0.0099900099900100 | **0.0474525474525474** |
|  |  | **30–60** | 0.0709290709290709 | 0.1418581418581410 |
|  |  | **60–90** | 0.0189810189810189 | **0.0474525474525474** |
|  |  | **90–120** | 0.0019980019980020 | **0.0199800199800199** |
|  |  | **120–150** | 0.9890109890109890 | 0.9890109890109890 |
| **Perimeter** | **GABA** | **-60–0** | 1.0 | — |
|  |  | **0–30** | 0.2907092907092900 | 0.4152989867275580 |
|  |  | **30–60** | 0.8021978021978020 | 0.8021978021978020 |
|  |  | **60–90** | 0.3806193806193800 | 0.4273504273504270 |
|  |  | **90–120** | 0.1398601398601390 | 0.2331002331002330 |
|  |  | **120–150** | 0.0619380619380619 | 0.1238761238761230 |
|  | **glutamate** | **-60–0** | 1.0 | — |
|  |  | **0–30** | 0.0189810189810189 | 0.0632700632700632 |
|  |  | **30–60** | 0.0359640359640359 | 0.0899100899100899 |
|  |  | **60–90** | 0.0059940059940060 | **0.0299700299700299** |
|  |  | **90–120** | 0.0009990009990010 | **0.0099900099900100** |
|  |  | **120–150** | 0.3846153846153840 | 0.4273504273504270 |
| **Circularity** | **GABA** | **-60–0** | 1.0 | — |
|  |  | **0–30** | 0.3686313686313680 | 0.4095904095904090 |
|  |  | **30–60** | 0.3276723276723270 | 0.4095904095904090 |
|  |  | **60–90** | 0.3266733266733260 | 0.4095904095904090 |
|  |  | **90–120** | 0.1928071928071920 | 0.3213453213453210 |
|  |  | **120–150** | 0.5244755244755240 | 0.5244755244755240 |
|  | **glutamate** | **-60–0** | 1.0 | — |
|  |  | **0–30** | 0.0369630369630369 | 0.0739260739260739 |
|  |  | **30–60** | 0.0089910089910090 | **0.0299700299700299** |
|  |  | **60–90** | 0.0089910089910090 | **0.0299700299700299** |
|  |  | **90–120** | 0.0009990009990010 | **0.0099900099900100** |
|  |  | **120–150** | 0.0249750249750249 | 0.0624375624375624 |
| **Peak ∆F/F** | **GABA** | **-60–0** | 1.0 | — |
|  |  | **0–30** | 0.7932067932067930 | 0.8813408813408810 |
|  |  | **30–60** | 0.6193806193806190 | 0.8728771228771220 |
|  |  | **60–90** | 0.9460539460539460 | 0.9460539460539460 |
|  |  | **90–120** | 0.3456543456543450 | 0.8641358641358640 |
|  |  | **120–150** | 0.0079920079920080 | **0.0399600399600399** |
|  | **glutamate** | **-60–0** | 1.0 | — |
|  |  | **0–30** | 0.6983016983016980 | 0.8728771228771220 |
|  |  | **30–60** | 0.2247752247752240 | 0.7492507492507490 |
|  |  | **60–90** | 0.6513486513486510 | 0.8728771228771220 |
|  |  | **90–120** | 0.5284715284715280 | 0.8728771228771220 |
|  |  | **120–150** | 0.0009990009990010 | **0.0099900099900100** |
| **Fall time** | **GABA** | **-60–0** | 1.0 | — |
|  |  | **0–30** | 0.9150849150849150 | 0.9150849150849150 |
|  |  | **30–60** | 0.0789210789210789 | 0.1578421578421570 |
|  |  | **60–90** | 0.0549450549450549 | 0.1373626373626370 |
|  |  | **90–120** | 0.0079920079920080 | **0.0266400266400266** |
|  |  | **120–150** | 0.0009990009990010 | **0.0049950049950050** |
|  | **glutamate** | **-60–0** | 1.0 | — |
|  |  | **0–30** | 0.2287712287712280 | 0.3812853812853810 |
|  |  | **30–60** | 0.5644355644355640 | 0.6271506271506270 |
|  |  | **60–90** | 0.3876123876123870 | 0.4845154845154840 |
|  |  | **90–120** | 0.3426573426573420 | 0.4845154845154840 |
|  |  | **120–150** | — | — |
| **Rise time** | **GABA** | **-60–0** | 1.0 | — |
|  |  | **0–30** | 0.1798201798201790 | 0.1998001998001990 |
|  |  | **30–60** | 0.1088911088911080 | 0.1361138861138860 |
|  |  | **60–90** | 0.0109890109890109 | **0.0156985871271585** |
|  |  | **90–120** | 0.0019980019980020 | **0.0049950049950050** |
|  |  | **120–150** | 0.6643356643356640 | 0.6643356643356640 |
|  | **glutamate** | **-60–0** | 1.0 | — |
|  |  | **0–30** | 0.0109890109890109 | **0.0156985871271585** |
|  |  | **30–60** | 0.0049950049950050 | **0.0099900099900100** |
|  |  | **60–90** | 0.0009990009990010 | **0.0033300033300033** |
|  |  | **90–120** | 0.0009990009990010 | **0.0033300033300033** |
|  |  | **120–150** | 0.0009990009990010 | **0.0033300033300033** |
| **Decay time constant** | **GABA** | **-60–0** | 1.0 | — |
|  |  | **0–30** | 0.8951048951048950 | 0.8951048951048950 |
|  |  | **30–60** | 0.1318681318681310 | 0.3216783216783210 |
|  |  | **60–90** | 0.1608391608391600 | 0.3216783216783210 |
|  |  | **90–120** | 0.0199800199800199 | 0.0666000666000666 |
|  |  | **120–150** | — | — |
|  | **glutamate** | **-60–0** | 1.0 | — |
|  |  | **0–30** | 0.3766233766233760 | 0.5380333951762520 |
|  |  | **30–60** | 0.7402597402597400 | 0.8225108225108220 |
|  |  | **60–90** | 0.3686313686313680 | 0.5380333951762520 |
|  |  | **90–120** | 0.4775224775224770 | 0.5969030969030960 |
|  |  | **120–150** | — | — |
| **Duration (10% peak)** | **GABA** | **-60–0** | 1.0 | — |
|  |  | **0–30** | 0.5714285714285710 | 0.7842157842157840 |
|  |  | **30–60** | 0.6273726273726270 | 0.7842157842157840 |
|  |  | **60–90** | 0.9650349650349650 | 0.9650349650349650 |
|  |  | **90–120** | 0.8731268731268730 | 0.9650349650349650 |
|  |  | **120–150** | — | — |
|  | **glutamate** | **-60–0** | 1.0 | — |
|  |  | **0–30** | 0.0409590409590409 | 0.0999000999000999 |
|  |  | **30–60** | 0.0499500499500499 | 0.0999000999000999 |
|  |  | **60–90** | 0.0239760239760239 | 0.0799200799200799 |
|  |  | **90–120** | 0.0639360639360639 | 0.1065601065601060 |
|  |  | **120–150** | — | — |

**Statistics for Extended Data Fig. 4a: Fold change in individual Ca^2+^ events features compared to baseline.** Two-sided permutation testing was used to identify time bins with changes in event features compared to baseline. *p*-values were adjusted across tested time bins using the Benjamini-Hochberg procedure to obtain *q*-values. *q*-values < 0.05 are bold.

**Supplementary Table 13.**

| **Propagation direction** | **NT** | **Time relative to uncaging** | ***p*-value** | ***q*-value** |
| --- | --- | --- | --- | --- |
| **Growth toward pia** | **GABA** | **-60–0** | 1.0 | — |
|  |  | **0–30** | 0.4575424575424570 | 0.6978735550164120 |
|  |  | **30–60** | 0.3976023976023970 | 0.6978735550164120 |
|  |  | **60–90** | 0.5764235764235760 | 0.7159507159507160 |
|  |  | **90–120** | 0.7182817182817180 | 0.7182817182817180 |
|  |  | **120–150** | 0.4885114885114880 | 0.6978735550164120 |
|  | **glutamate** | **-60–0** | 1.0 | — |
|  |  | **0–30** | 0.0009990009990010 | **0.0099900099900100** |
|  |  | **30–60** | 0.0149850149850149 | **0.0374625374625374** |
|  |  | **60–90** | 0.0049950049950050 | **0.0166500166500166** |
|  |  | **90–120** | 0.0019980019980020 | **0.0099900099900100** |
|  |  | **120–150** | 0.6443556443556440 | 0.7159507159507160 |
| **Growth away from pia** | **GABA** | **-60–0** | 1.0 | — |
|  |  | **0–30** | 0.6063936063936060 | 0.6063936063936060 |
|  |  | **30–60** | 0.1988011988011980 | 0.2840017125731410 |
|  |  | **60–90** | 0.5224775224775220 | 0.5805305805305800 |
|  |  | **90–120** | 0.2367632367632360 | 0.2959540459540450 |
|  |  | **120–150** | 0.0529470529470529 | 0.1323676323676320 |
|  | **glutamate** | **-60–0** | 1.0 | — |
|  |  | **0–30** | 0.0179820179820179 | 0.0899100899100899 |
|  |  | **30–60** | 0.0929070929070929 | 0.1858141858141850 |
|  |  | **60–90** | 0.0139860139860139 | 0.0899100899100899 |
|  |  | **90–120** | 0.0319680319680319 | 0.1065601065601060 |
|  |  | **120–150** | 0.1368631368631360 | 0.2281052281052280 |
| **Growth right** | **GABA** | **-60–0** | 1.0000000000000000 | — |
|  |  | **0–30** | 0.6513486513486510 | 0.7237207237207230 |
|  |  | **30–60** | 0.4195804195804190 | 0.5994005994005990 |
|  |  | **60–90** | 0.8341658341658340 | 0.8341658341658340 |
|  |  | **90–120** | 0.3056943056943050 | 0.5094905094905090 |
|  |  | **120–150** | 0.0119880119880119 | 0.0599400599400599 |
|  | **glutamate** | **-60–0** | 1.0 | — |
|  |  | **0–30** | 0.0009990009990010 | **0.0099900099900100** |
|  |  | **30–60** | 0.0609390609390609 | 0.1523476523476520 |
|  |  | **60–90** | 0.0229770229770229 | 0.0765900765900765 |
|  |  | **90–120** | 0.0829170829170829 | 0.1658341658341650 |
|  |  | **120–150** | 0.6493506493506490 | 0.7237207237207230 |
| **Growth left** | **GABA** | **-60–0** | 1.0 | — |
|  |  | **0–30** | 0.1148851148851140 | 0.1914751914751910 |
|  |  | **30–60** | 0.3736263736263730 | 0.4895104895104890 |
|  |  | **60–90** | 0.3916083916083910 | 0.4895104895104890 |
|  |  | **90–120** | 0.4835164835164830 | 0.5372405372405370 |
|  |  | **120–150** | 0.0609390609390609 | 0.1298701298701290 |
|  | **glutamate** | **-60–0** | 1.0 | — |
|  |  | **0–30** | 0.0569430569430569 | 0.1298701298701290 |
|  |  | **30–60** | 0.0479520479520479 | 0.1298701298701290 |
|  |  | **60–90** | 0.0389610389610389 | 0.1298701298701290 |
|  |  | **90–120** | 0.0649350649350649 | 0.1298701298701290 |
|  |  | **120–150** | 0.9230769230769230 | 0.9230769230769230 |
| **Shrinking away from pia** | **GABA** | **-60–0** | 1.0 | — |
|  |  | **0–30** | 0.6213786213786210 | 0.7170607170607170 |
|  |  | **30–60** | 0.4595404595404590 | 0.6564863707720850 |
|  |  | **60–90** | 0.9120879120879120 | 0.9120879120879120 |
|  |  | **90–120** | 0.1958041958041950 | 0.3916083916083910 |
|  |  | **120–150** | 0.1328671328671320 | 0.3321678321678320 |
|  | **glutamate** | **-60–0** | 1.0 | — |
|  |  | **0–30** | 0.0809190809190809 | 0.3321678321678320 |
|  |  | **30–60** | 0.0309690309690309 | 0.3096903096903090 |
|  |  | **60–90** | 0.2447552447552440 | 0.4079254079254070 |
|  |  | **90–120** | 0.1268731268731260 | 0.3321678321678320 |
|  |  | **120–150** | 0.6453546453546450 | 0.7170607170607170 |
| **Shrinking away from deeper layers** | **GABA** | **-60–0** | 1.0 | — |
|  |  | **0–30** | 0.6793206793206790 | 0.7472527472527470 |
|  |  | **30–60** | 0.2237762237762230 | 0.4475524475524470 |
|  |  | **60–90** | 0.6083916083916080 | 0.7472527472527470 |
|  |  | **90–120** | 0.7472527472527470 | 0.7472527472527470 |
|  |  | **120–150** | 0.0339660339660339 | 0.1898101898101890 |
|  | **glutamate** | **-60–0** | 1.0 | — |
|  |  | **0–30** | 0.0569430569430569 | 0.1898101898101890 |
|  |  | **30–60** | 0.3356643356643350 | 0.5594405594405590 |
|  |  | **60–90** | 0.0419580419580419 | 0.1898101898101890 |
|  |  | **90–120** | 0.2097902097902090 | 0.4475524475524470 |
|  |  | **120–150** | 0.4985014985014980 | 0.7121449978592830 |
| **Shrinking away from right** | **GABA** | **-60–0** | 1.0000000000000000 | 1.0000000000000000 |
|  |  | **0–30** | 0.6383616383616380 | 0.6383616383616380 |
|  |  | **30–60** | 0.3776223776223770 | 0.5394605394605390 |
|  |  | **60–90** | 0.4615384615384610 | 0.5769230769230760 |
|  |  | **90–120** | 0.1898101898101890 | 0.3796203796203790 |
|  |  | **120–150** | 0.2517482517482510 | 0.4195804195804190 |
|  | **glutamate** | **-60–0** | 1.0 | — |
|  |  | **0–30** | 0.0799200799200799 | 0.3796203796203790 |
|  |  | **30–60** | 0.1718281718281710 | 0.3796203796203790 |
|  |  | **60–90** | 0.1528471528471520 | 0.3796203796203790 |
|  |  | **90–120** | 0.1018981018981010 | 0.3796203796203790 |
|  |  | **120–150** | 0.5754245754245750 | 0.6383616383616380 |
| **Shrinking away from left** | **GABA** | **-60–0** | 1.0 | — |
|  |  | **0–30** | 0.6963036963036960 | 0.8191808191808190 |
|  |  | **30–60** | 0.1238761238761230 | 0.2397602397602390 |
|  |  | **60–90** | 0.7372627372627370 | 0.8191808191808190 |
|  |  | **90–120** | 0.2047952047952040 | 0.2925645782788640 |
|  |  | **120–150** | 0.1438561438561430 | 0.2397602397602390 |
|  | **glutamate** | **-60–0** | 1.0 | — |
|  |  | **0–30** | 0.0849150849150849 | 0.2397602397602390 |
|  |  | **30–60** | 0.0499500499500499 | 0.2397602397602390 |
|  |  | **60–90** | 0.0179820179820179 | 0.1798201798201790 |
|  |  | **90–120** | 0.1348651348651340 | 0.2397602397602390 |
|  |  | **120–150** | 0.8341658341658340 | 0.8341658341658340 |

**Statistics for Extended Data Fig. 4b: Change in probability of Ca^2+^ events growing or shrinking in the indicated direction compared to baseline.** Two-sided permutation testing was used to identify time bins with changes in propagation probability compared to baseline. *p*-values were adjusted across tested time bins using the Benjamini-Hochberg procedure to obtain *q*-values. *q*-values < 0.05 are bold.

**Supplementary Table 14.**

| **Dataset** | **Round** | **Time relative to uncaging** | ***p*-values (raw)** | ***p*-values (adjusted)** |
| --- | --- | --- | --- | --- |
| Multi-round glutamate uncaging | Round 1 | 0–30 | 0.107389261073893 | 0.482586244333393 |
|  |  | 30–60 | 0.497850214978502 | 1.86436755938545 |
|  |  | 60–90 | 0.373962603739626 | 1.48280689599646 |
|  |  | 90–120 | 0.101789821017898 | 0.482586244333393 |
|  |  | 120–150 | 0.613638636136386 | 2.17703012457662 |
|  |  | 150–180 | 0.00779922007799220 | **0.0438102037453499** |
|  |  | 180–210 | 0.0619938006199380 | 0.321447648782449 |
|  |  | 210–240 | 0.00389961003899610 | **0.0238964747701909** |
|  |  | 240–270 | 0.135986401359864 | 0.572902664362268 |
|  |  | 270–300 | 0.00119988001199880 | **0.00898670846058460** |
|  |  | 300–330 | 0.000199980001999800 | **0.00192572324155384** |
|  |  | 330–360 | 0.000799920007999200 | **0.00674003134543845** |
|  |  | 360–390 | 0.00289971002899710 | **0.0195460909017715** |
|  |  | 390–420 | 9.99900009999000e-05 | **0.00112333855757308** |
|  |  | 420–450 | 9.99900009999000e-05 | **0.00112333855757308** |
|  |  | 450–480 | 9.99900009999000e-05 | **0.00112333855757308** |
|  |  | 480–510 | 9.99900009999000e-05 | **0.00112333855757308** |
|  |  | 510–540 | 9.99900009999000e-05 | **0.00112333855757308** |
|  |  | 540–570 | 9.99900009999000e-05 | **0.00112333855757308** |
|  | Round 2 | 0–30 | 0.104989501049895 | 0.505502350907884 |
|  |  | 30–60 | 0.379962003799620 | 1.58866503536305 |
|  |  | 60–90 | 0.679432056794321 | 2.41044805222391 |
|  |  | 90–120 | 0.561243875612439 | 2.10176644121922 |
|  |  | 120–150 | 0.363463653634637 | 1.58866503536305 |
|  |  | 150–180 | 0.0125987401259874 | 0.0943604388361383 |
|  |  | 180–210 | 0.0209979002099790 | 0.141540658254208 |
|  |  | 210–240 | 0.400659934006599 | 1.58866503536305 |
|  |  | 240–270 | 0.0349965003499650 | 0.214455542809405 |
|  |  | 270–300 | 0.103689631036896 | 0.505502350907884 |
|  |  | 300–330 | 0.0536946305369463 | 0.301616402708371 |
|  |  | 330–360 | 0.000199980001999800 | **0.00168500783635961** |
|  |  | 360–390 | 9.99900009999000e-05 | **0.000962861620776922** |
|  |  | 390–420 | 9.99900009999000e-05 | **0.000962861620776922** |
|  |  | 420–450 | 9.99900009999000e-05 | **0.000962861620776922** |
|  |  | 450–480 | 9.99900009999000e-05 | **0.000962861620776922** |
|  |  | 480–510 | 9.99900009999000e-05 | **0.000962861620776922** |
|  |  | 510–540 | 9.99900009999000e-05 | **0.000962861620776922** |
|  |  | 540–570 | 9.99900009999000e-05 | **0.000962861620776922** |
|  | Round 3 | 0–30 | 0.684231576842316 | 2.71306085275502 |
|  |  | 30–60 | 0.654234576542346 | 2.71306085275502 |
|  |  | 60–90 | 0.806319368063194 | 3.01953404275643 |
|  |  | 90–120 | 0.357364263573643 | 1.85299015604593 |
|  |  | 120–150 | 0.244075592440756 | 1.49567422856502 |
|  |  | 150–180 | 0.354064593540646 | 1.85299015604593 |
|  |  | 180–210 | 0.622237776222378 | 2.71306085275502 |
|  |  | 210–240 | 0.904009599040096 | 3.20719070495311 |
|  |  | 240–270 | 0.0222977702229777 | 0.187878373754097 |
|  |  | 270–300 | 0.479252074792521 | 2.30749787419189 |
|  |  | 300–330 | 0.135886411358864 | 1.01774473316121 |
|  |  | 330–360 | 0.210678932106789 | 1.42012460448388 |
|  |  | 360–390 | 0.00909909009099090 | 0.0876204074906999 |
|  |  | 390–420 | 0.000599940005999400 | **0.00674003134543845** |
|  |  | 420–450 | 9.99900009999000e-05 | **0.00168500783635961** |
|  |  | 450–480 | 9.99900009999000e-05 | **0.00168500783635961** |
|  |  | 480–510 | 0.000499950004999500 | **0.00674003134543845** |
|  |  | 510–540 | 9.99900009999000e-05 | **0.00168500783635961** |
|  |  | 540–570 | 9.99900009999000e-05 | **0.00168500783635961** |
| RuBi-glutamate uncaging control | Round 1 | 0–30 | 0.982401759824018 | 3.53816171786332 |
|  |  | 30–60 | 0.778222177782222 | 3.08574493891456 |
|  |  | 60–90 | 0.0261973802619738 | 0.346630183479692 |
|  |  | 90–120 | 0.00819918008199180 | 0.276341285162977 |
|  |  | 120–150 | 0.392460753924608 | 1.83778188018955 |
|  |  | 150–180 | 0.997300269973003 | 3.53816171786332 |
|  |  | 180–210 | 0.408959104089591 | 1.83778188018955 |
|  |  | 210–240 | 0.601639836016398 | 2.53467303784395 |
|  |  | 240–270 | 0.0212978702129787 | 0.346630183479692 |
|  |  | 270–300 | 0.211778822117788 | 1.18961553246989 |
|  |  | 300–330 | 0.103589641035896 | 0.634788406715840 |
|  |  | 330–360 | 0.0359964003599640 | 0.346630183479692 |
|  |  | 360–390 | 0.0818918108189181 | 0.552008567191409 |
|  |  | 390–420 | 0.0743925607439256 | 0.552008567191409 |
|  |  | 420–450 | 0.00609939006099390 | 0.276341285162977 |
|  |  | 450–480 | 0.0521947805219478 | 0.439787045289859 |
|  |  | 480–510 | 0.0229977002299770 | 0.346630183479692 |
|  |  | 510–540 | 0.0346965303469653 | 0.346630183479692 |
|  |  | 540–570 | 0.267973202679732 | 1.38948338505962 |
|  | Round 2 | 0–30 | 0.0592940705929407 | 1.75240814981400 |
|  |  | 30–60 | 0.604039596040396 | 2.85187576303865 |
|  |  | 60–90 | 0.0238976102389761 | 1.61086749155979 |
|  |  | 90–120 | 0.121987801219878 | 1.75240814981400 |
|  |  | 120–150 | 0.916208379162084 | 3.25046880096066 |
|  |  | 150–180 | 0.847915208479152 | 3.17530365607323 |
|  |  | 180–210 | 0.755224477552245 | 2.99455627953510 |
|  |  | 210–240 | 0.137786221377862 | 1.75240814981400 |
|  |  | 240–270 | 0.155984401559844 | 1.75240814981400 |
|  |  | 270–300 | 0.676932306769323 | 2.85187576303865 |
|  |  | 300–330 | 0.207279272072793 | 1.99601213987056 |
|  |  | 330–360 | 0.479252074792521 | 2.69208085322388 |
|  |  | 360–390 | 0.116288371162884 | 1.75240814981400 |
|  |  | 390–420 | 0.341065893410659 | 2.55447187992117 |
|  |  | 420–450 | 0.324367563243676 | 2.55447187992117 |
|  |  | 450–480 | 0.666833316668333 | 2.85187576303865 |
|  |  | 480–510 | 0.419858014198580 | 2.57285378359055 |
|  |  | 510–540 | 0.604739526047395 | 2.85187576303865 |
|  |  | 540–570 | 0.381361863813619 | 2.57064795515023 |
|  | Round 3 | 0–30 | 0.593640635936406 | 2.66770440652454 |
|  |  | 30–60 | 0.869013098690131 | 3.36402453374550 |
|  |  | 60–90 | 0.287671232876712 | 2.66770440652454 |
|  |  | 90–120 | 0.0991900809919008 | 1.67152777366874 |
|  |  | 120–150 | 0.327267273272673 | 2.66770440652454 |
|  |  | 150–180 | 0.999100089991001 | 3.54454701071690 |
|  |  | 180–210 | 0.505849415058494 | 2.66770440652454 |
|  |  | 210–240 | 0.406459354064594 | 2.66770440652454 |
|  |  | 240–270 | 0.0353964603539646 | 1.19298554814261 |
|  |  | 270–300 | 0.515848415158484 | 2.66770440652454 |
|  |  | 300–330 | 0.357564243575642 | 2.66770440652454 |
|  |  | 330–360 | 0.0989901009899010 | 1.67152777366874 |
|  |  | 360–390 | 0.572942705729427 | 2.66770440652454 |
|  |  | 390–420 | 0.465453454654535 | 2.66770440652454 |
|  |  | 420–450 | 0.859514048595141 | 3.36402453374550 |
|  |  | 450–480 | 0.206879312068793 | 2.66770440652454 |
|  |  | 480–510 | 0.00809919008099190 | 0.545942538980515 |
|  |  | 510–540 | 0.431456854314569 | 2.66770440652454 |
|  |  | 540–570 | 0.898310168983102 | 3.36402453374550 |

**Statistics for Extended Data Fig. 7d: Change in event frequency in neighboring cells during multiple rounds of glutamate uncaging or in RuBi-glutamate uncaging controls.** Permutation testing used to identify time-points with changes in event frequency greater than chance for each condition. *p*-values corrected for multiple comparisons using Benjamini-Yekutieli procedure with FDR ≤ 0.05. Adjusted *p*-values < 0.05 are bold.
