# Supplementary material for: Network-level encoding of local neurotransmitters in cortical astrocytes
Source: Nature. 2024 Apr 17;629(8010):146–53. doi: 10.1038/s41586-024-07311-5 (PMC11062919; doi:10.1038/s41586-024-07311-5)
Supplement: Supplementary file 2 — Reporting Summary [file 41586_2024_7311_MOESM2_ESM.pdf]

nature portfolio

Corresponding author(s): Kira Poskanzer

Last updated by author(s): Feb 6, 2024

Reporting Summary

Nature Portfolio wishes to improve the reproducibility of the work that we publish. This form provides structure for consistency and transparency in reporting. For further information on Nature Portfolio policies, see our Editorial Policies and the Editorial Policy Checklist.

Statistics

For all statistical analyses, confirm that the following items are present in the figure legend, table legend, main text, or Methods section.

n/a	Confirmed	
☐	☒	The exact sample size (*n*) for each experimental group/condition, given as a discrete number and unit of measurement
☐	☒	A statement on whether measurements were taken from distinct samples or whether the same sample was measured repeatedly
☐	☒	The statistical test(s) used AND whether they are one- or two-sided<br>*Only common tests should be described solely by name; describe more complex techniques in the Methods section.*
☐	☒	A description of all covariates tested
☐	☒	A description of any assumptions or corrections, such as tests of normality and adjustment for multiple comparisons
☐	☒	A full description of the statistical parameters including central tendency (e.g. means) or other basic estimates (e.g. regression coefficient) AND variation (e.g. standard deviation) or associated estimates of uncertainty (e.g. confidence intervals)
☐	☒	For null hypothesis testing, the test statistic (e.g. *F*, *t*, *r*) with confidence intervals, effect sizes, degrees of freedom and *P* value noted<br>*Give P values as exact values whenever suitable.*
☒	☐	For Bayesian analysis, information on the choice of priors and Markov chain Monte Carlo settings
☒	☐	For hierarchical and complex designs, identification of the appropriate level for tests and full reporting of outcomes
☐	☒	Estimates of effect sizes (e.g. Cohen's *d*, Pearson's *r*), indicating how they were calculated

Our web collection on statistics for biologists contains articles on many of the points above.

Software and code

Policy information about availability of computer code

Data collection	2P imaging data were collected using PrairieView software (version 5) and confocal immunohistochemistry data were collected using MicroManager (2.0.0-gamma1).
Data analysis	Data analysis was done using AQuA 1 (downloaded 12/6/2019 for data in Fig.1–4, Ext. Data Fig. 1–6 and 8/12/2021 for data in Ext. Data Fig. 7), standard analyses were done in MATLAB (2018b) and Python (v3.8.18). Preprocessing of 2P imaging data was done using FIJI 2.0.0–2.14.0 with the plugin moco (03-18-2016_release), when appropriate. Processing of images from immunohistochemistry samples was done using FIJI/ImageJ (version 1.53c) with the plugin SynQuant (v1.2.8). All analysis code used for this study are available on the public repository Zenodo (doi.org/10.5281/zenodo.10681987).

For manuscripts utilizing custom algorithms or software that are central to the research but not yet described in published literature, software must be made available to editors and reviewers. We strongly encourage code deposition in a community repository (e.g. GitHub). See the Nature Portfolio guidelines for submitting code & software for further information.

Data

Policy information about availability of data

All manuscripts must include a data availability statement. This statement should provide the following information, where applicable:

- Accession codes, unique identifiers, or web links for publicly available datasets
- A description of any restrictions on data availability
- For clinical datasets or third party data, please ensure that the statement adheres to our policy

All data used for this study are available on the public repository Dryad (10.5061/dryad.83bk3jb0j). Ribosomal-mRNA expression in visual cortex astrocytes were obtained from the Farhy-Tselnicker et. al. publicly available dataset (https://www.ncbi.nlm.nih.gov/geo/query/acc.cgi?acc=GSE161398; Database: NCBI Gene Expression Omnibus; Identifier: GSE161398).

Research involving human participants, their data, or biological material

Policy information about studies with human participants or human data. See also policy information about sex, gender (identity/presentation), and sexual orientation and race, ethnicity and racism.

Reporting on sex and gender	n/a
Reporting on race, ethnicity, or other socially relevant groupings	n/a
Population characteristics	n/a
Recruitment	n/a
Ethics oversight	n/a

Note that full information on the approval of the study protocol must also be provided in the manuscript.

Field-specific reporting

Please select the one below that is the best fit for your research. If you are not sure, read the appropriate sections before making your selection.

☒ Life sciences ☐ Behavioural & social sciences ☐ Ecological, evolutionary & environmental sciences

For a reference copy of the document with all sections, see nature.com/documents/nr-reporting-summary-flat.pdf

Life sciences study design

All studies must disclose on these points even when the disclosure is negative.

Sample size	No statistical methods were used to predetermine sample size, but our sample sizes are similar to those reported in previous publications (Di Castro et al., Nat. Neurosci., 2011; Mariotti et al., Glia, 2016; Durkee et al., Glia, 2018; Nagai et al., Neuron, 2021; Pittolo et al., Cell Reports, 2022), and statistical significance was calculated using post-hoc tests.
Data exclusions	Ex vivo 2P Ca2+ imaging during bath application of receptor agonists: slices from mice overexpressing Cyto-GCaMP were excluded, as the fluorescent sensor showed limited dynamics in these slices.<br>Ex vivo 2P uncaging datasets: individual cells were excluded if the baseline event rate changed significantly, as described in methods (under '2P uncaging event-based analysis').<br>Ex vivo 2P repeated rounds of uncaging: individual recordings were excluded if the focal plane of the slice changed over the course of the 15 min recording (z-drift), as this could not be corrected for post hoc like shifts in x and y.
Replication	Experiments were repeated as indicated for each figure panel. Empirical findings were replicated across multiple fields-of-view (FOVs), multiple slices, and multiple animals and were successful. The total number of FOVs, slices, and animals is reported for each experiment. The replications are shown as individual dots in all panels in which their display would not hinder readability/legibility of the graph. For event detection with AQuA, detection parameters were kept consistent within datasets.
Randomization	Samples were allocated into experimental groups by expression of each fluorescent sensor. When imaging the same slice in response to multiple stimuli in sequence, the order of the stimuli was alternated between slices and across experimental days. Both male and female mice were used, in approximately equal numbers, and were randomly selected.
Blinding	For ex vivo imaging experiments in response to stimuli, blinding was not possible because the baseline period was the first half of the recording and the post-stim period was the second half of the recording so that each response could be compared to its own baseline activity. Blinding was not relevant for 2P uncaging of GABA and glutamate because the same cells/FOVs were exposed to the same exact stimulation (all factors remained constant: concentration of caged compounds, uncaging location, uncaging intensity, imaging parameters) and the order of NT was alternated between slices.

Reporting for specific materials, systems and methods

We require information from authors about some types of materials, experimental systems and methods used in many studies. Here, indicate whether each material, system or method listed is relevant to your study. If you are not sure if a list item applies to your research, read the appropriate section before selecting a response.

Materials & experimental systems

n/a	Involved in the study
☐	☒ Antibodies
☒	☐ Eukaryotic cell lines
☒	☐ Palaeontology and archaeology
☐	☒ Animals and other organisms
☒	☐ Clinical data
☒	☐ Dual use research of concern
☒	☐ Plants

Methods

n/a	Involved in the study
☒	☐ ChIP-seq
☒	☐ Flow cytometry
☒	☐ MRI-based neuroimaging

Antibodies

Antibodies used

α-connexin-43 (1:1500, rabbit, Sigma-Aldrich), α-GFP (1:3000, chicken, Abcam), α-mCherry (1:2000, rat, Thermo Fisher Scientific), α-rabbit Alexa Fluor 405, α-chicken Alexa Fluor 488, and α-rat Alexa Fluor 555 (all 1:1000, Thermo Fisher Scientific)

Validation

α-connexin-43 (rabbit, Sigma-Aldrich, C6219) has been validated by the manufacturer. The application statement on the Sigma-Aldrich website reads:
"Anti-Connexin-43 may be used in immunoblotting, immunocytochemistry and immunohistochemistry (frozen and formalin-fixed, paraffin-embedded tissues). Polyclonal antibodies reacting specifically with Cx43 may be applied in diverse cellular and molecular approaches to the study of gap junctions and their properties. A minimum working dilution of 1:8,000 is determined by immunoblotting using a whole extract from mouse brain. A minimum working dilution of 1:400 is determined by indirect immunofluorescent staining of acetone-fixed cultured baby hamster kidney (BHK). A minimum working dilution of 1:2,000 is determined by indirect immunofluorescent staining of rat heart. (Negative on rat liver sections). A minimum working dilution of 1:2,000 is determined by indirect immunoperoxidase staining of trypsin-digested,formalin-fixed, paraffin-embedded human or animal tissue."

α-GFP (chicken, Abcam, ab13970) has been validated by the manufacturer. The following validations are listed on the abcam website under "images":
"-Immunocytochemistry/ Immunofluorescence – Anti-GFP antibody: ab13970 staining GFP in GFP-transfected NIH/3T3 (Mouse embryo fibroblast cell line) cells. The cells were fixed with 4% formaldehyde (10 minutes) and then blocked in 1% BSA / 0.3M glycine in 0.1%PBS-Tween for 1 hour. The cells were then incubated with ab13970 at 1/2000 dilution overnight at +4°C followed by incubation with Goat Anti-Chicken IgY H&L (Alexa Fluor® 488) preadsorbed (ab150173), for 1 hour, at 1 µg/ml. Under identical experimental conditions, when compared to the basal level of GFP expression in transfected NIH/3T3 cells, the cells upon which ab13970 was applied gave a stronger signal in the 488 channel, indicating that ab13970 is binding to GFP and therefore eliciting signal amplification. ab13970 was also applied to non-GFP-transfected NIH/3T3 cells, which produced no positive staining, indicating specificity for GFP. Nuclear DNA was labeled with 1.43 µM DAPI (blue).
-Western blot - Anti-GFP antibody (ab13970): All lanes : Anti-GFP antibody (ab13970) at 1/2000 dilution (Diluent 1x TBS /4 hours at 4° C). Lane 1 : 3 µg of GFP plasmid overexpressed in mouse cardiomyocytes whole cell lysate with BSA / for 1 hour at room temperature. Lane 2 : 2 µg of GFP plasmid overexpressed in mouse cardiomyocytes whole cell lysate with BSA / for 1 hour at room temperature. Lane 3 : 1 µg of GFP plasmid overexpressed in mouse cardiomyocytes whole cell lysate with BSA / for 1 hour at room temperature. Lysates/proteins at 25 µg per lane. Blocking peptides at 5 % per lane. Secondary all lanes : Goat Anti-Chicken IgY H&L (Alexa Fluor® 594) preadsorbed (ab150176) at 1/5000 dilution. Performed under reducing conditions. Additional bands at: 25 kDa. We are unsure as to the identity of these extra bands. Exposure time: 30 seconds. Gel Running Conditions: Reduced Denaturing (15% PAGE). Detection method: Fluorescent Secondary Antibodies."

α-mCherry (rat, Thermo Fisher Scientific, M11217) has been validated by the manufacturer. The following validations are listed on the Thermo Fisher Scientific website along with 166 references:
"Western blot was performed using: mCherry Monoclonal Antibody (16D7) (Product #M11217) by loading whole cell extracts of untransfected and transiently transfected HEK-293E lysates: untransfected, 60 µg (Lane 1), empty vector control, 60 µg (Lane 2), H3 mCherry, 60 µg (Lane 3), H3-mCherry, 30 µg (Lane 4), H3-mCherry, 15 µg (Lane 5), H3-mCherry, 7.5 µg (Lane 6), H3-DsRed, 60 µg (Lane 7), H3-dTomato, 60µg (Lane 8) and p65-RFP, 60 µg (Lane 9) were electrophoresed using NuPAGE™ 4-12% Bis-Tris Protein Gel (Product # NP0322BOX). Resolved proteins were then transferred onto a nitrocellulose membrane (Product # IB23001) by iBlot® 2 Dry Blotting System (Product # IB21001). A ~43 kDa band corresponding to H3-mCherry and 87 kDa band corresponding to p65-RFP were observed in HEK293E transfected lysates on probing with the primary antibody (1 µg/mL) and detected by chemiluminescence with F(ab')2-Rabbit anti-Rat IgG (H+L) Secondary Antibody, HRP (Product # PA1-29927, 1:4000 dilution) using the iBright FL 1500 (Product #A44241)."

α-rabbit Alexa Fluor 405 (Thermo Fisher Scientific, A-31556) has been validated by the manufacturer. The following validations are listed on the Thermo Fisher Scientific website, along with 374 references:
"Immunofluorescence analysis of Goat anti-Rabbit IgG (H+L) Cross-Adsorbed Secondary Antibody, Alexa Fluor 405 (Product # A-31556) was performed using MCF 10A (positive model) and A-431 (negative model) cells stained with Vimentin Polyclonal Antibody (Product # PA5-27231). The cells were fixed with 4% paraformaldehyde for 10 minutes, permeabilized with 0.1% Triton™ X-100 for

10 minutes, blocked with 1% BSA for 1 hour and labeled with 2 µg/mL primary antibody for 3 hours at room temperature. Goat anti-Rabbit IgG (H+L) Cross-Adsorbed Secondary Antibody, Alexa Fluor 405 (Product # A-31556) in 0.1% BSA in PBS for 45 minutes at room temperature, was used for detection of Vimentin in the cytoplasm (Panel a: blue). Nuclei (Panel b: red) were stained with SYTOX™ Orange Nucleic Acid Stain (Product # S11368). F-actin was stained with Alexa Fluor® 488 Phalloidin (Product # A12379, 1:300) (Panel c: green). Panel d represents the composite image. The specificity of the secondary antibody was proved by the absence of signal in A-431 (negative model for vimentin) due to no primary antibody binding (Panel e). Nonspecific staining was not observed with secondary antibody alone (panel f). The images were captured at 20X magnification."

α-chicken Alexa Fluor 488 (Thermo Fisher Scientific, A-11039) has been validated by the manufacturer. The following validations are listed on the Thermo Fisher Scientific website, along with 2307 references:
"Mouse intestine cryosection showing basement membranes labeled with our chicken IgY anti-fibronectin antibody (Product # A21316) and the Alexa Fluor® 488 Goat Anti-Chicken IgG (Product # A-11039, green). Goblet cells and crypt cells were labeled with Alexa Fluor® 594 wheat germ agglutinin (Product # W11262, red). The microvillar brush border and smooth muscle layers were visualized with Alexa Fluor® 680 phalloidin (Product # A22286, pseudocolored purple). The section was counterstained with DAPI (Product # D1306, D3571, D21490, blue)."

α-rat Alexa Fluor 555 (Thermo Fisher Scientific, A-21434) has been validated by the manufacturer. The following validations are listed on the Thermo Fisher Scientific website, along with 625 references:
"Immunofluorescence analysis of Goat anti-Rat IgG (H+L) Secondary Antibody, Alexa Fluor® 555 was performed using A549 cells stained with alpha Tubulin (YL1/2) Rat Monoclonal Antibody (Product # MA1-80017). The cells were fixed with 4% paraformaldehyde for 10 minutes, permeabilized with 0.1% Triton™ X-100 for 10 minutes, blocked with 1% BSA for 1 hour and labeled with 2µg/mL Rat primary antibody for 3 hours at room temperature. Goat anti-Rat IgG (H+L) Secondary Antibody, Alexa Fluor® 555 (Product # A-21434) was used at a concentration of 2µg/mL in phosphate buffered saline containing 0.2% BSA for 45 minutes at room temperature, for detection of alpha Tubulin in the cytoplasm (Panel a: red). Nuclei (Panel b: blue) were stained with DAPI in SlowFade® Gold Antifade Mountant (Product # S36938). F-actin was stained with Alexa Fluor® 488 Phalloidin (Product # A12379), 1:300) (Panel c: green). Panel d represents the composite image. No nonspecific staining was observed with the secondary antibody alone (panel f), or with an isotype control (panel e). The images were captured at 60X magnification."

Animals and other research organisms

Policy information about studies involving animals; ARRIVE guidelines recommended for reporting animal research, and Sex and Gender in Research

Laboratory animals	Swiss Webster (postnatal day 15–39), Cx43fl/fl and Cx43fl/+ (postnatal day 28–42), EAAT2-tdT (postnatal day 14–31) and C57Bl/6 (2–4 months) mice were used for experiments. Animal housing rooms were kept at 68-74 degrees Fahrenheit and 30-70% humidity.
Wild animals	The study did not involve wild animals.
Reporting on sex	Male and female mice were used at roughly equal numbers in all experiments in this study, and mice were chosen at random.
Field-collected samples	The study did not involve field-collected samples.
Ethics oversight	All experimental procedures were approved by the UCSF Institutional Animal Care and Use Committee.

Note that full information on the approval of the study protocol must also be provided in the manuscript.

Plants

Seed stocks	n/a
Novel plant genotypes	n/a
Authentication	n/a
